# The mutational dynamics of short tandem repeats in large, multigenerational families

**DOI:** 10.1186/s13059-022-02818-4

**Published:** 2022-12-12

**Authors:** Cody J. Steely, W. Scott Watkins, Lisa Baird, Lynn B. Jorde

**Affiliations:** Department of Human Genetics, University of Utah School of Medicine, Salt Lake City, UT 84112 USA

**Keywords:** Short tandem repeats, Mutations, Pedigrees

## Abstract

**Background:**

Short tandem repeats (STRs) compose approximately 3% of the genome, and mutations at STR loci have been linked to dozens of human diseases including amyotrophic lateral sclerosis, Friedreich ataxia, Huntington disease, and fragile X syndrome. Improving our understanding of these mutations would increase our knowledge of the mutational dynamics of the genome and may uncover additional loci that contribute to disease. To estimate the genome-wide pattern of mutations at STR loci, we analyze blood-derived whole-genome sequencing data for 544 individuals from 29 three-generation CEPH pedigrees. These pedigrees contain both sets of grandparents, the parents, and an average of 9 grandchildren per family.

**Results:**

We use HipSTR to identify de novo STR mutations in the 2nd generation of these pedigrees and require transmission to the third generation for validation. Analyzing approximately 1.6 million STR loci, we estimate the empirical de novo STR mutation rate to be 5.24 × 10^−5^ mutations per locus per generation. Perfect repeats mutate about 2 × more often than imperfect repeats. De novo STRs are significantly enriched in *Alu* elements.

**Conclusions:**

Approximately 30% of new STR mutations occur within Alu elements, which compose only 11% of the genome, but only 10% are found in LINE-1 insertions, which compose 17% of the genome. Phasing these mutations to the parent of origin shows that parental transmission biases vary among families. We estimate the average number of de novo genome-wide STR mutations per individual to be approximately 85, which is similar to the average number of observed de novo single nucleotide variants.

**Supplementary Information:**

The online version contains supplementary material available at 10.1186/s13059-022-02818-4.

## Background

Short tandem repeats (STRs), or microsatellites, are 1–6 base pair (bp) motifs of repeating units of DNA. These loci make up approximately 3% of the human genome [1]. STRs are distributed throughout the genome and are located in both coding and non-coding regions [2]. STRs have recently been associated with gene expression, where length variation can regulate gene expression of nearby loci [3–5]. STR expansions are also known to contribute to a number of diseases, including amyotrophic lateral sclerosis [6, 7], Huntington disease [8], fragile X syndrome [9], and nearly 50 others (reviewed in [10–12]).

STRs have high mutation rates compared to other types of variants, including single nucleotide variants (SNVs) and indels [13–15]. The mutation rate for STRs can vary significantly depending on the motif length at the locus of interest [16]. Their high heterozygosity has made STRs a valuable tool in forensics. Typically, only 13 loci are needed to have high statistical power to distinguish among individuals [17]. Multiple mechanisms have been proposed to explain this high mutation rate, including unequal crossing over in meiosis, retrotransposition-mediated mechanisms, and strand-slippage during replication (reviewed in [18]). It is possible that each of these mechanisms contributes to the high mutation rate of STRs, but strand slippage is the mechanism proposed for generating most observed mutations in STR loci [19]. Generally, studies of STR mutation rates have analyzed a small number of loci [20] or have focused on loci on the Y chromosome [21–24]. While recent work has examined genome-wide STR mutations in a small number of individuals, or families [25–27] or in disease cohorts [28, 29], further analysis of STRs is needed to better understand their mutational dynamics in the genomes of healthy individuals.

Due to the repetitive structure of STRs and their high mutability, sequencing and genotyping these loci is difficult, especially using short-read sequencing data. Many tools have been created during the last decade to genotype and identify mutations at STRs and longer tandem repeats across the genome [25, 30–33]. Some of these tools are designed to detect STR expansions at disease-related loci, while others detect expansions and contractions of STRs genome-wide but are constrained by sequencing read length and the STR motif size.

The three-generation structure of the Centre d'Etude du Polymorphisme Humain (CEPH) pedigrees has been valuable for previous work on mutation rates of single nucleotide variants, mobile element insertions, and structural variants [34–37]. These data have also been used in analyses of the role of maternal age and DNA damage in generating germline mutations [38] and in examining the association between SNV mutation rate and longevity [39]. Here, we present pedigree-based empirical estimates of the rate of mutation, parent-of-origin transmission differences, interfamilial repeat length variation, and the distribution of new STR alleles for microsatellite loci throughout the genome using these well-characterized CEPH families.

## Results

We utilized whole-genome sequencing data from 544 individuals in 29 CEPH pedigrees. These pedigrees include three generations, generally with both sets of grandparents in the first generation, the parents in the second generation, and the grandchildren in the third generation. The average number of grandchildren in the third generation is approximately nine (ranging from 7 to 16). We analyzed de novo STR mutations in the second generation of these families, and the large number of individuals in the third generation allowed us to analyze and verify transmission of putative de novo mutations.

We used HipSTR to genotype and analyze STR loci throughout the genome. Other tools for STR genotyping exist, such as GangSTR [40] and ExpansionHunter [41, 42], but these tools generally attempt to genotype alleles that are longer than read length and provide less precise estimates of allele length. HipSTR genotypes each STR and provides the precise length of each allele, but it does not attempt to genotype STRs that are longer than the length of the sequencing read. The precise estimation of STR length is important for analyzing de novo mutations that may differ by a single repeat unit. We used a reference file containing more than 1.6 million defined STR loci (see the “Methods” section), each of which HipSTR attempted to genotype. We were only able to assay STRs that were present in the reference file. While it is likely that there are other unannotated STRs, the number of STRs in the HipSTR reference file exceeds the number of STRs presently annotated in the human genome reference sequence (hg19). On average, ~ 49% of STR loci passed our filtering criteria for members of the second generation (see Methods, and Additional file 1: Table S1) and could be examined for de novo mutation events. Loci that did not pass our filtering generally had low coverage, low posterior probability supporting the genotype, high level of PCR stutter, or a large number of flanking indels.

To assess the accuracy of the genotypes produced by HipSTR, we compared a subset of the genotypes to previously analyzed PCR-based genotypes in the CEPH families (see the “Methods” section). We compared the PCR-based genotypes at ten random loci in three families (363 total genotypes) to the genotypes generated by HipSTR. The filtered HipSTR genotypes matched 353 of the 363 previously generated STR genotypes for a concordance rate of 97.25%. These previously genotyped loci do not include mononucleotide repeats, which are more difficult to sequence and validate. To check specifically for genotype quality in the first generation, we compared the genotypes called by HipSTR with PCR-based genotypes for 23 loci in grandparents in the CEPH families. We find that 796 of 813 genotypes generated by HipSTR match the previously generated STR genotypes for a similar concordance rate of 97.9% for the first generation.

**Fig. 1:**
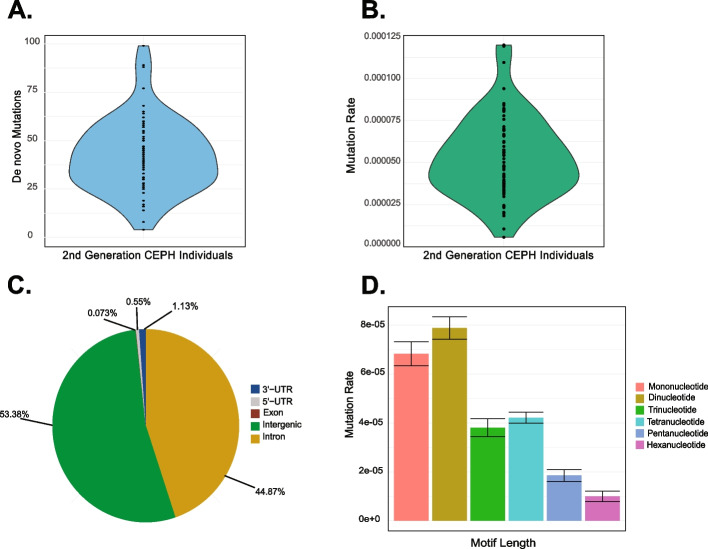
Number of de novo mutations and mutation rates among 68 individuals. **A** The number of de novo STR mutations identified in each individual from the second generation of the CEPH pedigrees. The mean number of identified mutations was ~ 42, with wide variation in the number of de novo mutations detected per individual. **B** The STR mutation rate for each individual in the second generation of the CEPH pedigrees. **C** The majority of de novo STR mutations were identified in intergenic regions (shown in green) and intronic regions (shown in yellow). Only two mutations overlap with exons. **D** Mutation rates for 2747 unique genotyped short tandem repeats with motif lengths from 1 bp (mononucleotide) to 6 bp (hexanucleotide). Mutation rates generally decrease as motif length increases, with some exceptions

In the second generation of each family, we identified de novo mutations at STR loci and then traced the transmission of the mutation to the third generation to ensure that it was a germline mutation. We analyzed 68 individuals in the second generation of 29 families (some large families have more than two individuals in the second generation). Those who were excluded were missing a parent in the first generation or had a parent who could not be analyzed by HipSTR (see the “Methods” section). Collectively, we were able to identify 5,249 putative de novo mutations in these individuals. We filtered these mutations to ensure that they were transmitted to at least two individuals in the third generation and filtered loci where the parent (in generation 2) without the de novo mutation was missing a genotype or shared the same genotype. We required that at least two individuals in the third generation had the de novo allele, similar to the filtering performed in the initial publication using HipSTR in a three-generation family [25]. Because the average number of grandchildren in the third generation is nine, the average false negative rate for detecting de novo mutations is only 0.0195 (see the “Methods” section). Approximately 20% of identified putative de novo mutations were not found in more than one individual in the third generation and were classified as false positive results, with generally decreasing frequency of false positives as the motif size increased (34.4%, 11.26%, 9.82%, 10.7%, 6.1%, 4.76% for mononucleotide – hexanucleotide, respectively). Approximately twenty-two percent of the total de novo mutations were shared with the other parent in the second generation, and for ~ 2% of these mutations, the other parent in the second generation had missing data. After filtering these loci, we identified 2859 de novo STR alleles in these individuals for an average of ~ 42 mutations per individual (Fig. 1A). There was a large amount of variation among individuals, with fewer than 10 mutations identified in some individuals and others having nearly 100; however, the number of new mutations discovered per individual follows a normal distribution (Shapiro–Wilk test, *p* = 0.08). The mutation rates calculated for STR loci show a pattern similar to the number of mutations per individual (Fig. 1B). The genome-wide mutation rates for STR loci that passed our filtering (which varied by trio) ranged from 5.58 × 10^−6^ to 1.2 × 10^−4^ with an average value of 5.24 × 10^−5^ mutations per locus per generation.

After identifying the de novo STR mutations in CEPH individuals, we examined the location of these mutations in the genome. Using the UCSC Genome Browser, we identified all exons, introns, 3′-UTRs, and 5′-UTRs in the genome (hg19). We then intersected the de novo STR’s position with each of these locations (Fig. 1C). The majority (53.38%) were found in intergenic regions. Slightly less than half (44.87%) were found in intronic regions, with a much smaller portion being found in UTRs. Only two mutations were found in exons: a trinucleotide (CCG) repeat mutation in *USP24* and a second trinucleotide (CGG) repeat mutation in *PHLPP1*. We compared the ratio of the number of observed and expected de novo events found in each of the five genomic features shown in Fig. 1C. De novo STR events are significantly underrepresented in the coding regions of exons (*p* < 1e − 6) but not in the 5′- and 3′-UTRs. In non-coding regions, slightly more de novo events were found in introns than expected by chance (Additional file 1: Table S2).

We also intersected the de novo STR locations with transposable element insertion locations in hg19 to determine how often transposable elements (*Alu* elements, LINE-1, and SVA) were potentially the source for new mutations (Additional file 1: Figure S1). These three families of transposable elements were selected for analysis because they are active in humans. Approximately 30% of the de novo STR mutations were found in recognized *Alu* elements, which can contain multiple poly(A) stretches. We found a smaller fraction of these mutations in LINE-1 (6%) and SVA (0.12%) elements. We compared the ratio of the number of observed and expected de novo events found in *Alu* elements (which compose 11% of the genome), LINE-1 (which compose 17% of the genome), and SVA (which compose 0.1% of the genome). De novo STR events were significantly overrepresented in *Alu* elements (*p* < 2.2e − 16) and significantly underrepresented in LINE-1 insertions (*p* < 2.2e − 16). The number of de novo events identified in SVA insertions was not significantly different from the expected value (*p* = 1) (Additional file 1: Table S3).

Next, we analyzed all unique de novo STR mutations passing all filters by their motif length. We divided the number of de novo mutations for each motif length by the number of STRs that passed our filters for that length. We found that STRs with shorter motif lengths generally had higher mutation rates than those with longer motif lengths (Fig. 1D). The mutation rates ranged from 9.99 × 10^−6^ for hexanucleotide repeats to 7.88 × 10^−5^ for dinucleotide repeats. Mononucleotide repeats had a slightly lower mutation rate than dinucleotide repeats at 6.82 × 10^−5^, but a smaller proportion of these passed our filtering methods.

We also compared the identified de novo STRs that were perfect repeats (e.g., “ATATATATAT”) against imperfect repeats, those with an interrupted repeat structure (e.g., “ATACATATAT”). Of the 2,747 unique STR loci with a de novo mutation, 2045 (~ 74.4%) were classified as perfect repeats, and 702 (~ 25.5%) were imperfect repeats by Tandem Repeats Finder (Table 1). We found that perfect repeats (0.00213 de novo mutations/total perfect repeat loci) were approximately twice as likely to mutate as imperfect repeats (0.00106 de novo mutations/ total imperfect repeat loci) (two-proportions *Z*-test, *p* < 0.00001).

**Table 1:** Perfect and imperfect de novo STRs. The number of de novo STR mutations that were defined as perfect and imperfect in our dataset. The “Total perfect” and “Total imperfect” columns show the number of genome-wide perfect and imperfect repeats identified. The fractions of perfect and imperfect loci with a de novo mutation were calculated by dividing the number of de novo mutations by the total number of perfect or imperfect repeats

	Perfect de novo	Imperfect de novo	Total perfect	Total imperfect	Fraction of perfect loci with de novo mutation	Fraction of imperfect loci with de novo mutation
Mono	788	145	615,604	216,599	0.0013	0.00067
Di	770	230	143,570	153,117	0.0054	0.0015
Tri	132	38	40,510	39,788	0.0033	0.00096
Tet	287	230	102,591	135,956	0.0028	0.0017
Penta	56	36	37,281	61,933	0.0015	0.00058
Hexa	12	23	20,956	52,125	0.00057	0.00044

**Fig. 2:**
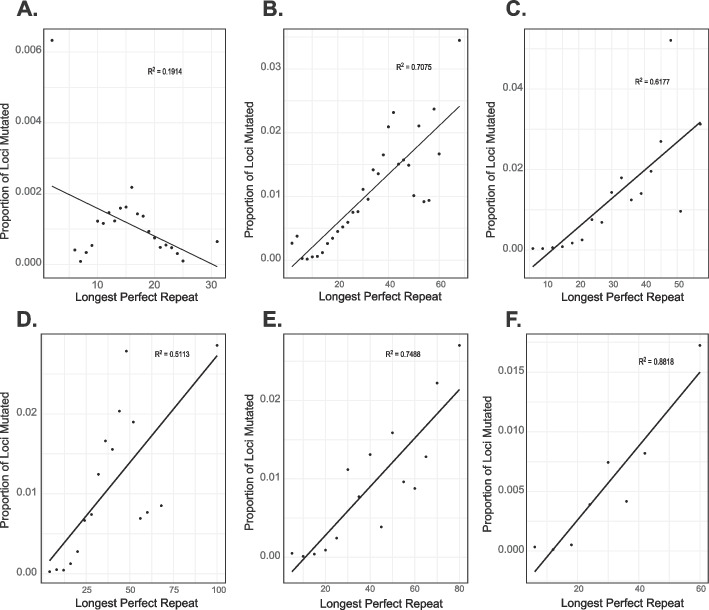
Relationship between the longest perfectly repeating segment of the STR and mutability. **A**–**F** show mononucleotides – hexanucleotides. The length of the perfectly repeating segment is shown on the *X*-axis, and the proportion of loci at a given length that were mutated are shown on the *Y*-axis

**Fig. 3:**
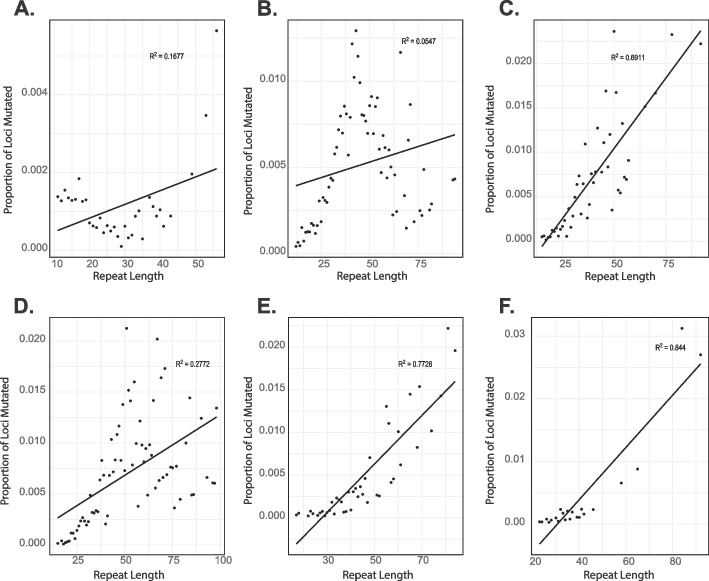
Relationship between total STR length and mutability. **A**–**F** show mononucleotides – hexanucleotides. The total length of the STR is shown on the X-axis, and the proportion of loci at a given length that were mutated are shown on the *Y*-axis

To better understand the factors that contribute to the mutation rate, we analyzed the relationship between the longest stretch of perfectly repeating sequence and the proportion of loci mutated. For dinucleotides – hexanucleotides we find that as the perfectly repeating segment increases in length, the locus is more mutable (Fig. 2B–F). Mononucleotides are an exception to this pattern as they increase in mutation rate as the longest perfectly repeating segment reaches ~ 15 bp, but the mutation rate begins to drop as this segment gets longer (Fig. 2A). This is likely caused by the difficulty in sequencing and genotyping these longer, low complexity loci. This pattern is similar when mononucleotide repeats within *Alu* elements are considered separately from those in other regions of the genome (Additional file 1: Figure S2 A and B). Additionally, we examined the relationship between total repeat length and the proportion of loci of that length that were mutated. We found that mononucleotides and dinucleotides show a weak relationship between repeat length and proportion of loci mutated (Fig. 3A, B); however, this relationship is much stronger as motif length increases (Fig. 3C–F). The pattern is similar when mononucleotide repeats within *Alu* elements are considered separately from those in other regions of the genome (Additional file 1: Figure S2 C and D). All motifs in Figs. 2 and 3 are shown with a linear trend line for consistency, but better-fitting trendlines for mononucleotide and dinucleotides can be found in Additional file: Figure S3.

To determine if de novo STR mutations are more likely to originate in males or females, we analyzed the sex of the grandparent transmitting the most probable haplotype with the de novo mutation event (see the “Methods” section). Of 2202 resolved haplotypes, 1117 de novo STR alleles were transmitted by males and 1085 were transmitted by females. This approximately 3% male transmission bias was not statistically significant (male/female ratio = 1.03, *p*-value ≥ 0.05, two-sided binomial test). We performed a power analysis to ensure that we had adequate power to detect a paternal bias. We estimate that we are sufficiently powered to detect a significant decrease in sex transmission bias for an effect size greater than ~ 10% (65% power to detect a 5% effect at an alpha of 0.05) (Additional file: Figure S4 A). Additionally, due to the difficulty of accurately sequencing and genotyping mononucleotide repeats, and because they compose ~ 34% of our de novo mutations, we also performed a power analysis that excluded the de novo mononucleotide repeats. This analysis shows a decrease in power (49% power to detect a 5% effect at an alpha of 0.05) (Additional file: Figure S4 B). The male–female difference in de novo mutation rates remained nonsignificant after removing the mononucleotide repeats from this analysis.

**Fig. 4:**
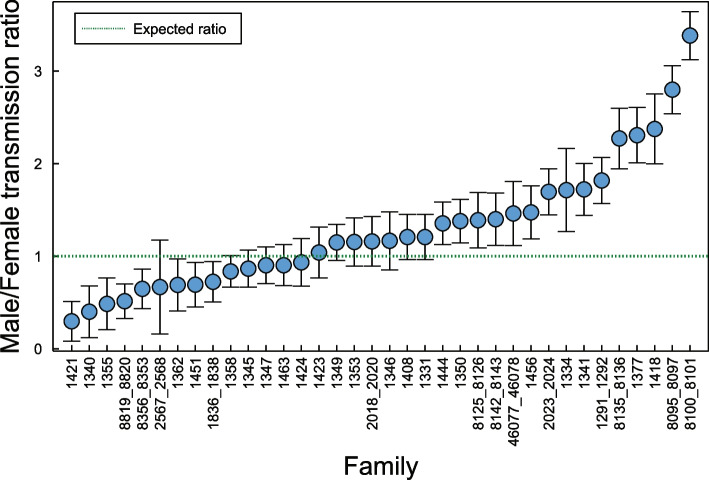
Observed male/female de novo STR transmission ratio by family. The ratio of male to female transmissions of de novo STR alleles is shown by family. The male/female transmission ratio ranged from 0.30 to 3.4. Of the 36 individual families within the 29 large CEPH pedigrees*, 22/36 (61%) have a higher-than-expected rate of de novo STR transmission by males, while 14/36 (39%) have a low-than-expected transmission rate. The male/female transmission ratios suggest a trend for a male transmission bias in the CEPH families. *Several CEPH families have an extended pedigree structure which were separated into non-extended pedigrees for the analysis (see the “Methods” section)

Individual families, however, varied in their male/female transmission ratios. Fifteen families (42%) had an elevated male/female transmission ratio (Fig. 4). Four families had a statistically significant transmission bias after correcting for multiple tests. Families 1421 and 8819_8820 showed a female transmission bias, and families 8095_8097 and 8100_8101 showed a male transmission bias (*p* < 0.05, Bonferroni corrected). In three of these four families, a two- to three-fold higher rate of de novo transmission in one grandparent accounted for the elevated ratio. This result suggests that some individuals transmit new STR mutations at an atypically high rate. We also analyzed the relationship between parental age and STR mutation rate but did not find a strong correlation for either paternal age (*r*^2^ = 0.044) or maternal age (*r*^2^ = 0.0071).

**Fig. 5:**
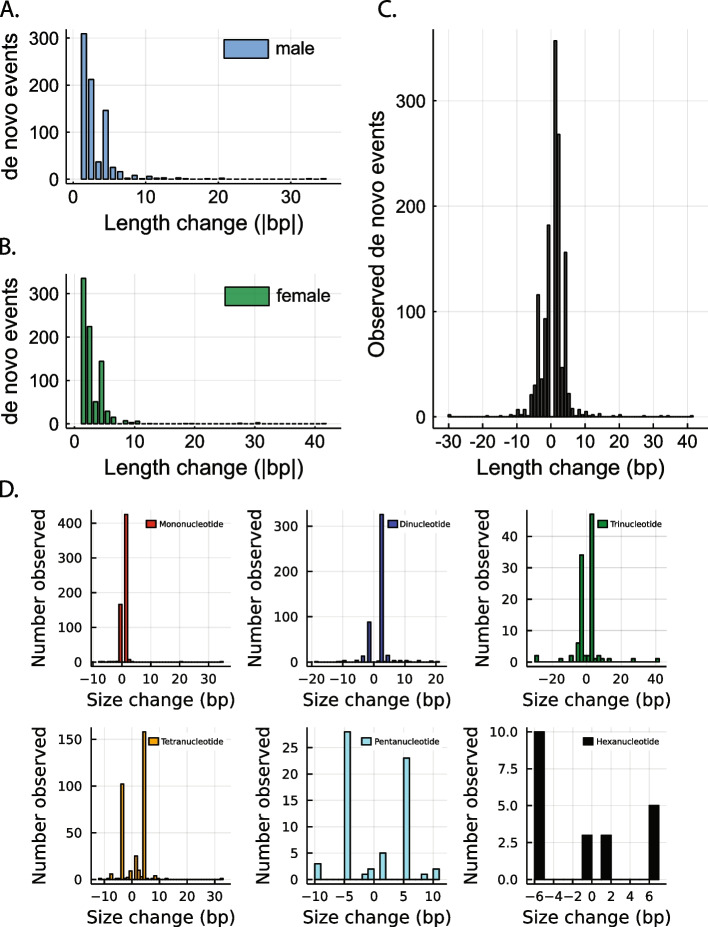
Distribution of length changes for phased de novo STRs. **A**, **B** The distribution of length differences between new and original phased STR alleles is similar among CEPH males and females. **C** Most de novo events are contractions and expansions of one repeat unit. **D** Panels show the distributions of transmitted phased-determined de novo STR alleles for each STR motif length in the CEPH families**.** In each case, the majority of the observed size changes are a single repeat unit, and most new mutations are a multiple of the original STR motif

We analyzed the spectrum of size differences between the original and the de novo STR alleles for 1388 mutations in which the transmitting haplotype and the size change in base pairs could be unambiguously identified. Except for three-base pair repeats, smaller mutations were generally more frequent than larger mutations, consistent with the overall pattern of observed mutations (Fig. 5A, B). There was not a significant difference between the length of de novo alleles in males and females (*p*-value ≥ 0.7, two-tailed *t*-test). Expansions were favored over contractions overall (*P* < 1e − 24, binomial test) (Fig. 5C). By class, the larger penta- and hexa-nucleotide repeats had slightly more contractions than expansions, but the differences between expansions and contractions were not significant (Fig. 5D, *P* > 0.5). Collectively, new STR alleles rarely differed by more than 6 bp from their original length, consistent with strand-slippage by a single repeat unit as the most common length change for all classes of microsatellites.

After determining the parent of origin for the mutations, we analyzed the change in size, as measured by the number of repeats, between the transmitting grandparent’s allele and the de novo allele in the parent explicitly by repeat motif size (Fig. 5D). The majority of the identified mutations show a single stepwise change, defined as a mutation resulting in a change of a single repeat motif (that is, a dinucleotide repeat expanding or contracting by two bases or a trinucleotide repeat expanding or contracting by three bases, etc.). This pattern is seen for each STR motif type examined, with the total number of events decreasing as motif size increases.

## Discussion

We used the unique structure of the CEPH pedigrees to determine the genome-wide mutation rate and the number of STR mutations inherited in 68 individuals from generation 2 in 29 3-generation families. Our analysis reveals a high degree of variability among families in the number of transmission-verified de novo STR mutations (see Fig. 1A) and in the mutation rate of STR loci (see Fig. 1B). This variation is similar to the pattern seen for single nucleotide variants in these families [35]; it may be due to individual differences in genetic backgrounds or differences in DNA repair efficacy. We compared the number of de novo STRs to the number of de novo single nucleotide variants transmitted by each individual and found no correlation between the number of mutations found (*r*^2^ = 4 × 10^−5^). We may not see a relationship between these values due to the small sample size included in our study or differing DNA repair mechanisms involved in these two mutation types.

Analyzing approximately 49% of all STR loci in the genome, we found an average of ~ 42 de novo STRs in the examined individuals and an average mutation rate of 5.24 × 10^−5^ per locus per generation. If we were able to assay all STRs across the genome, we estimate there to be an average of approximately 85 de novo STR mutations per individual. This estimate of de novo STR mutations in these individuals falls within the range of previous genome-wide estimates [21, 25]. However, our estimate is likely to be conservative. This is due in part to the limitations of HipSTR and short-read sequencing data. We are only able to confidently assess repeats that are smaller than the sequencing reads (~ 150 bp), permit flanking sequence to be mapped, and have reads that completely span the STR motif. We also removed alleles that were shared with the other parent or were not passed down to multiple grandchildren in the third generation. Some of the identified mutations that were passed down to fewer than two grandchildren may have been true de novo mutations or the product of mosaicism. While it is likely that some true de novo STRs were excluded because of this filtering criterion, this prevented a number of false positives from being included in the dataset. Additionally, few loci on the Y chromosome passed our filtering criteria, possibly due to the highly repetitive nature of the Y chromosome [43, 44]. While these filtering steps increased confidence in our genotyping, they also decreased sensitivity. Finally, the WGS data from the CEPH families are not PCR-free, which may have introduced some additional level of error into our analyses. PCR stutter may create products that are generally one repeat unit smaller than the target, leading to an incorrect repeat size being sequenced and genotyped.

The majority of de novo STR mutations were found in intergenic or intronic regions, with a very small proportion of these events occurring in exons and 3′- or 5′-UTRs (Fig. 1C), similar to previous findings of variability at these loci[26]. Only two of these mutations occur in exons, and unsurprisingly, they are both trinucleotide repeats. One of these mutations was found in *PHLPP1*, which has been associated with colorectal cancer [45], and the other was found in *USP24*, which has been associated with Parkinson’s disease [46]. These loci appear to be polymorphic within the families that pass our filtering criteria.

Examining non-exonic STRs, we found that many of the identified de novo STR mutations overlap with transposable elements (TEs) (Additional file 1: Figure S1). TEs have been proposed to act as seeds for microsatellites [47, 48] because of their poly(A) tails; furthermore, *Alu* elements have an A-rich region in the middle of the element. Approximately 30% of our de novo STRs overlap with *Alu* elements, likely in the poly(A) tail, so this may be an underappreciated source of origin for new STR loci. This finding supports recent work showing that many non-reference or rare tandem repeat loci are in close proximity to *Alu* elements [49]. We found fewer de novo STRs in L1s and SVAs, despite the fact that L1s compose a greater portion of the genome than *Alu* elements (reviewed in [50]). It is unclear if this difference is due to a lower number of copies of L1s throughout the genome, thus creating fewer potential seeds for microsatellites, or if some STRs in L1s were difficult to identify and include in the reference file. Alternatively, the genomic location of TE insertions may play a large role in the mutation rate of the associated STRs. *Alu* elements, particularly older insertions, have been shown to insert in more GC-rich regions of the genome, while L1 insertions have been found in more AT-rich regions [51, 52]. Because GC-rich regions of the genome accrue more mutations (as shown in yeast [53]), *Alu* elements may contain more STR mutations due to their genomic location rather than something unique about the insertion itself. Additionally, an analysis of poly(A) tail length of *Alu* elements and L1 insertions using Tandem Repeats Finder shows that *Alu* elements (mean =  ~ 21; median = 20) have longer identifiable poly(A) tracts than L1s (mean =  ~ 13; median =  ~ 11). These longer poly(A) tails may also contribute to the increased number of STR mutations in *Alu* elements. There are likely many factors influencing the relationship between STRs and TEs, and this relationship should be further investigated.

Although many studies have examined the mutation rates of different STR motifs, there appears to be no consensus on how mutation rate and motif size are related. Some studies show that dinucleotide repeats mutate more quickly than the longer tetranucleotide repeats [16, 54], while others show that tetranucleotide repeats mutate more quickly [20, 55]. In our dataset, the mutation rate generally decreases with increased motif length (Fig. 1D); however, there are exceptions to this pattern. Over all loci, the mononucleotide repeats examined in this study appear to mutate more slowly than the dinucleotide repeats. This is likely due to an under-sampling of the mononucleotide loci and sampling of only those loci that are smaller than read length. Given the low complexity of these regions, they are more difficult to sequence and confidently genotype. Many of these loci did not pass the filtering applied due to low-quality scores or a small number of reads spanning the repeat. Other mononucleotide repeats are located in the tail of transposable elements and are difficult to map accurately, likely contributing to the decreased number of repeats passing our filters. Due to these factors, as well as the fact that the WGS data are not PCR-free, it is possible that the mononucleotide mutation rate is less accurate than that of other classes of STRs. The second STR motif that does not follow this pattern is trinucleotide repeats, which have a slightly lower mutation rate than tetranucleotides. The trinucleotide mutation rate falls within the 90% CI of the best-fit curve for de novo allele size changes using all phased de novo mutations (Additional file 1: Figure S5), indicating that mutational steps of three base pairs are not exceptionally low compared to expectation. Relative to mono-, di-, tetra-, and pentanucleotide repeats [2], trinucleotide repeats are enriched in coding regions and may be mutationally constrained due to negative selection on new alleles. We also find that the frequency of observed de novo STR alleles decreases exponentially with an increasing number of repeats (Additional file 1: Figure S5).

Examining the structure and length of the de novo STRs, we found that perfect repeats mutate more quickly than imperfect repeats (Table 1). This has been found in previous work and is hypothesized to be due to increased replication slippage in these perfectly repeating regions [54, 55]. We also analyzed the relationship between mutability and the longest perfectly repeating segment, as well as repeat length, because these factors have been linked to mutation rates. We find that for dinucleotides – hexanucleotides, as the length of the perfectly repeating segment increases, the STR becomes more mutable (see Fig. 2). Mononucleotides do not follow this pattern, but it is likely that most of the longer perfectly repeating mononucleotide STRs have been filtered from our final data set due to low sequencing coverage or poor genotyping quality. Furthermore, it is possible that the decreasing number of STRs at greater lengths in the reference genome (Additional file 2: Table S4) could play a role in this trend for mononucleotides. A similar pattern is seen for dinucleotide repeats as the majority of these are smaller than 44 bases. We find a similar pattern for the total length of the STR, with longer repeat motifs showing increased mutability as the total length of the repeat increases (see Fig. 3). We also found a general trend for increasing allele length driven by single repeat length expansions in the mono- through tetranucleotide repeats, consistent with previous studies [55–57]. Overall, the results of this analysis match closely with previous work[21].

Previous studies of mutation dynamics have noted a male bias for de novo single nucleotide variants [35, 58, 59]. From our analysis, we identified slightly more male than female transmissions for all de novo STR mutations, but this excess was not significant. Among families, we find that more families show male transmission bias than female transmission bias (Fig. 4). Comparing the male and female transmissions within a single family shows that either males or females can have statistically significant excess transmissions, but also that these cases are infrequent. This finding is, again, quite similar to the results shown for single nucleotide variants in these families. We also note that our estimated error rate for STRdiff is ~ 5%, which may reduce our ability to identify a parent-of-origin effect if that effect is very small.

A previous genome-wide analysis of STRs in a single CEPH family found varying levels of male mutation bias per motif, and similar to our results, the level of male bias was slight[27]. Our power analysis suggests that we have adequate power to detect a signal of male transmission bias when all STRs are included, and the effect size is at least 10%. If there are many false positives in the mononucleotides, the signal may be obscured. While we did not see a different pattern when the mononucleotides were removed, our power to detect such a pattern was also decreased.

Due to the challenges and high false positive rate associated with genotyping STRs, we were unable to examine de novo mutations in the third generation of individuals within these families. This also prevented us from examining the effect of parental age (within a single family) on the mutation rate. Collection of the fourth generation of the CEPH pedigrees is now underway and will allow new analyses to better understand how parental age, genetic background, and differences within DNA repair genes play a role in altering mutation rates.

Comparing the size of the mutation to the motif length of the STR, we find that most of the mutations occur in a stepwise fashion (see Fig. 5). This mutation pattern has been noted in other studies [26, 60, 61]. Regardless of the repeat motif length, we found a rapidly decreasing number of mutations as the step size increased. In addition to stepwise changes, we find that small indels can occur within the repeat unit, particularly for hexanucleotide repeats. These were generally an increase or decrease of a single base within the repeat unit. Slippage has been the proposed mechanism for most single-step STR mutations, though larger STR mutations may be caused by other mechanisms, including unequal crossover, and should be further investigated [18].

## Conclusions

We were able to utilize the unique structure of the CEPH pedigrees to better understand the mutational dynamics of STR loci in healthy individuals. As sequencing technology (e.g., long-read sequencing) and computational methods for the detection and genotyping of STRs improve, precisely genotyping longer STRs will improve the estimate of the mutation rate. Future analyses of large pedigrees from diverse populations may uncover additional variation in STR mutation rates, as we have only examined families of European ancestry. Our study provides new perspectives on the dynamics of STR mutations and highlights the need for larger sample sizes and novel tools to investigate this underappreciated portion of the genome.

## Methods

### Sequencing data

Whole-genome sequencing (WGS) data were available from 599 individuals from 33 families (Sasani et al. 2019). The WGS data are not PCR-free. These genomes were sequenced to ~ 30 × coverage, with complete coverage data for each genome used in this study shown in Additional file 3:Table S5. Coverage data for each file were generated using covstats from the goleft package. These data are available with controlled access through dbGaP (phs001872.v1.p1).

### Short tandem repeat genotyping and basic filtering

HipSTR (version 0.6.2) [25] was used to genotype short tandem repeats (STRs) in the CEPH sequencing data. HipSTR was run on 29 families, though in some cases not all individuals from a family could be successfully run due to a known issue with HipSTR stemming from difficulty extracting certain filtering tags from BAM files. Most pedigrees contain all four grandparents in the first generation (only four pedigrees are missing one or more grandparents). Some families contain multiple offspring (parents) in the second generation, allowing for the analysis of multiple individuals in some of the 29 pedigrees. Splitting these extended pedigrees produced 36 family units used for some analyses. Each CEPH family was run individually using the default stutter model, as recommended in the HipSTR documentation. The GRCh37 STR reference file from HipSTR (which includes approximately 1.6 million loci) was used for all analyses. Filtering methods included with HipSTR and DumpSTR from TRTools (version 3.0.0) [62] were used to filter the genotypes generated by HipSTR. Specifically, we filtered these genotypes for loci with a minimum quality score > 0.9, maximum flanking indels < 0.15, maximum call stutter < 0.15, minimum call depth of 10, maximum call depth of 1000. All loci overlapping segmental duplications in the genome were removed. With these filtering criteria, we retained loci that were confidently genotyped, did not contain too many flanking indels, and showed little evidence of stutter artifacts in the repeat.

### Validation of genotypes

STR genotypes were previously generated in the 1990s for 360 markers for individuals in the CEPH pedigrees as part of the Human Genome Project. STR loci were amplified by PCR, and STR genotypes were detected and visualized using an Automated Hybridization and Imaging Instrument (AHII) by modifying the methodology previously used to generate high throughput de novo sequence data [63]. These loci were identified in hg19 using the UCSC Genome Browser; then the genotypes coded by HipSTR were compared to the genotypes previously coded using AHII. A list of the loci and genotypes generated by AHII and HipSTR are shown in Additional file 4: Table S6. In total, we analyzed 10 loci in multiple families, allowing for the comparison of 363 genotype calls between the two methods. For the comparison that included only grandparents, we analyzed a total of 23 loci in multiple families. This included 813 genotypes called by both methods. These genotype comparisons are shown in Additional file 5: Table S7.

### *Filtering for *de novo* mutations*

After filtering the genotypes generated with HipSTR, the genotypes for individuals in the second generation of the CEPH families were compared to those of their parents (generation 1) to identify potential de novo mutations. For a mutation to be considered for further analysis, we required that at least 10 reads spanned the de novo allele in the individual in the second generation. This decreased the number of loci that could be considered for analysis, but provided greater confidence in the called genotype.

Next, each potential de novo STR that met these criteria in the second generation was compared to the third generation to ensure that the de novo allele was transmitted to multiple individuals (at least two) in the third generation (similar to Willems et al. 2017). Given the high mutation rate of STR loci, requiring that at least two individuals inherited the de novo allele increased our confidence in the successful transmission of the de novo allele. In the average family in this study with nine grandchildren the probability of transmitting a de novo mutation to zero or one child in the third generation is 0.0195 (binomial test). With our 5249 putative de novo mutations, we would expect only 102 false negatives (0.0195 × 5249). Further, to ensure the de novo allele was transmitted from the individual in which it was identified, we removed from consideration all de novo STRs that were shared with the other parent (similar methodology was also used in [25]). To calculate the STR mutation rate, we divided the number of de novo mutations by the total number of STRs that passed our filters for each trio.

### Identifying perfect and imperfect repeats

To identify which STR loci were perfect and which were imperfect, we used Tandem Repeats Finder [64] (Version 4.09.1). BEDTOOLS [65] was used to get sequence data for each of the approximately 1.6 million STR loci included in the HipSTR reference file. The sequence data were analyzed with Tandem Repeats Finder with a minimum score requirement of 15 to ensure that even the short repeats included in the HipSTR reference file could be accurately identified. Each motif size (mononucleotide − hexanucleotide) was run separately to be sure we were identifying the correct repeat. Those repeats that had a perfect score for “Percent Matches” in the output file were considered to be perfect repeats. All other repeats were considered to be imperfect. We then used BEDTOOLS to intersect the loci in which we identified de novo STR mutations with the location of the perfect and imperfect repeats.

### *Genomic Location of *de novo* mutations*

After identifying de novo mutations in STRs, we intersected the location of these mutations with exons, introns, 5′-UTRs, and 3′-UTRs using BEDTOOLS [65]. We used the UCSC Genome Browser to find the location of genes in hg19. The different regions of genes were run separately to determine if the de novo STRs intersected with any component of a gene. A similar procedure was used to find the location of active transposable elements (*Alu* elements, L1, and SVA) in hg19. These loci were intersected with the identified de novo mutations to determine the frequency with which transposable elements were the sites of these mutations.

### Identification of Poly(A) tails in *Alu* elements and L1s.

To determine the length of identifiable poly(A) tails in *Alu* elements and L1s, we obtained fasta files of the sequences for each insertion (with an additional 40 bp of flanking sequence on the 3′ end) in hg19 from the UCSC Genome Browser. Short tandem repeats in each element were identified with Tandem Repeats Finder. The results were then filtered to only include those that were found near the end of the insertion and had a repeat motif of “A.” From the filtered results, the mean and median length of the identifiable poly(A) tails were determined.

### STRdiff

STRdiff was used to evaluate the characteristics of de novo STR mutations found in parents of the CEPH families. This program leverages the three-generation structure of the CEPH pedigrees to infer the sex and haplotype of the grandparent transmitting the de novo STR allele. It also infers the size change, in base pairs, between the original and the de novo alleles.

Input to STRdiff is a variant call format (vcf) file containing two sets of grandparents, two parents, and all offspring. A region surrounding the de novo STR is first extracted and then phased in all possible trios within the family using the Beagle software package [66]. Because the de novo STR is a mutational event that creates a misinherited allele, the de novo allele cannot be phased directly. Instead, the haplotype(s) carrying the novel STR allele are first identified in multiple offspring. A consensus haplotype is created from all offspring haplotypes that carry the de novo allele. Using a consensus haplotype helps to reduce mismatches caused by rare alleles, sequencing/genotyping errors, and inferred recombination events. The consensus haplotype is then compared to each of the four-phased grandparental chromosomes of the parent that harbors the de novo mutation. The likelihood of a match to each grandparental chromosome is calculated as the fraction of alleles contained on each grandparental haplotype that match the offspring consensus haplotype bearing the de novo STR allele. These match probabilities are used to identify the most likely grandparental chromosome with the original STR allele that produced the mutational event. To evaluate the confidence in each prediction, the difference between the highest and the next best match likelihood is provided as a unique solution score (uss). A default uss score of 10 filters a small percentage of reads. The uss score may be changed by the user to accommodate varying levels of genomic relatedness in a data set.

Depending on the chromosomal location and family, regional haplotypes may be highly similar. To improve the number of de novo STRs for which a transmitting grandparental haplotype could be reliably identified, STRdiff was run over a range of haplotype sizes (10, 20, …, 300 kb). By using this broad range of haplotypes, a probable haplotype solution was found for 2361 of 2456 (96%) de novo STRs. Of these 2361 haplotypes, 2202 (93%) were uniquely resolved from other haplotypes at a minimum of ≥ 10% of all polymorphic sites found along the length of the haplotype. Additionally, we tested the concordance between haplotypes constructed directly from WGS sequence and those based on 1.1 M SNPs common Illumina array SNPs extracted from the WGS sequence. There was > 94% concordance for sex assignment for the transmitting grandparent. Assignments that differed were most often due to a high similarity among parental and grandparental haplotypes at that locus and minor phasing differences among the inferred haplotypes.

To obtain a more rigorous independent estimate of the accuracy of STRdiff, a subset of 24 de novo transmission events were examined in IGV [67]. These loci, along with details of the STRdiff prediction for each, are shown in Additional File 1: Table S8. The examined loci were selected randomly, but we required that these loci had sufficient read depth, low stutter at the STR locus, and usually a nearby SNP to allow for independent read-based phasing. Mononucleotide repeats were particularly difficult to analyze through IGV. The STRdiff and IGV read-based phasing predictions for the parent transmitting the de novo STR allele matched in 23/24 (> 95%) of the examined loci. The allele size changes, when predicted by STRdiff, were correct for 19/21 (~ 90%) loci. The IGV images associated with each locus are shown in Additional file 1: Figure S6.

### Size change of STR mutations

The stepwise change for each class of STR repeat (mono, di, …) was calculated in STRdiff using information from the vcf file. The sequence for the transmitting grandparent’s allele and the de novo allele in the offspring was obtained directly from the vcf file. The absolute value of the difference in base pairs between these alleles was divided by the allele class size to determine the number of steps. Repeat size changes could not always be resolved due to similarity in haplotypes and STR homozygosity in the grandparents, parents, or offspring. In total, repeat size changes were determined for 1533 mutational events.

## Supplementary Information

**Additional file 1.**

**Additional file 2.**

**Additional file 3.**

**Additional file 4.**

**Additional file 5.**

**Additional file 6.**

**Additional file 7.**

## Data Availability

The CEPH genomes analyzed in this study are available with controlled access through dbGaP (phs001872.v1.p1) [68]. STRdiff is available on github (https://github.com/ScottWatkins/STRdiff.jl) [69] and zenodo (MIT release license; https://doi.org/10.5281/zenodo.7320057) [70]. Information about mutated STR loci, including parental genotypes, the de novo allele, and the motif can be found in Additional file 6: Table S9.
