# Supplementary material for: The mutational dynamics of short tandem repeats in large, multigenerational families
Source: Genome Biol. 2022 Dec 12;23:253. doi: 10.1186/s13059-022-02818-4 (PMC9743774; doi:10.1186/s13059-022-02818-4)
Supplement: Supplementary file 1 — Additional file 1. [file 13059_2022_2818_MOESM1_ESM.pdf]

Table S1. Number of STRs that were removed by specific filters. Mononucleotide repeats have been separate from other motif lengths.

	No children	Only 1	Shared with other parent	Missing data in other parent
Mononucleotide	605	171	448	73
Di-Hexanucleotide	202	111	722	58
Total	807	282	1170	131

Table S2. Distribution of de novo STRs by genomic location.

	Observed	Expected	Odds Ratio (CI 95%)	P-value
**Coding exons**	2	31	0.06 (0.01 - 0.25)	< 1e-6*
**5'-UTR**	31	36	0.86 (0.51 - 1.43)	> 0.623
**3'-UTR**	15	17	0.88 (0.41 - 1.88)	> 0.860
**Introns**	1233	1119	1.18 (1.06 - 1.32)	< 0.002*
**Intergenic**	1466	1544	0.89 (0.80 - 0.99)	> 0.037

(* significant with Bonferroni correction; Fisher's exact test)

Table S3. Distribution of *de novo* STRs in transposable elements.

	Observed	Expected	Odds Ratio (CI 95%)	P-value
**Alu**	813	266	3.92 (3.35-4.58)	< 2.2e-16*
**LINE-1**	167	445	0.33 (0.28-0.40)	<2.2e-16*
**SVA**	5	4	1.25 (0.27-6.31)	1

(*significant with Bonferroni correction; Fisher's exact test)

Table S8. Read-based estimates of STRdiff accuracy using IGV.

Index	Ped	Id	Position (hg19)	Predicted transmission	IGV transmission	Sex of transmitter	Predicted change	IGV change	Pred. sex	Pred. size
1	1375	8819	20:46109558	8323	8323	male	+4	+4	OK	OK
2	1440	1292	21:21634951	1293	1293	female	+6	+3	OK	fail
3	1347	8445	10:68161277	8369	8369	female	-4	-4	OK	OK
4	1459	2024	1:61917927	2034	2034	female	-3	-3	OK	OK
5	1421	1310	9:112241290	1314	1314	female	+1	+1	OK	OK
6	1463	2188	8:65373478	2213	2213	female	-4	-4	OK	OK
7	1463	2209	9:91199686	2281	2281	male	+2	+2	OK	OK
8	1463	2188	9:107770736	2213	2213	female	+4	+4	OK	OK
9	1347	8445	2:240119544	8368	8368	male	+2	+2	OK	OK
10	1347	8445	9:90190129	8368	8368	male	+2	+2	OK	OK
11	1345	8333	12:117577179	8326	8326	female	-5	-5	OK	OK
12	1345	8333	12:9855972	8326	8326	female	+4	+4	OK	OK
13	1345	8333	22:46973294	8326	8326	female	-	+3	OK	-
14	1345	8334	13:23249475	8329	8329	male	+2	+2	OK	OK
15	1345	8334	21:28649979	8329	8329	male	±2	+2	OK	OK
16	1346	8365	6:144453196	8366	8366	female	+3	+3	OK	OK
17	1346	8365	20:16307714	8367	8367	male	+2	+2	OK	OK
18	1346	8365	10:24297088	8367	8366	female	-	+8	fail	fail
19	1350	8410	7:136457485	8403	8403	female	+5	+5	OK	OK
20	1347	8440	17:74646299	8413	8413	female	-5	-5	OK	OK
21	1347	8440	8:9262507	8413	8413	female	+2	+2	OK	OK
22	1347	8440	19:40796156	8414	8414	male	-4	-4	OK	OK
23	1350	8404	6:104178218	8431	8431	male	-12	-12	OK	OK
24	1353	8463	11:41701236	8649	8649	female	-	-4	OK	OK

Figure S1. *De novo* STRs that intersect transposable elements. *Alu* elements intersect approximately 30% of the *de novo* STRs that were identified in this study (red). LINE-1 (L1) insertions show a smaller proportion (green), with those that intersect SVA insertions shown in the small purple sliver. The remaining portion of the chart (blue) shows the *de novo* STRs that intersect non-transposable element (TE) regions of the genome.

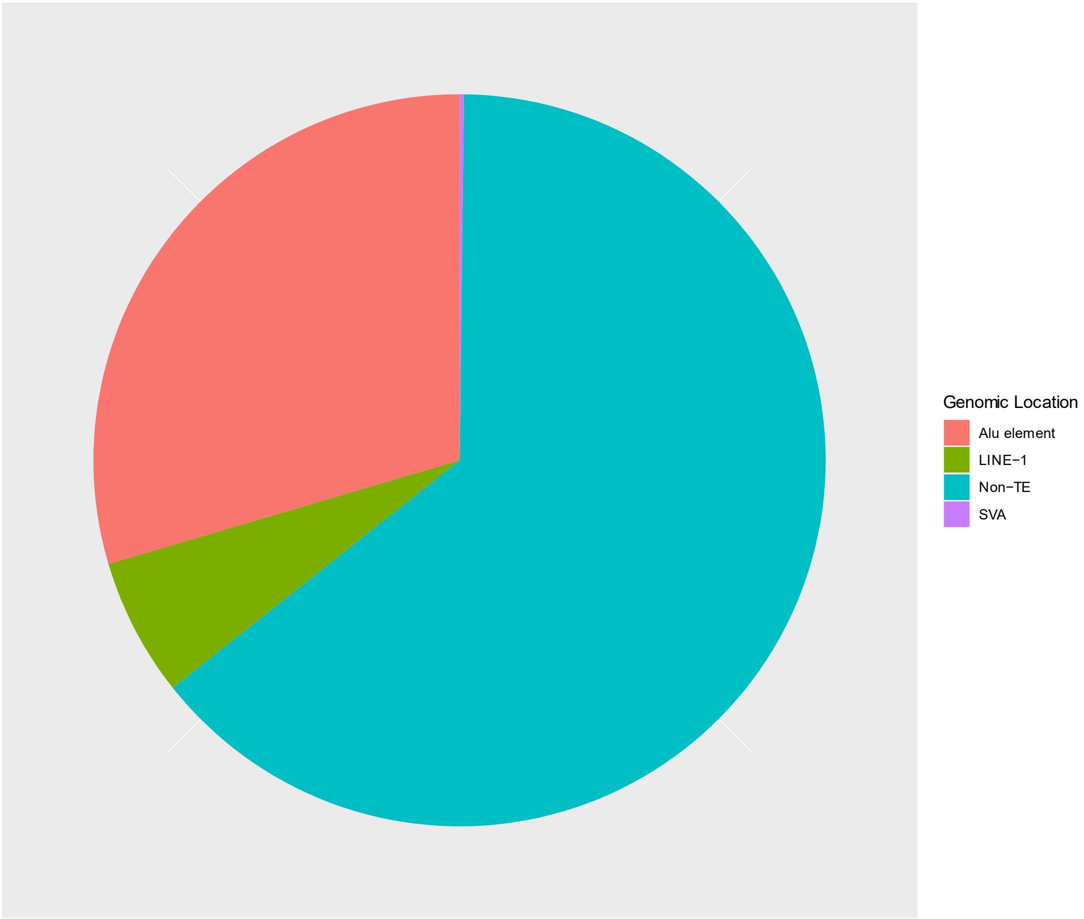

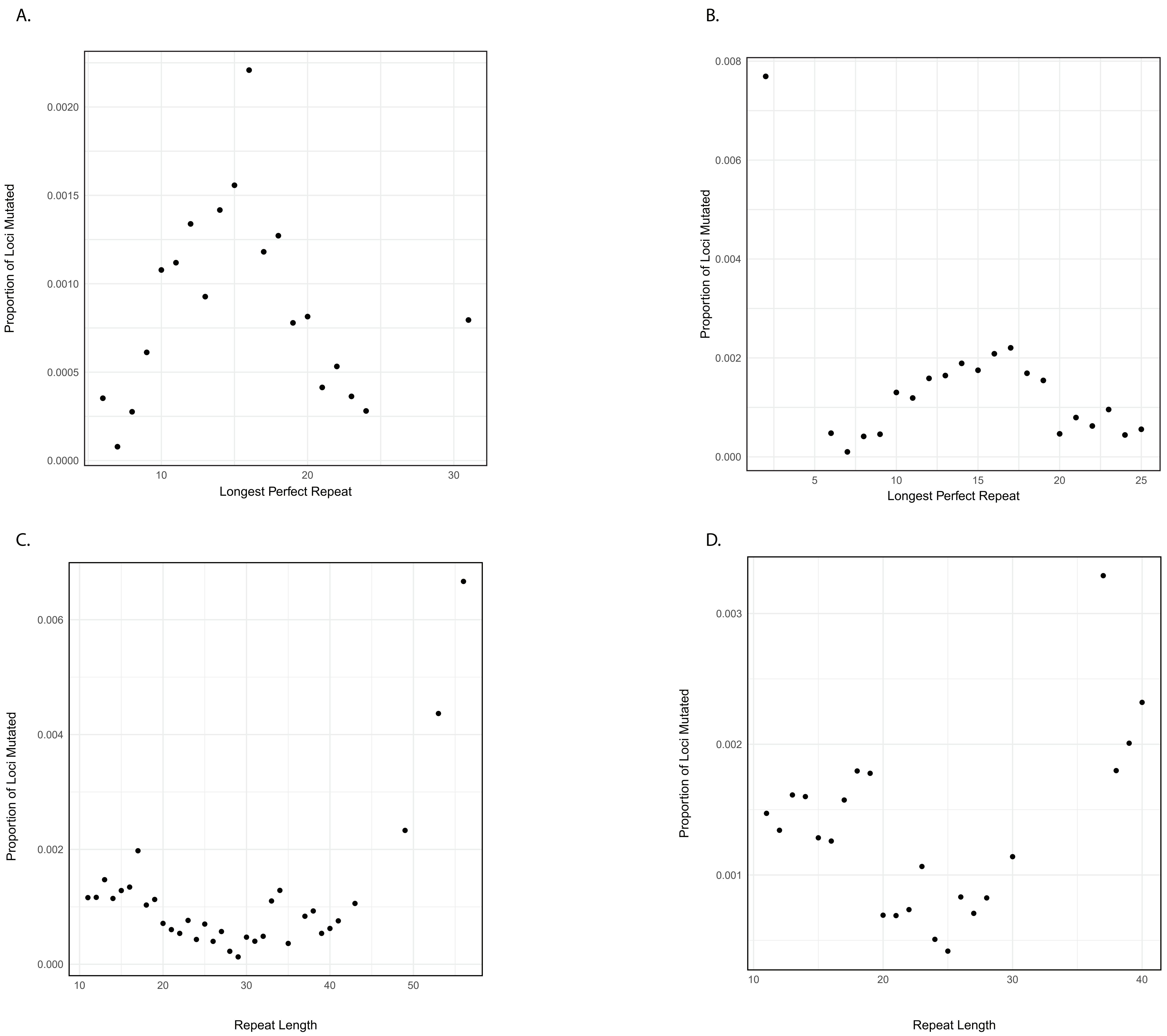

Figure S2. Relationship between the longest perfectly repeating segment of mononucleotide repeats and mutability. A) Mutability of mononucleotide loci within *Alu* elements as the longest perfectly repeating segment increases. B) Mutability of mononcleotide repeats in non-*Alu* regions of the genome as the longest perfectly repeating segment increases. C) Repeat length and mutability for mononucleotide repeats within *Alu* elements. D) Repeat length and mutability for mononucleotide repeats in non-*Alu* regions of the genome.

Figure S3. Updated trendlines for mononucleotide or dinucleotide repeats. A. A better fitting trendline for the mutation rate of mononucleotide repeats as repeat length increases. B. A better fitting trendline for the mutation rate of mononucleotide repeats as the length of the longest perfect stretch increases. C. A better fitting trendline for the mutation rate of dinucleotide repeats as the length of the longest perfect stretch increases.

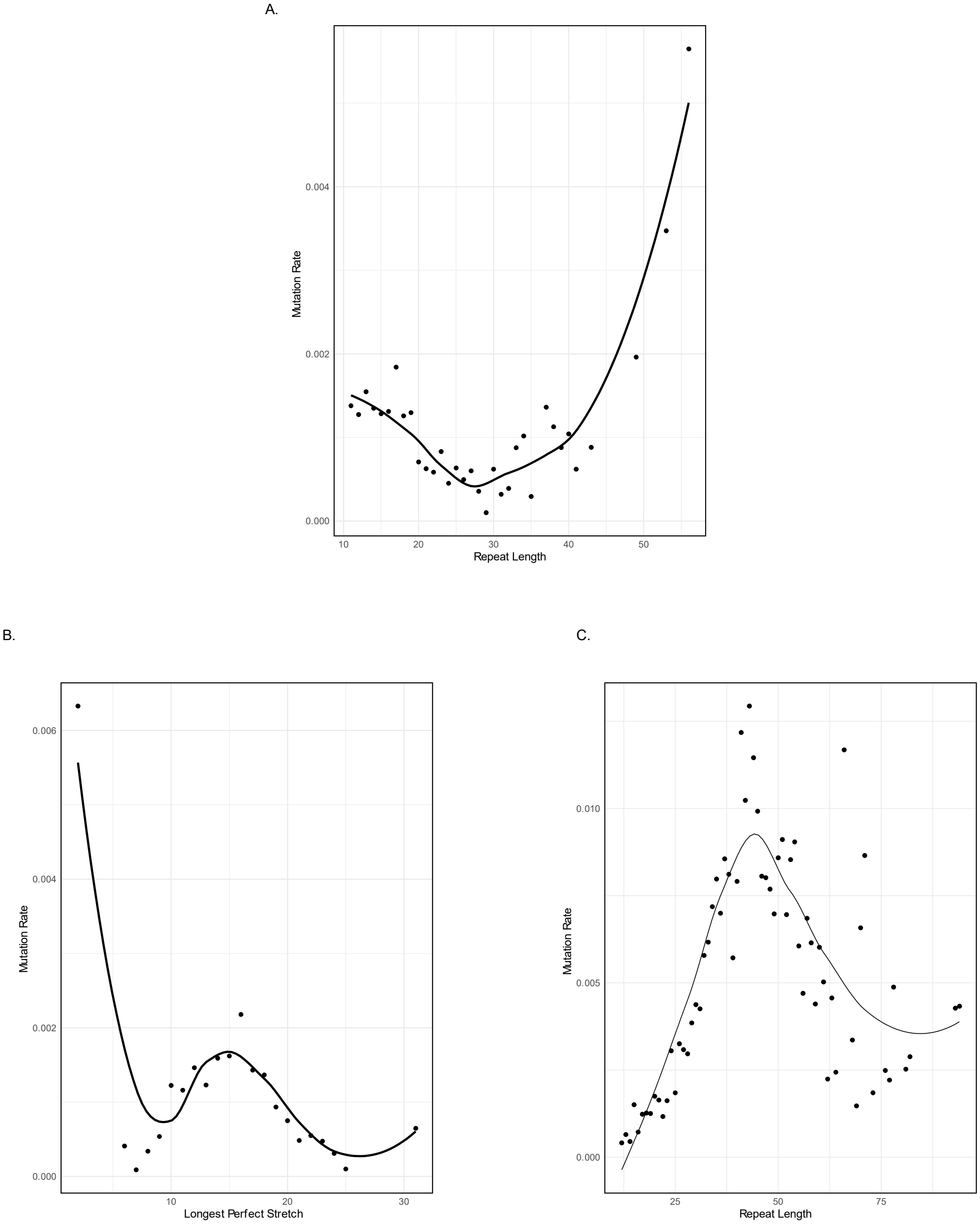

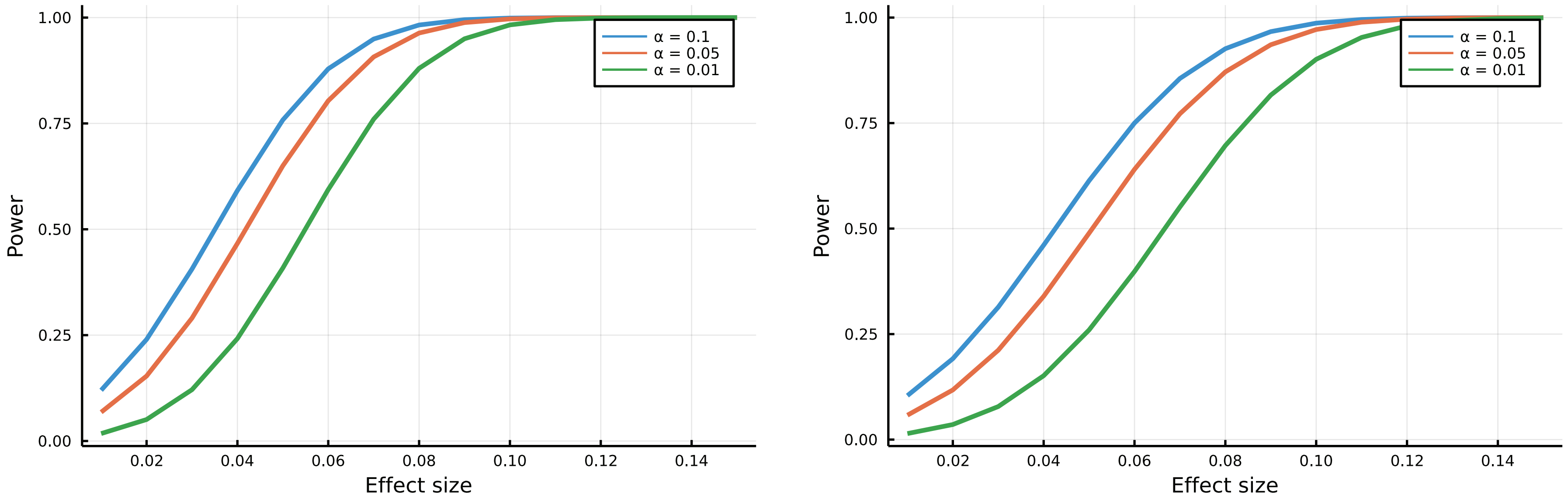

Figure S4. Power analyses of sex transmission bias. A) Power analysis including all *de novo* STRs identified in this study. B) Power analysis excluding mononucleotide repeats.

Figure S5. Stepwise changes observed all *de novo* STR alleles follow an exponential distribution. All motif lengths except for trinucleotide repeats fall within the 95 percent confidence interval. Trinucleotide repeats fall within the 90 percent confidence interval.

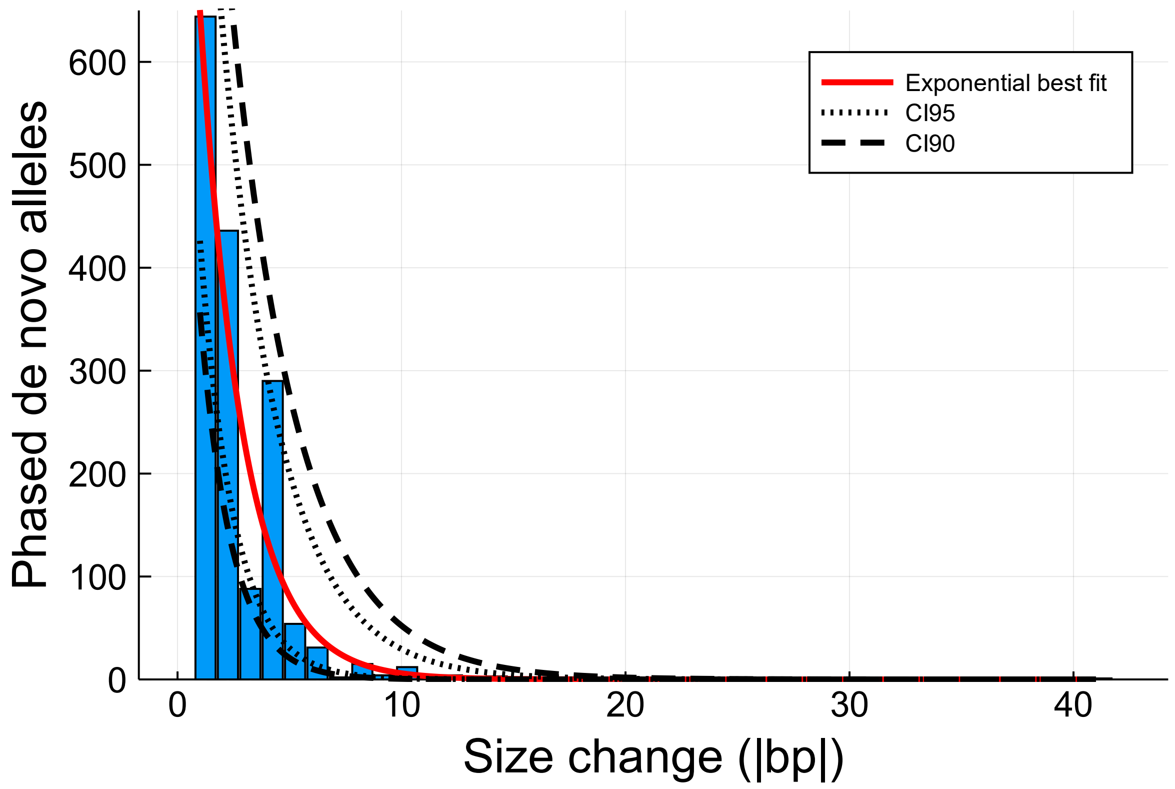

Figure S6. Comparison of STRdiff results to IGV images.

De novo 1

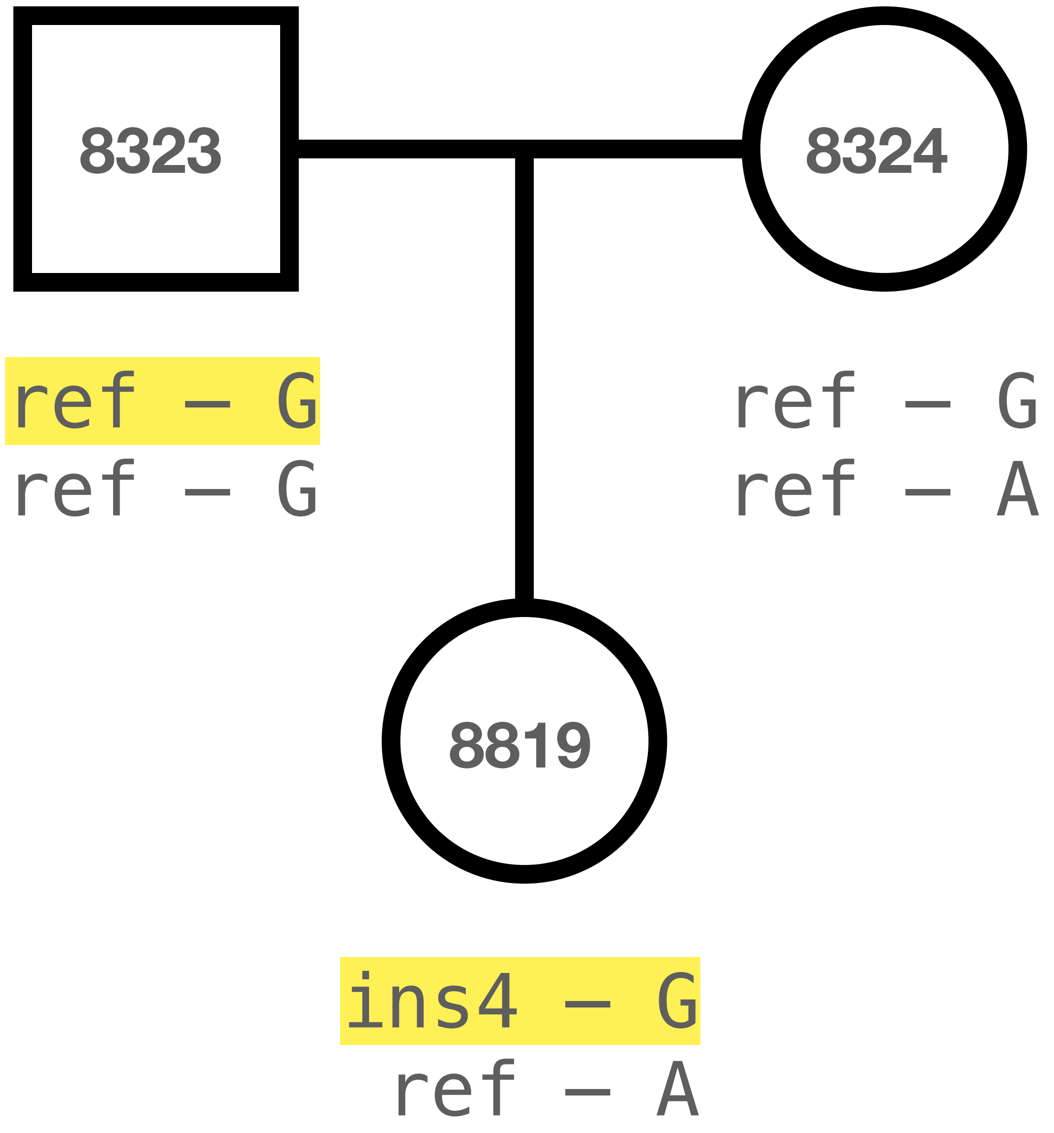

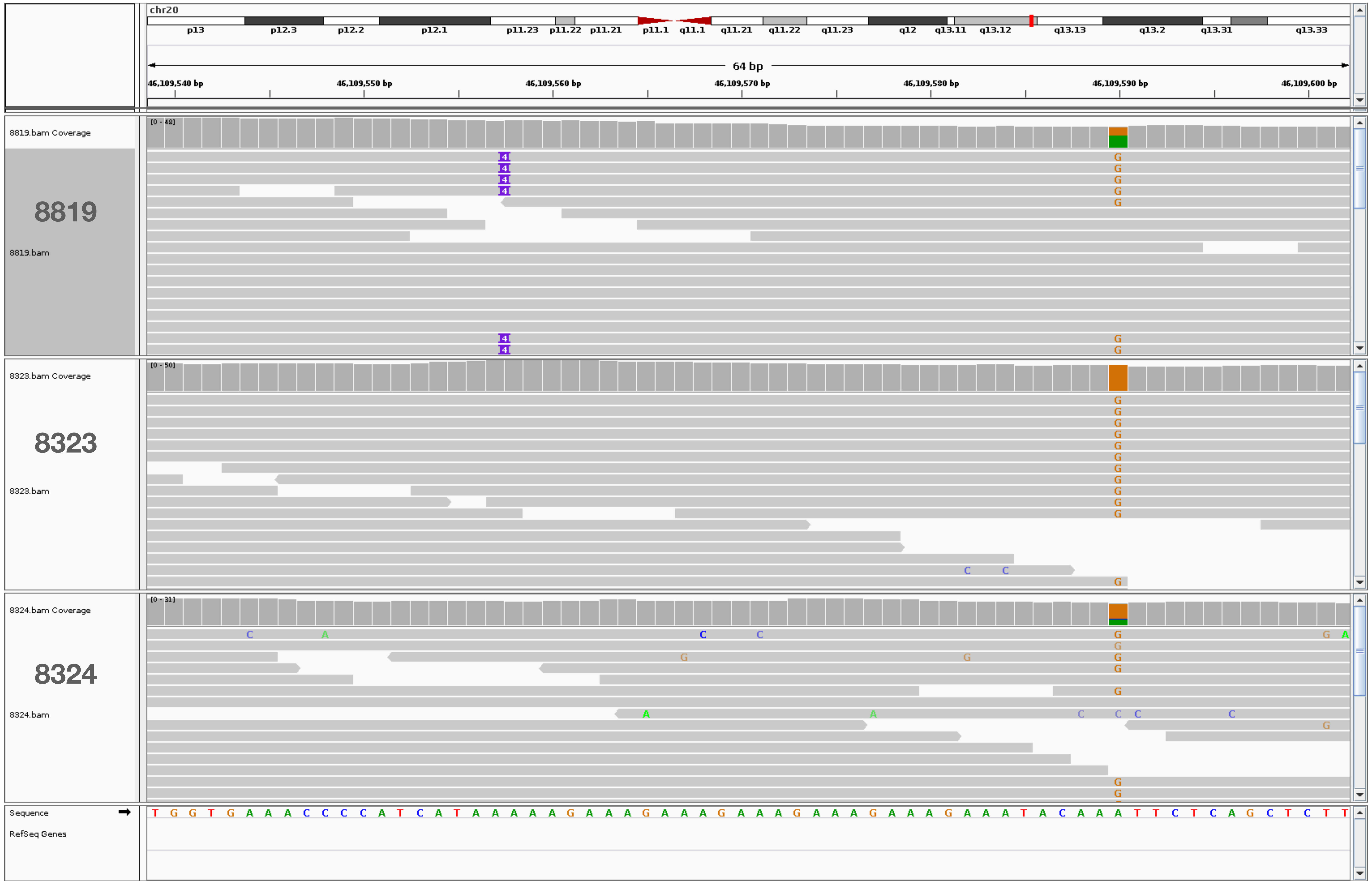

STRdiff header: **ped iid loc trgp trgp_sex trgp_str trgp_size p_strp_size str_change str_change_bp numoffspr gp_probs uss hapsize str_type**

8819_8820 8819 20:46109558 8323q male 0 27 1 31 1 4 3 [0.524038, 0.966346, 0.552885, 0.596154] 37 190000 4

De novo 2

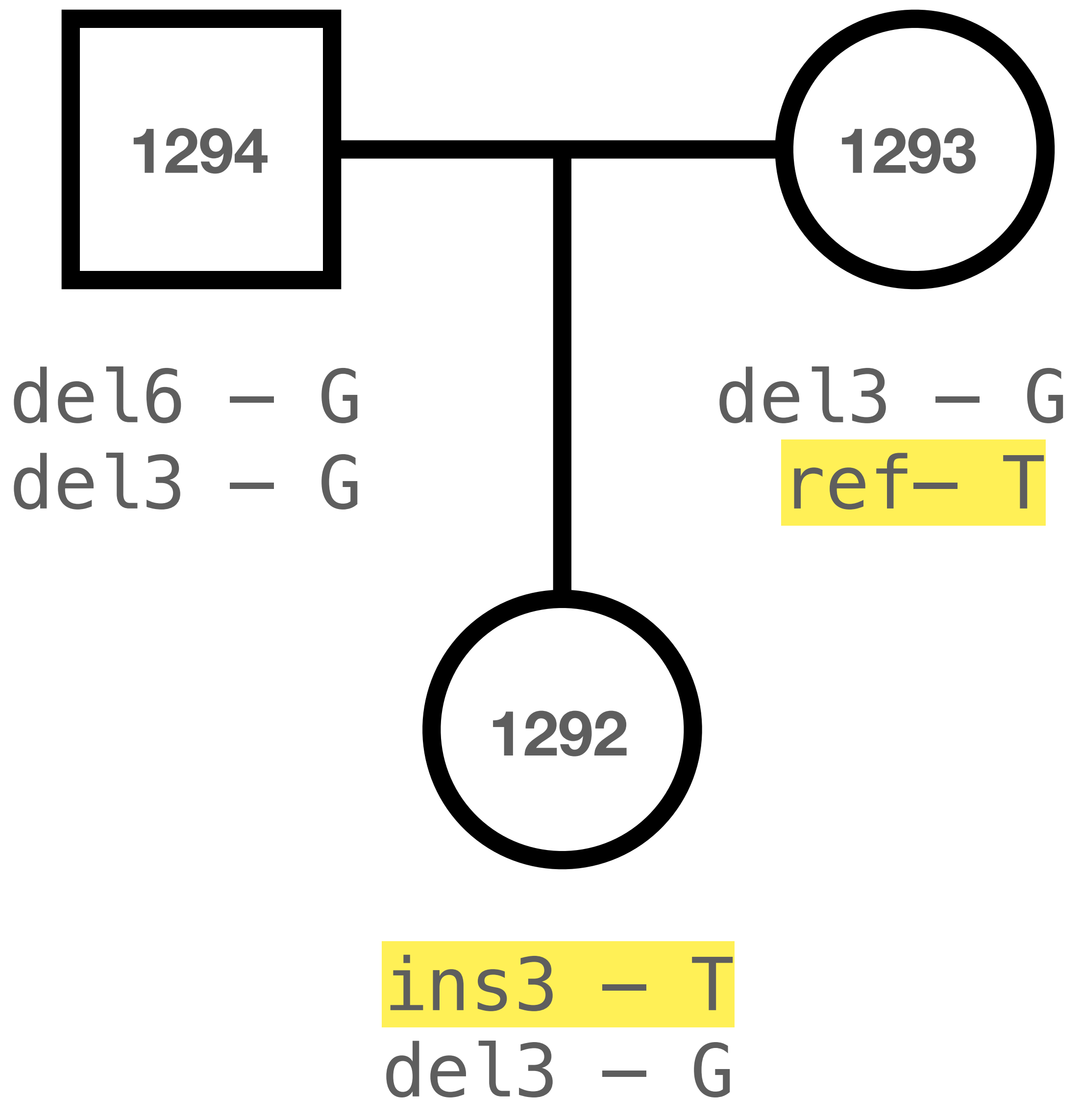

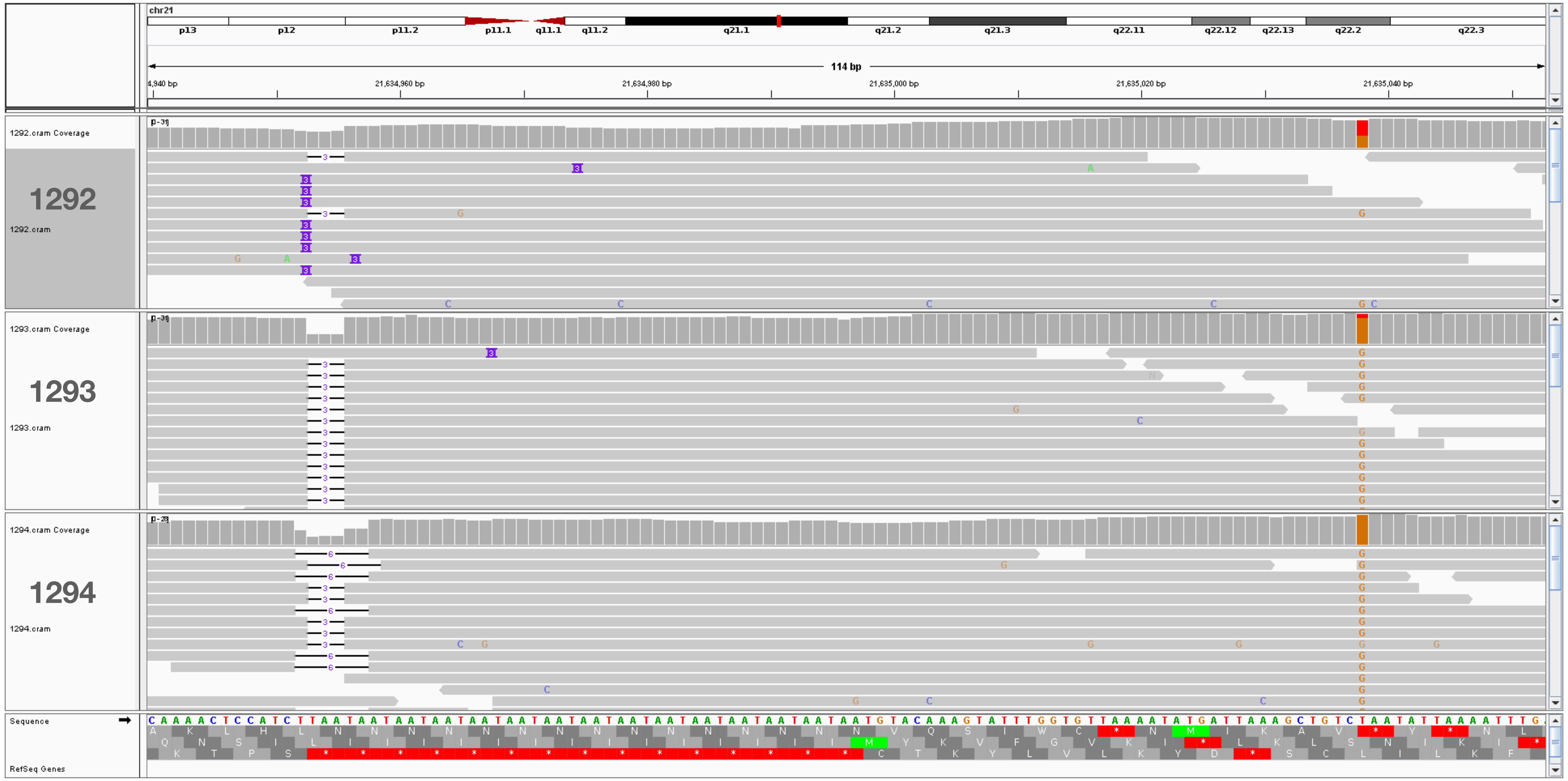

del3-G must be transmitted from male gp

1291_1292 1292 21:21634951 1293p female 4 45 5 51 1 6 3 [0.376344, 0.327957, 0.994624, 0.397849] 60 50000 3

T allele in mother is weak, 6-bp repeat was expected?

De novo 3

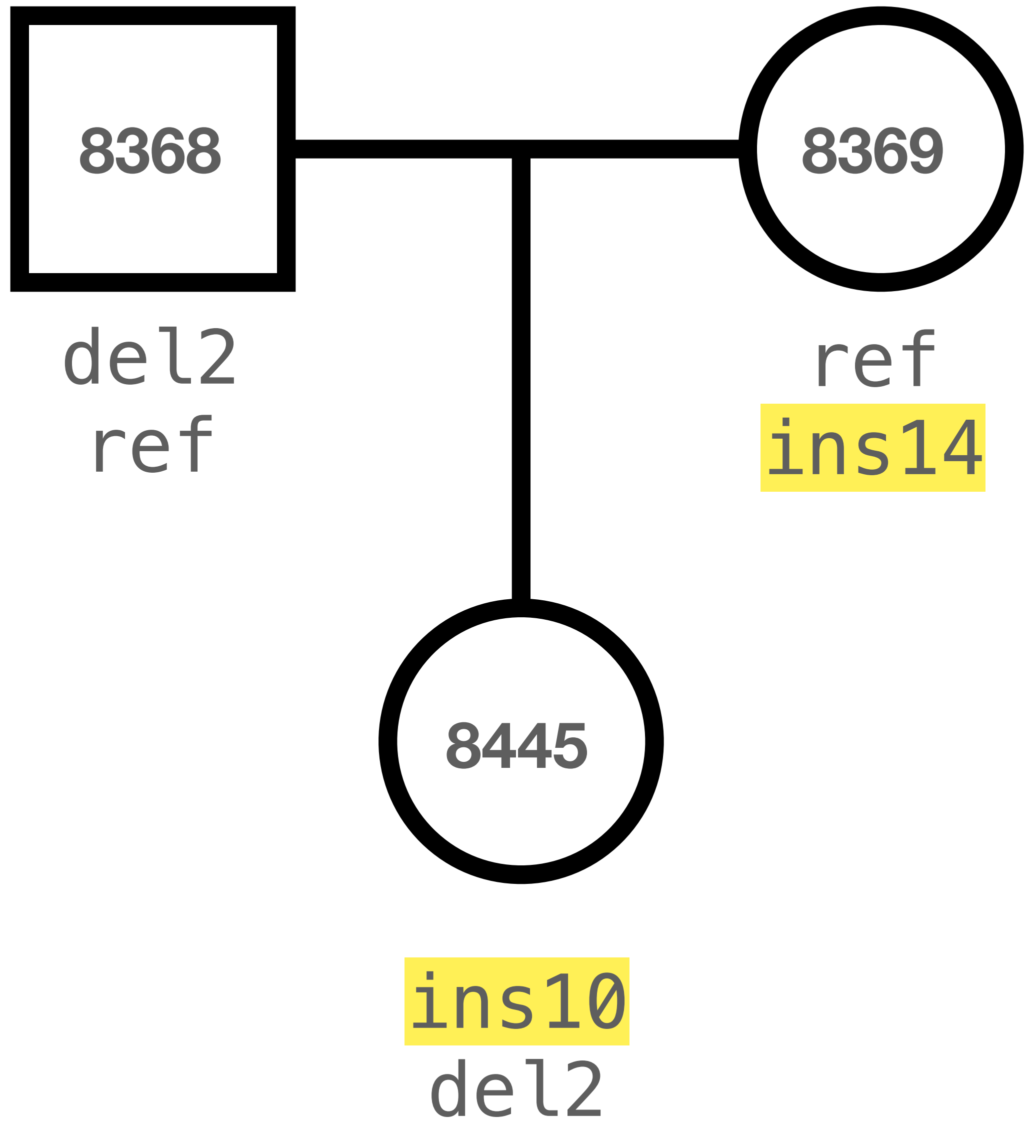

No flanking snp but father must transmit del2 allele

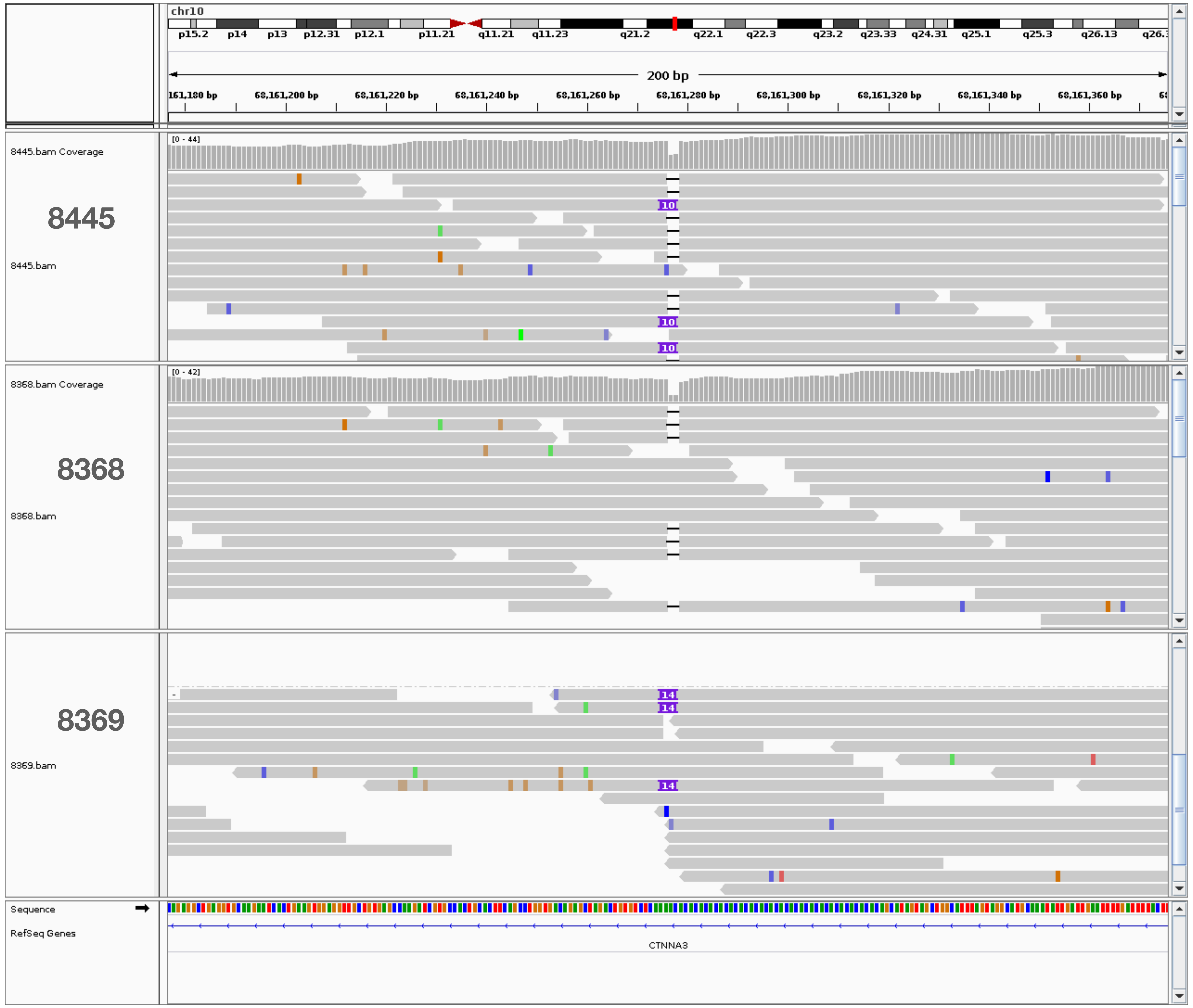

10 | 11 reads
-2 | 15 reads
-4 | 1 read
-6 | 1 read

1347 8445 10:68161277 8369p female 7 57 6 53 -1 -4 1 [0.115789, 0.115789, 0.989474, 0.242105] 75 30000 2

De novo 4

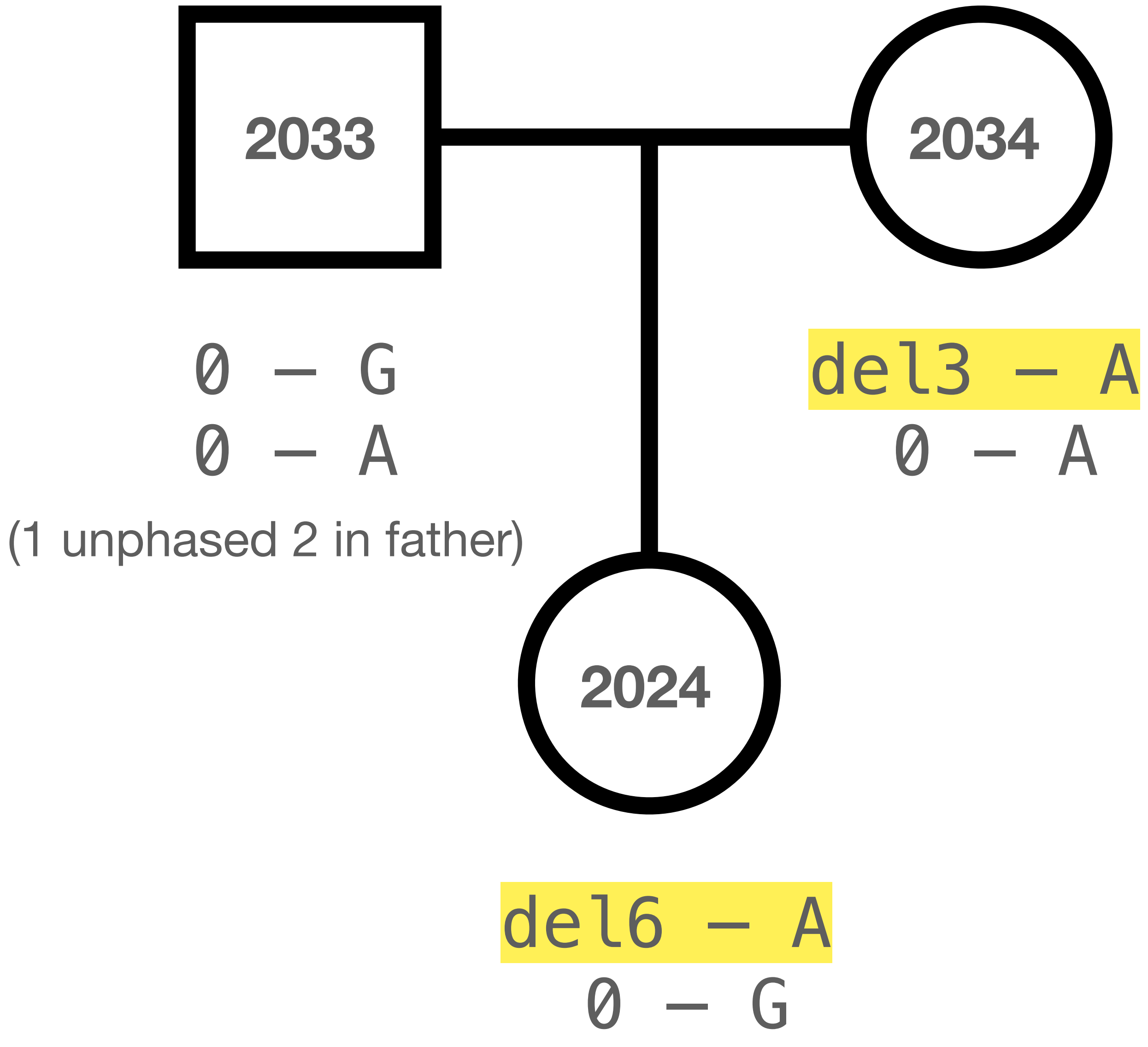

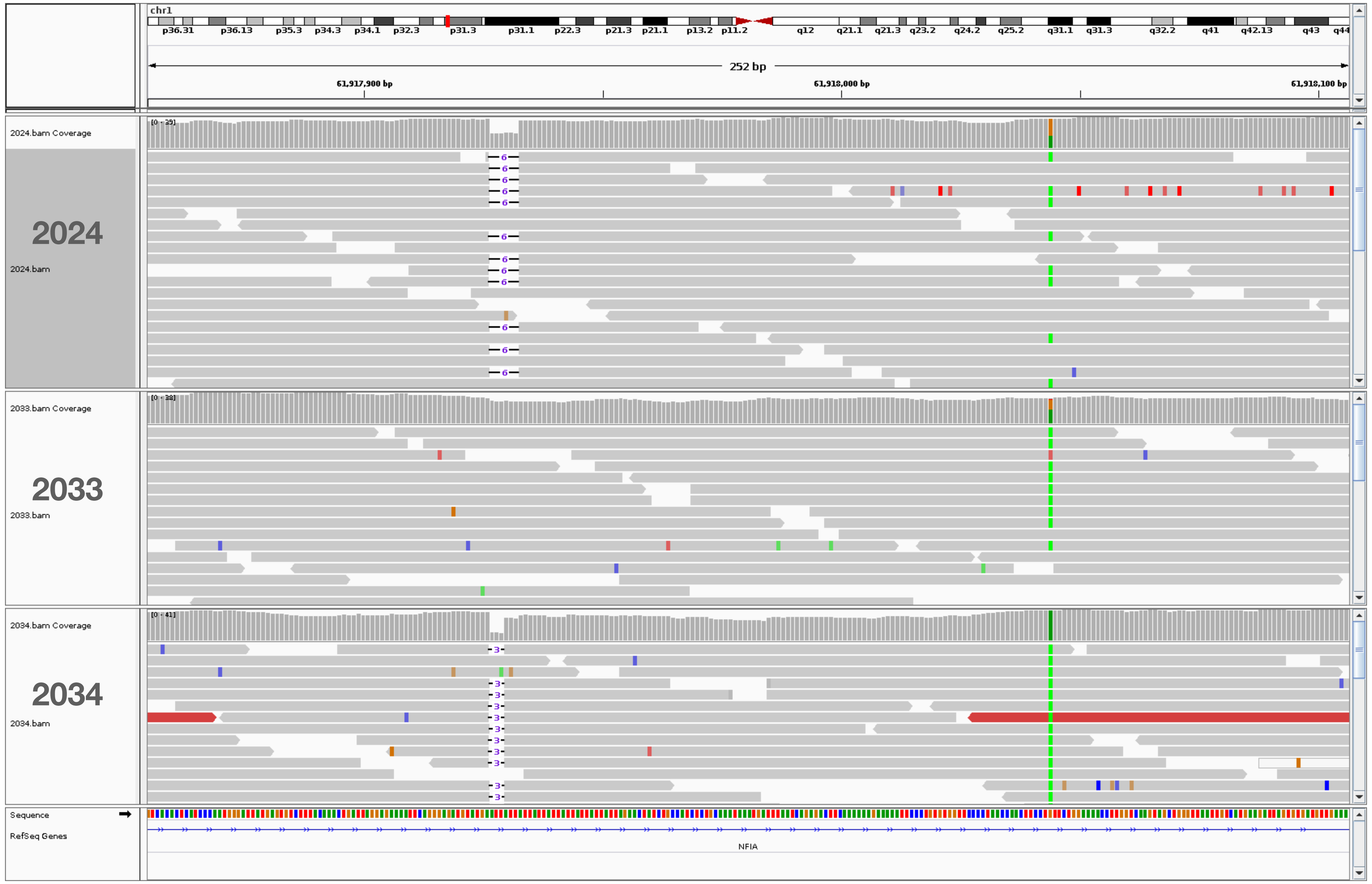

2023_2024 2024 1:61917927 2034p female 2 25 1 22 -1 -3 2 [0.583333, 0.416667, 0.958333, 0.458333] 38 20000 3

De novo 5

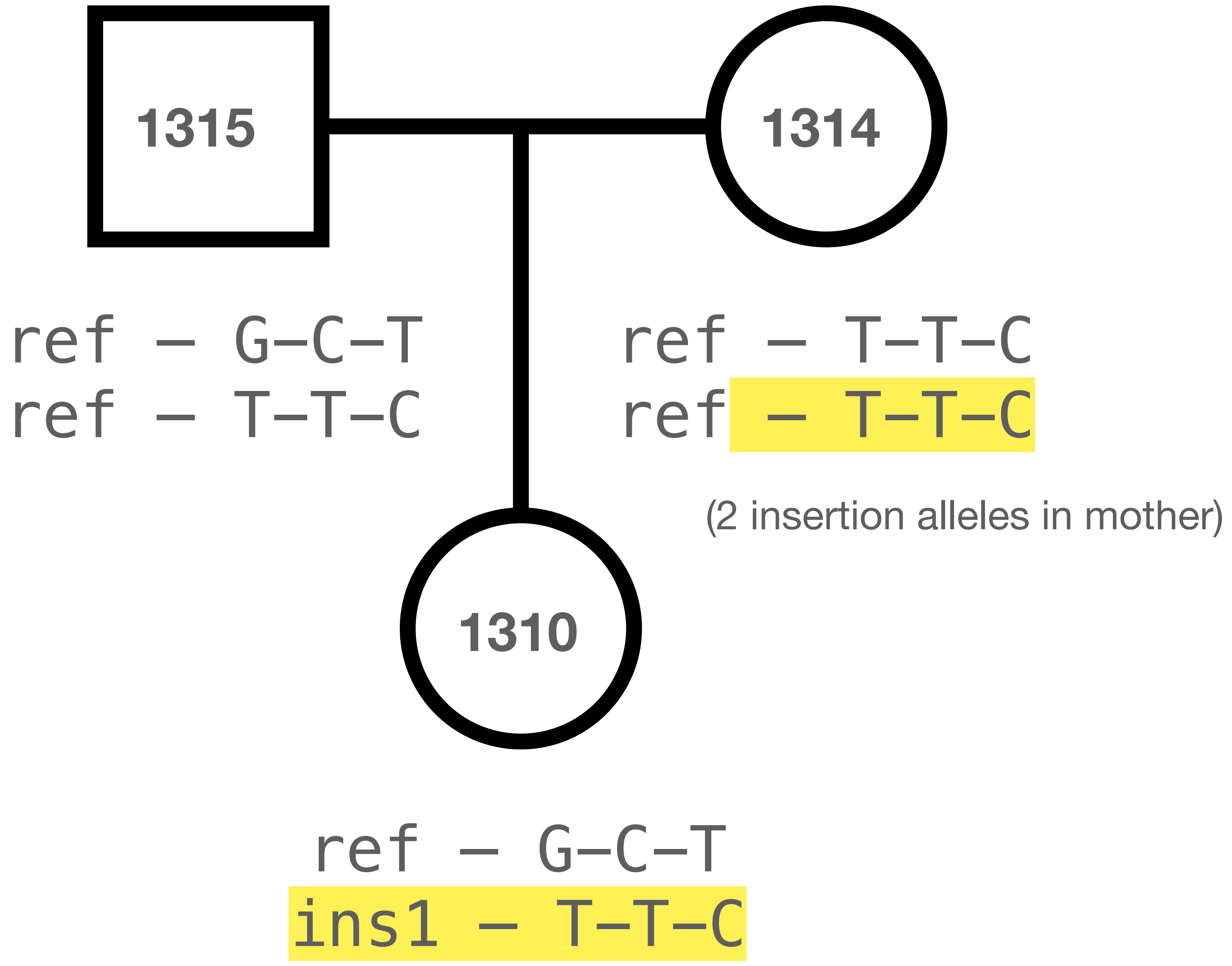

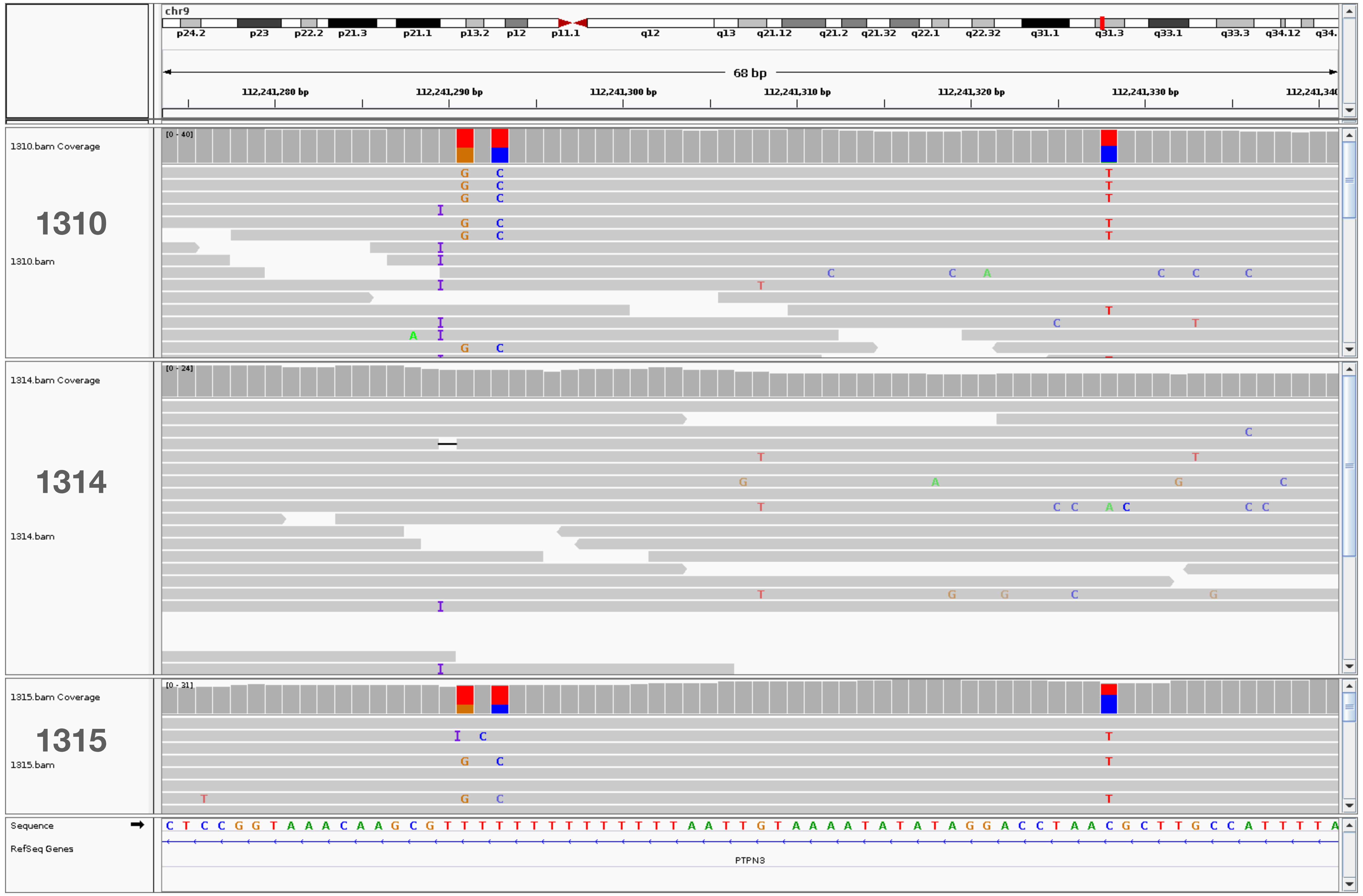

(ins1 is seen in transmitting female at a very low frequency)

1421 1310 9:112241290 1314p female 0 14 2 15 2 1 2 [0.641264, 0.689591, 0.806691, 0.605948] 12 260000 1

De novo 6

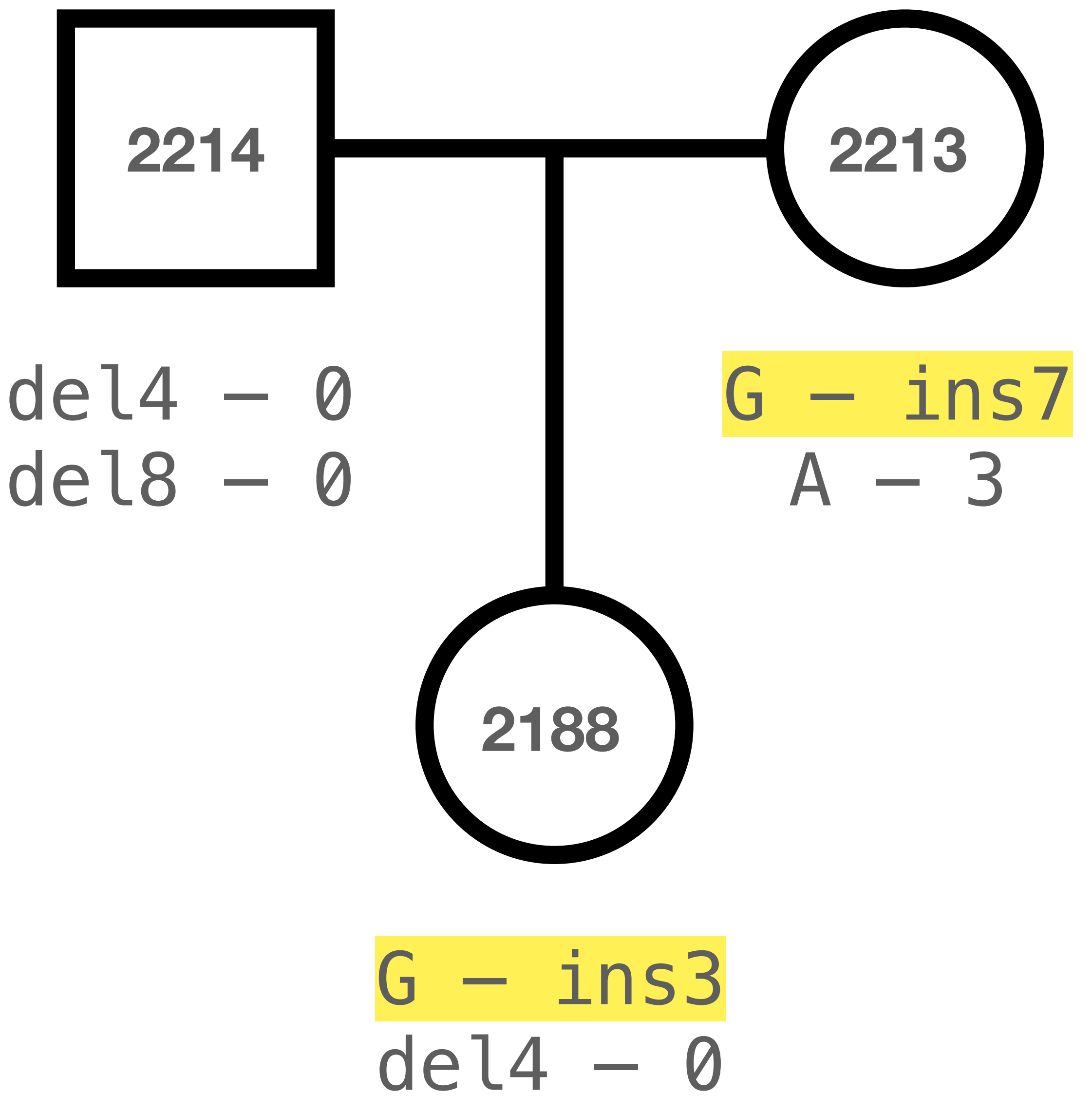

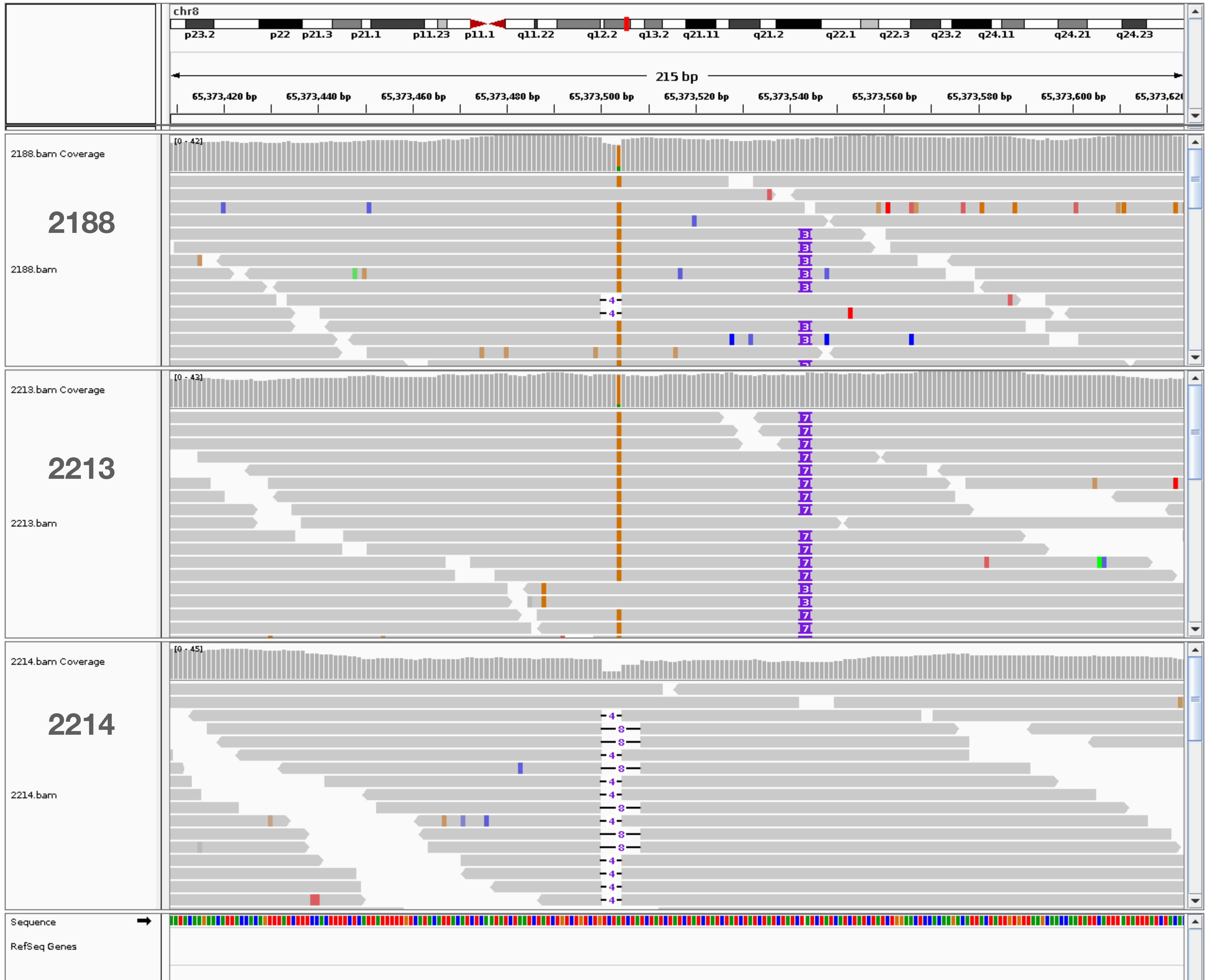

Mutation appears offset from predicted location but within read length

1463 2188 8:65373478 2213p female 4 92 3 88 -1 -4 2 [0.605839, 0.580292, 0.919708, 0.740876] 18 2900004

De novo 7

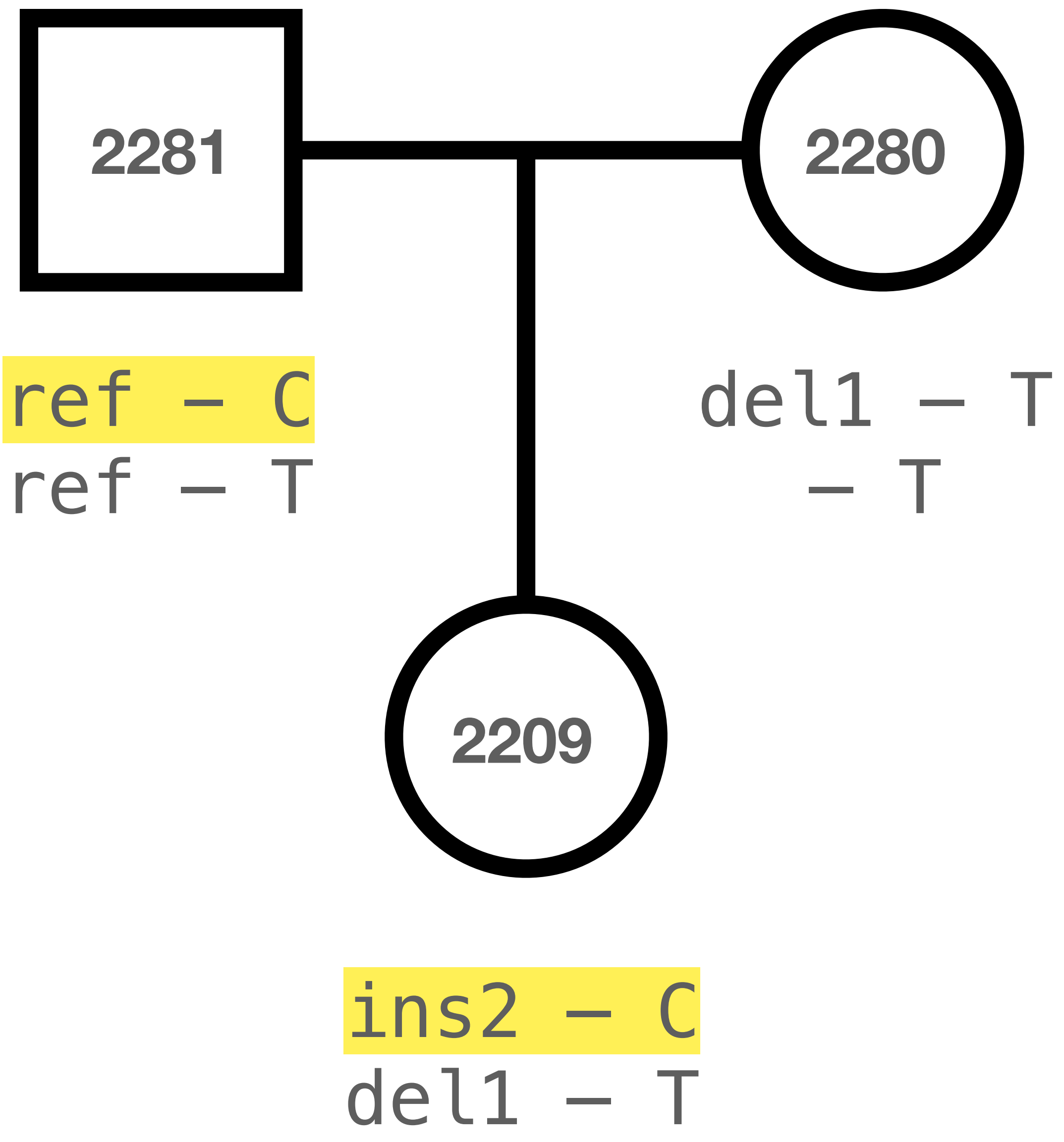

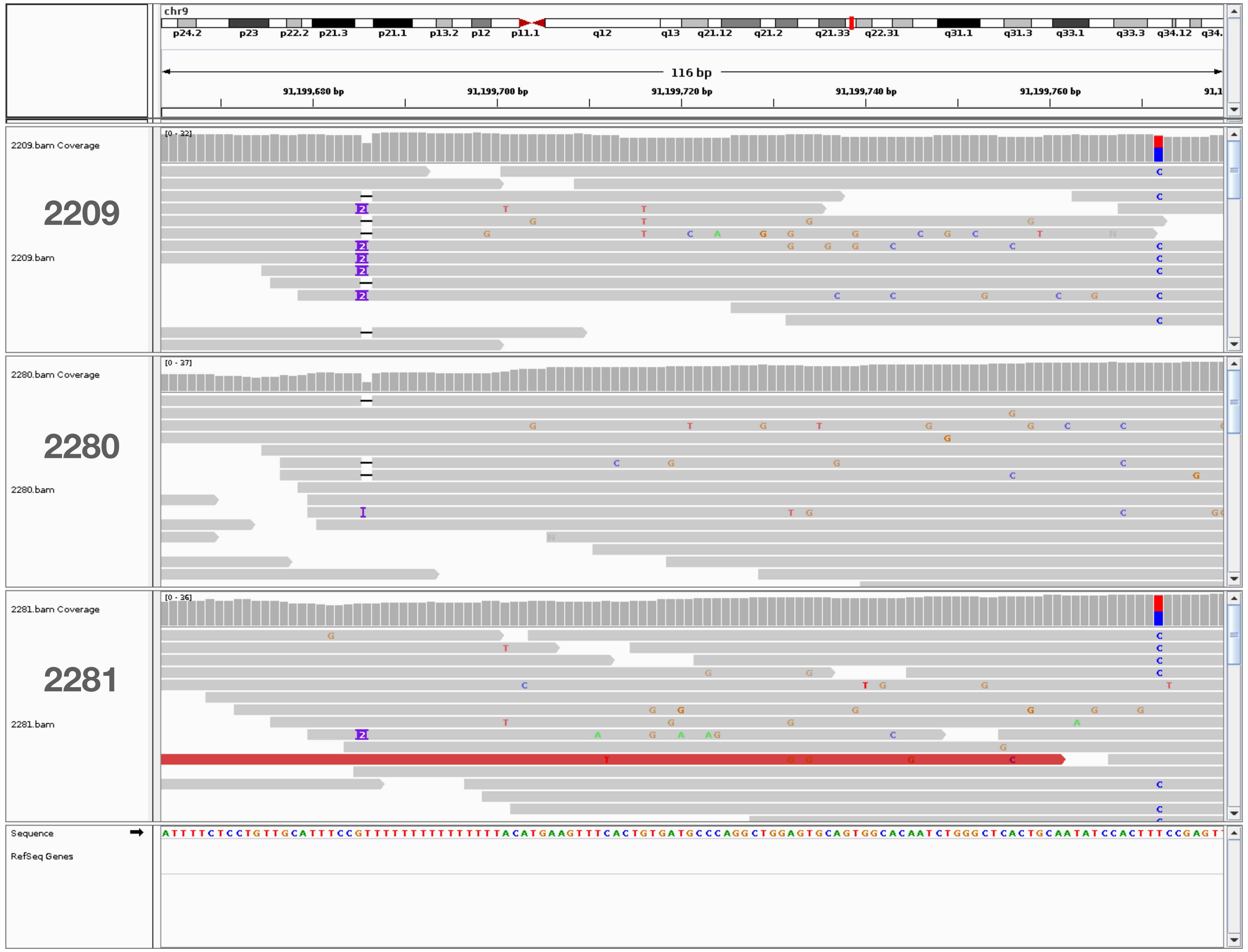

GP (mother) is T/T and must transmit del1 allele
Father is C/T; ins2 must come from father, but linked 0-C reads
One ins2 and one del2 seen in father reads
0-C most likely allele from transmitting -> ins2

1463 2209 9:91199686 2281q male 0 15 2 17 2 2 6 [0.642353, 0.969412, 0.541176, 0.463529] 33 290000 1

De novo 8

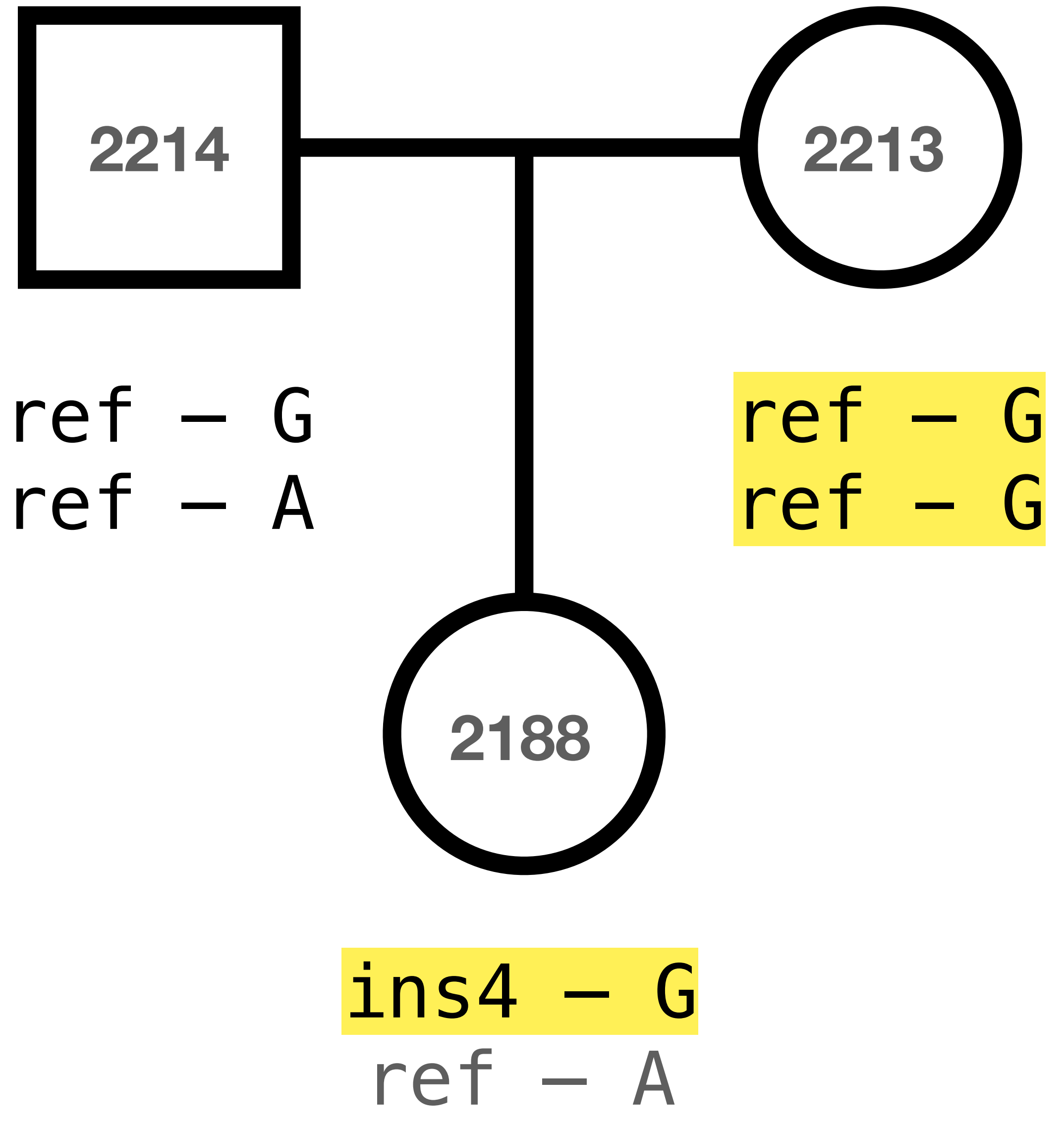

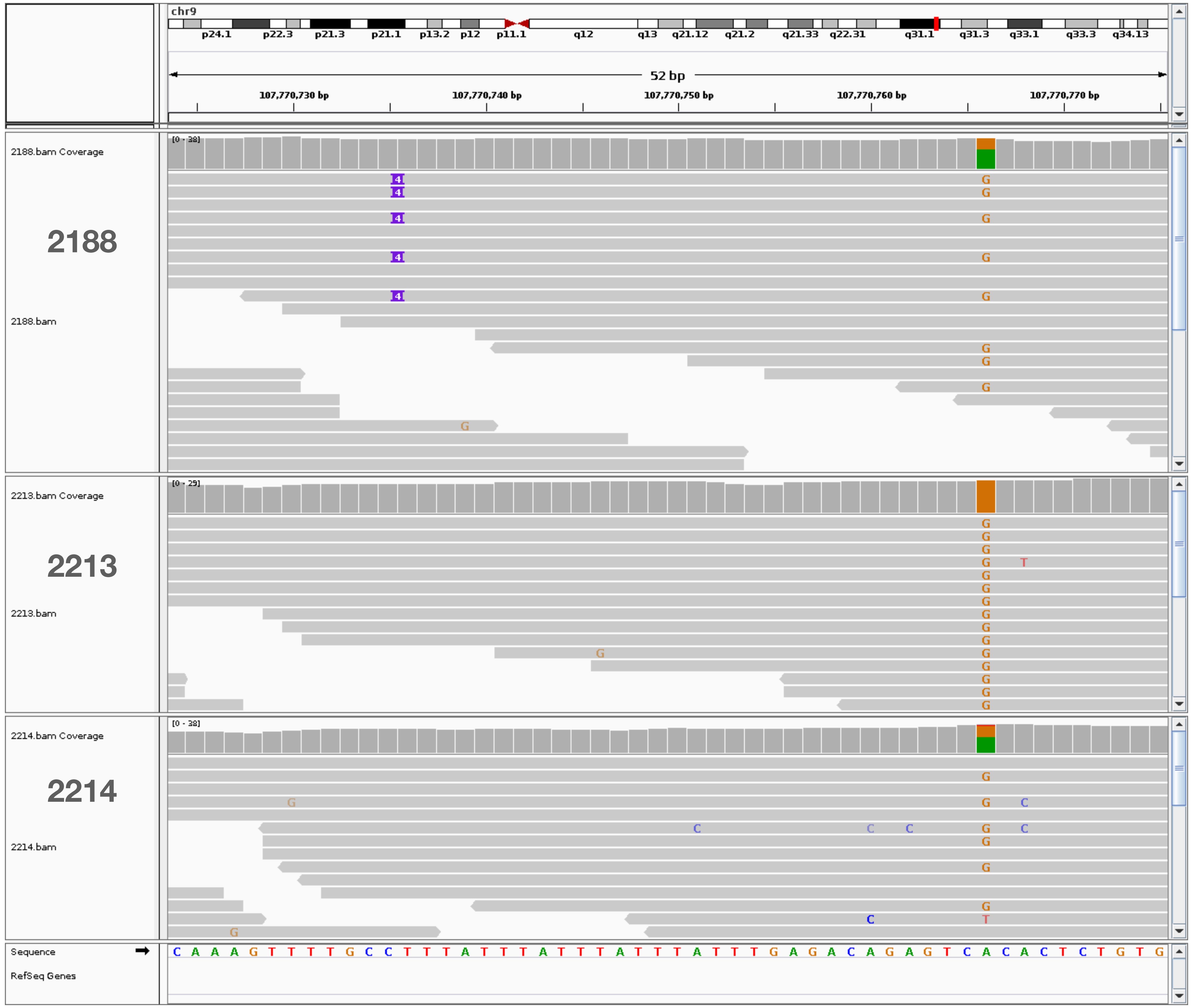

1463 2188 9:107770736 2213q female 0 19 1 23 1 4 4 [0.447368, 0.671053, 0.460526, 0.973684] 30 20000 4

De novo 9

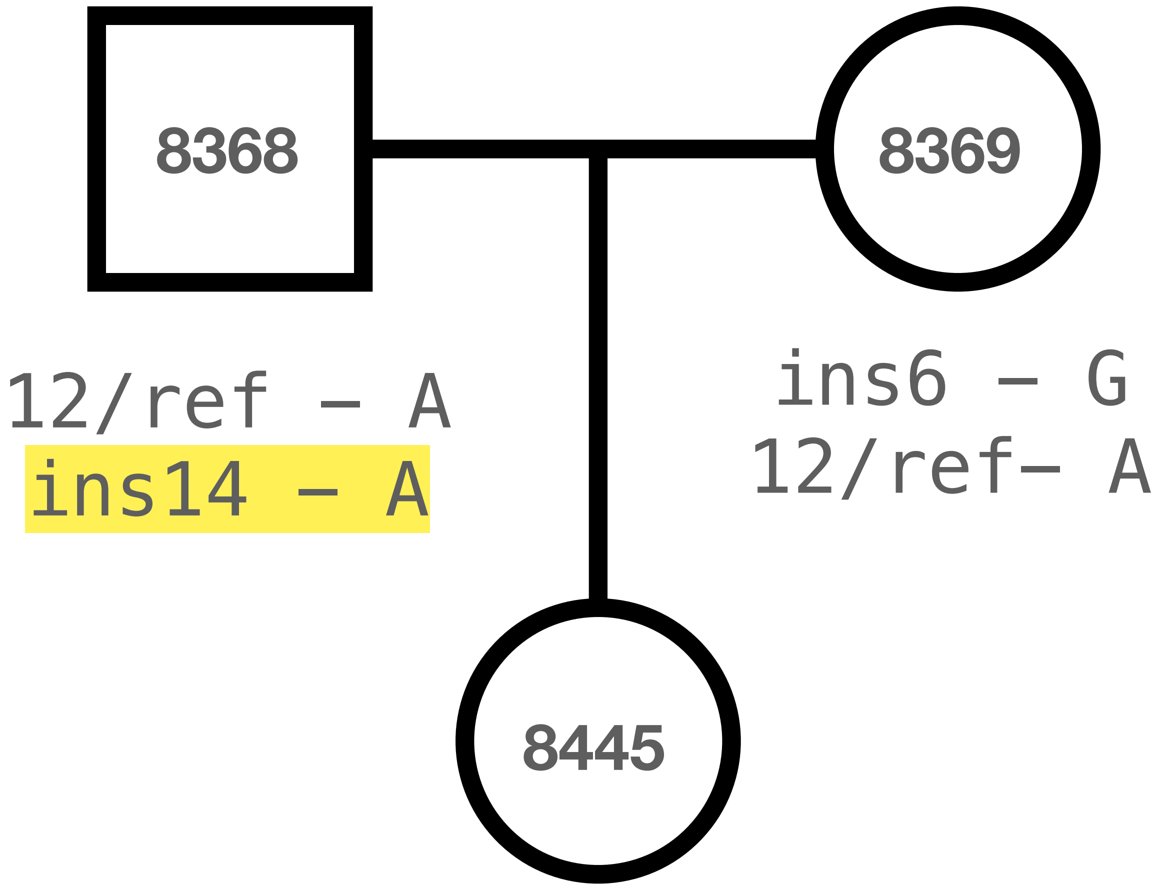

12/ref – A
ins14 – A

ins6 – G
12/ref– A

ins16 – (A)
ins6 – G

ins6-G must inherit from 8369 (mother)
Father 8368 is A/A

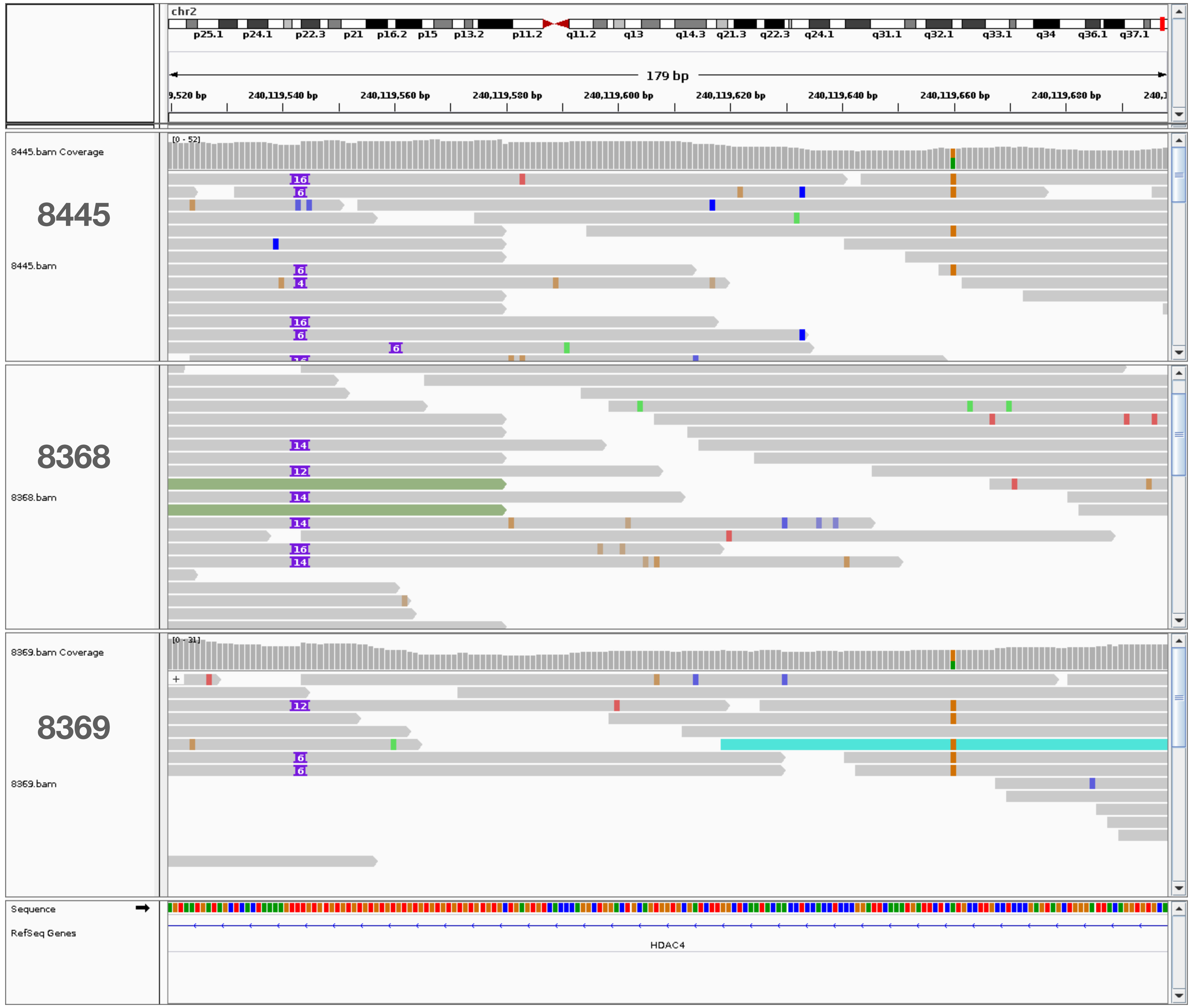

16 | 14 reads
6 | 12 reads
4 | 1 read
0| 4 reads

1347 8445 2:240119544 8368q male 5 50 6 52 1 2 6 [0.684455, 0.962877, 0.491879, 0.658933] 28 160000 2

De novo 10

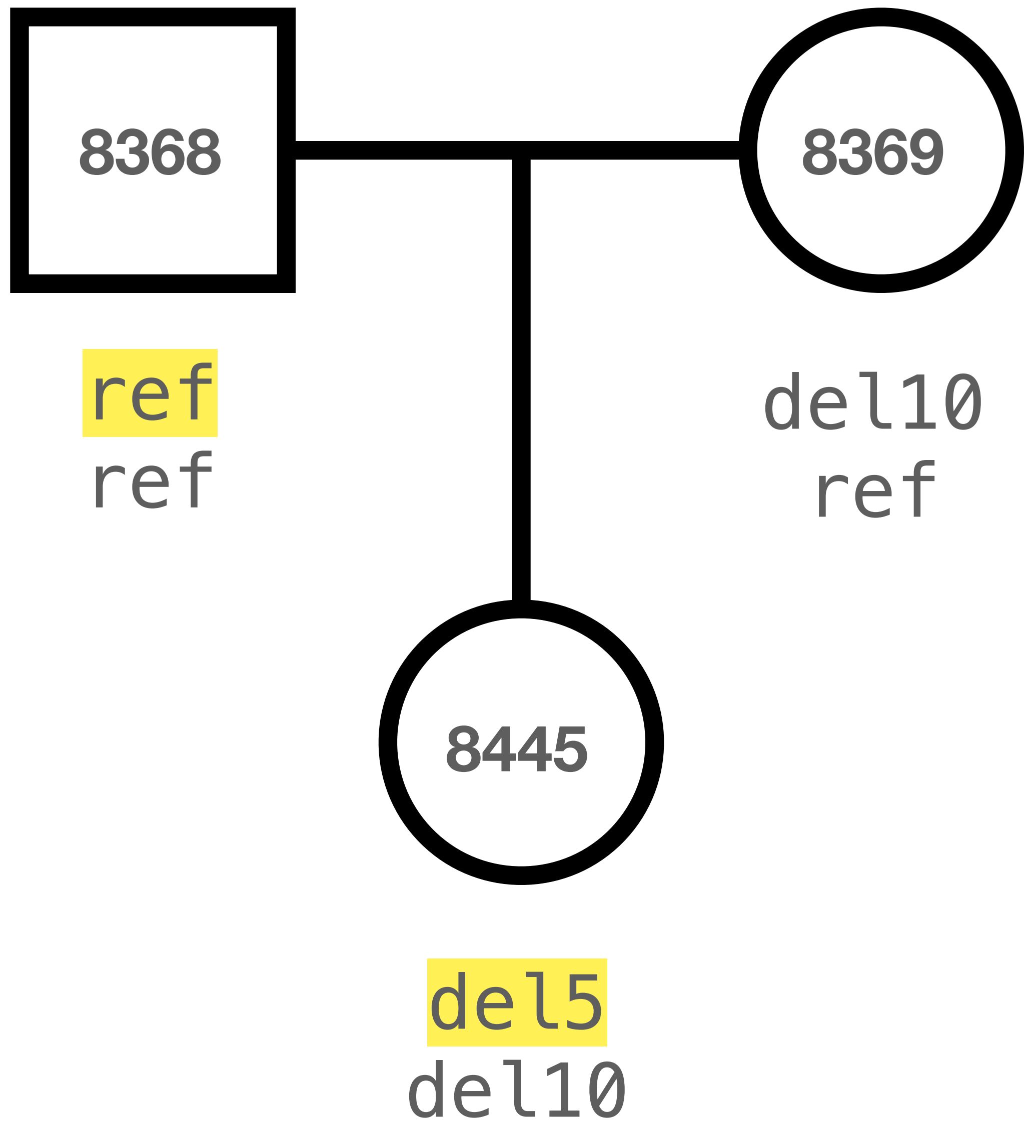

(Low freq. del5 seen in 8368)

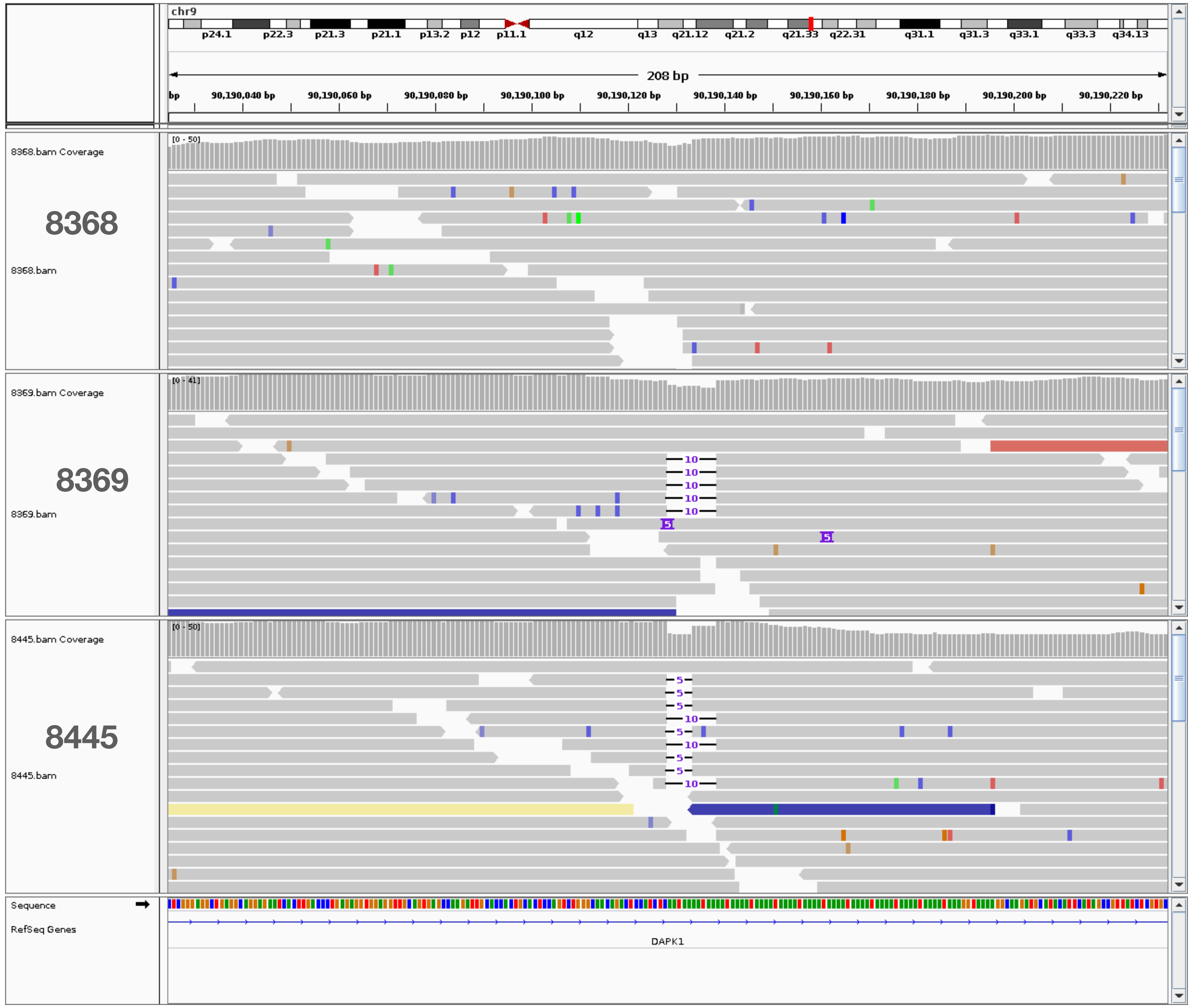

1347 8445 9:90190129 8368p male 0 61 4 56 4 -5 6 [0.953459, 0.655346, 0.413836, 0.681761] 27 2100005

De novo 11

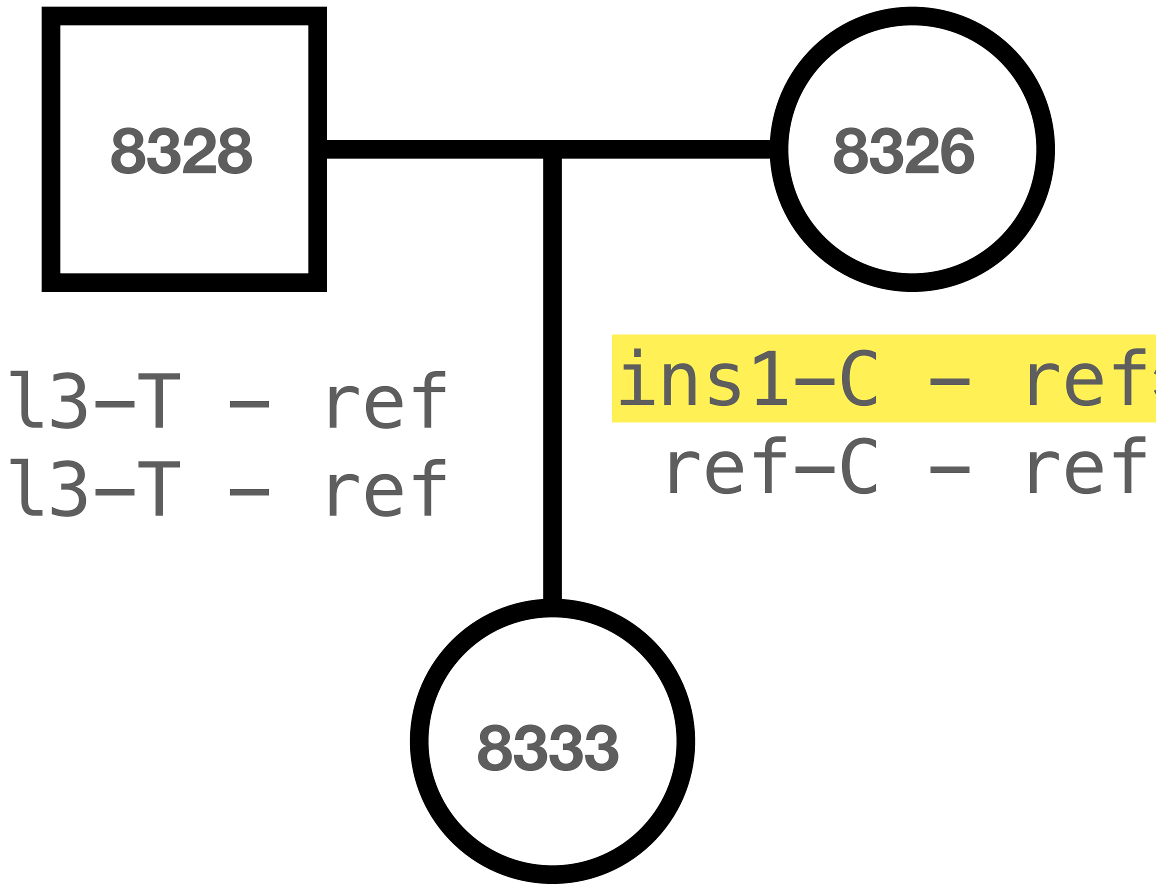

del3–T – ref
del3–T – ref

ins1–C – ref*
ref–C – ref

ins1–C – del6
T – ref

* one del6 read observed in mother

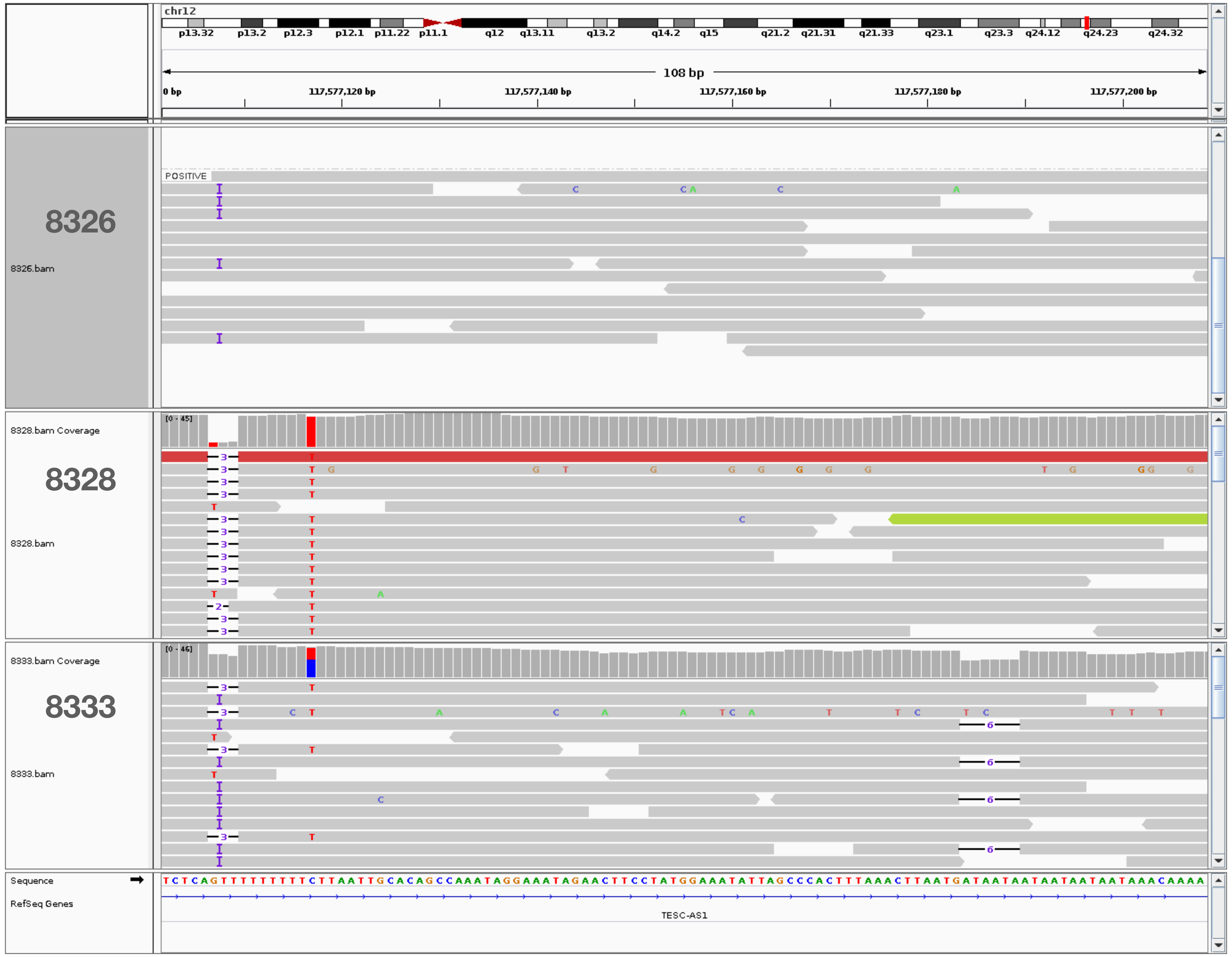

1345 8333 12:117577179 8326p female 0 24 1 18 1 -6 1 [0.373984, 0.398374, 0.95122, 0.780488] 17 90000 3

De novo 12

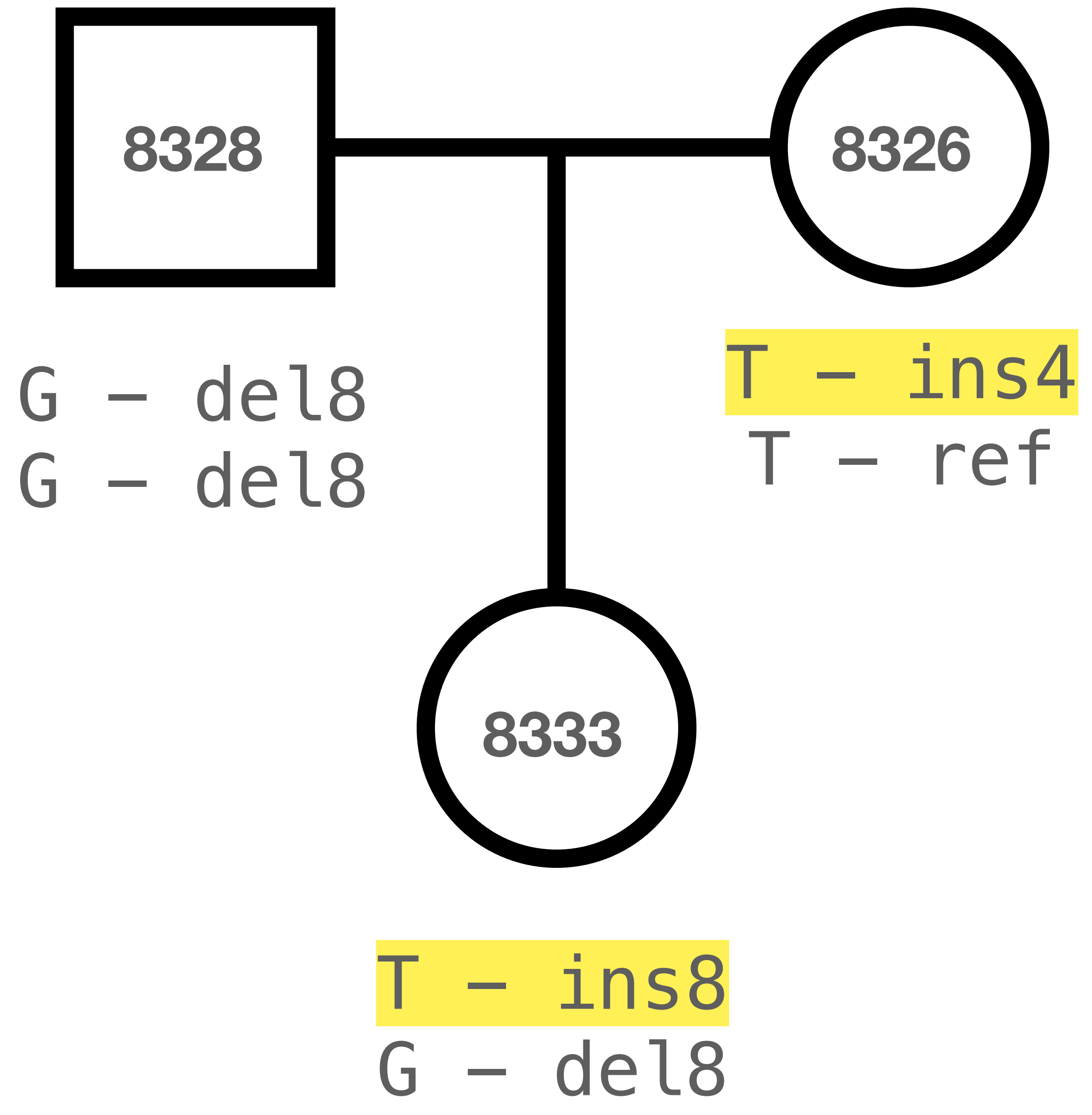

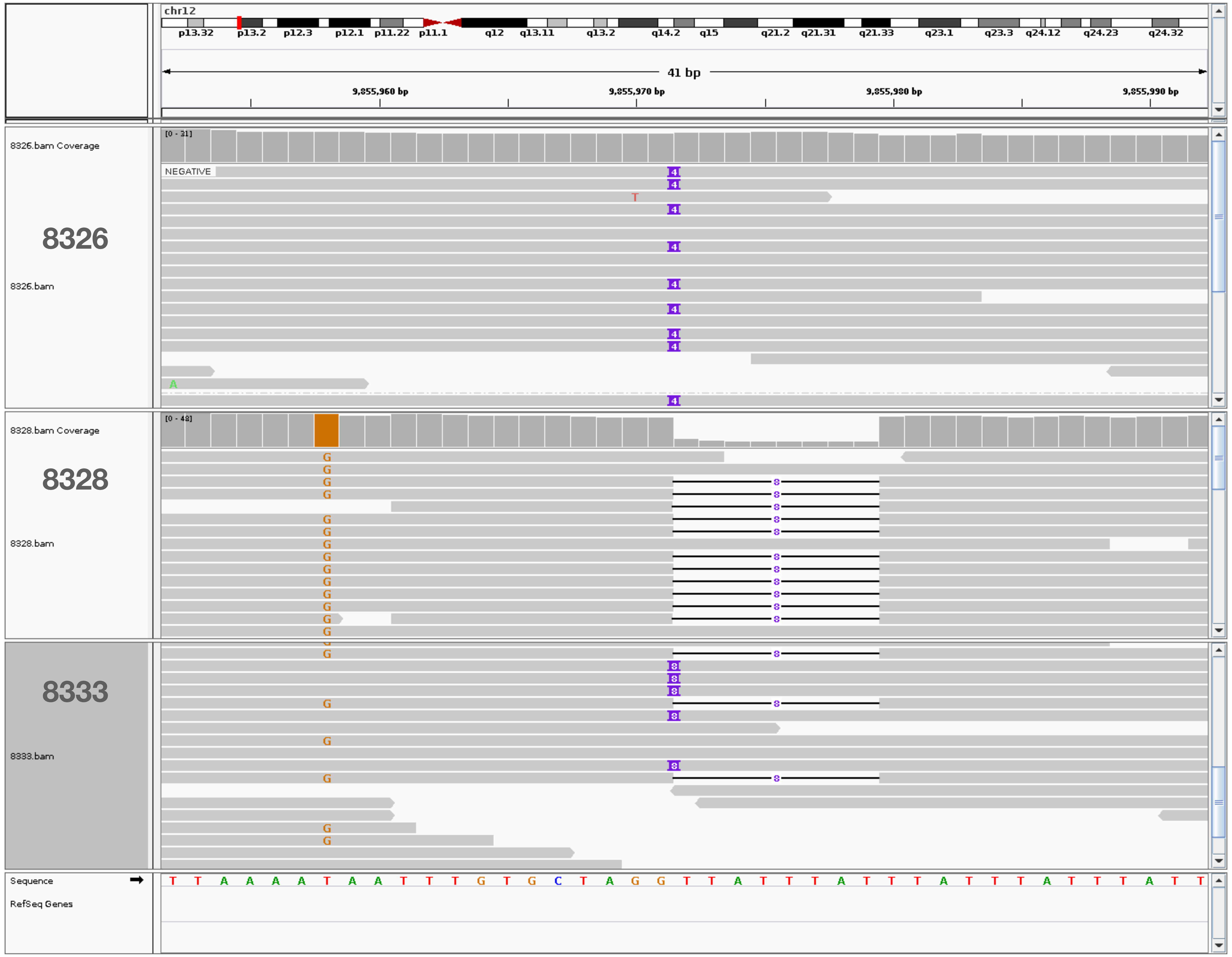

1345 8333 12:9855972 8326q female 2 41 3 45 1 4 1 [0.602941, 0.485294, 0.602941, 0.794118] 19 50000 4

De novo 13

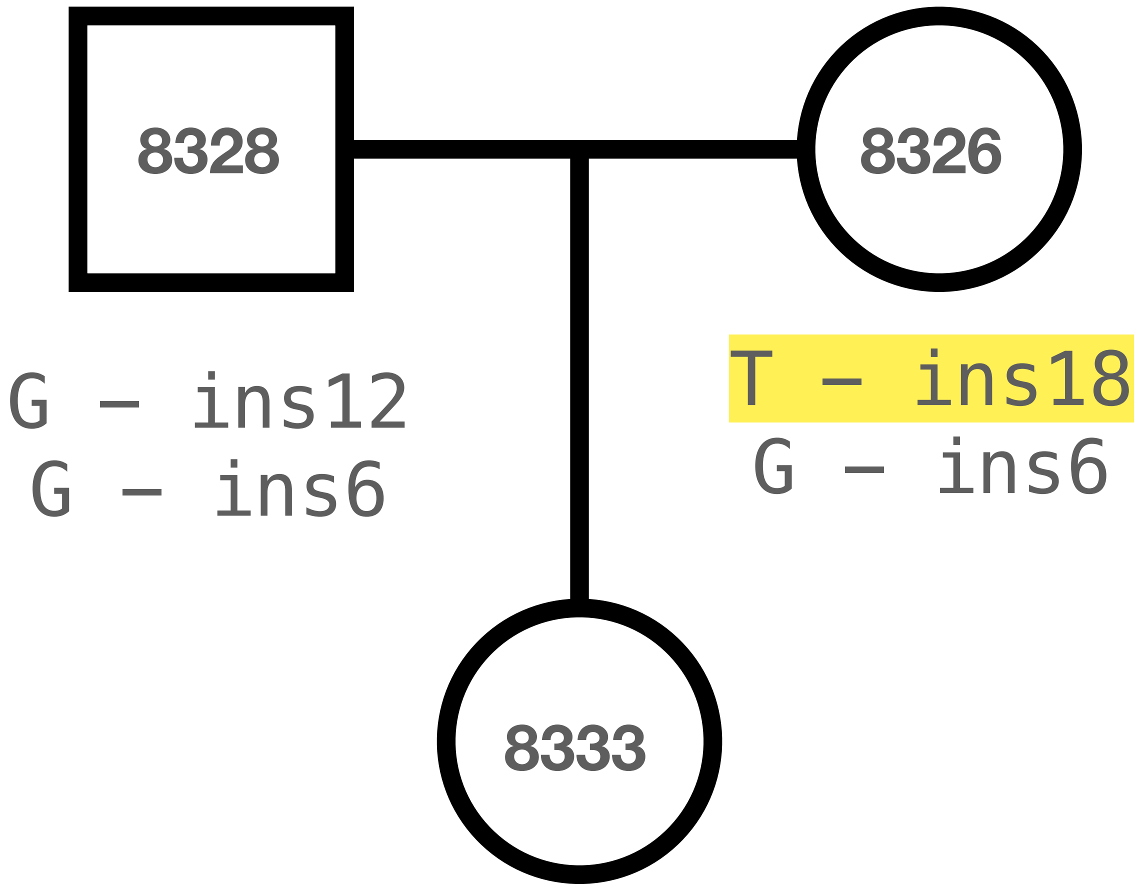

G – ins12
G – ins6

T – ins18
G – ins6

T – ins21
G – ins6
(T – ref)

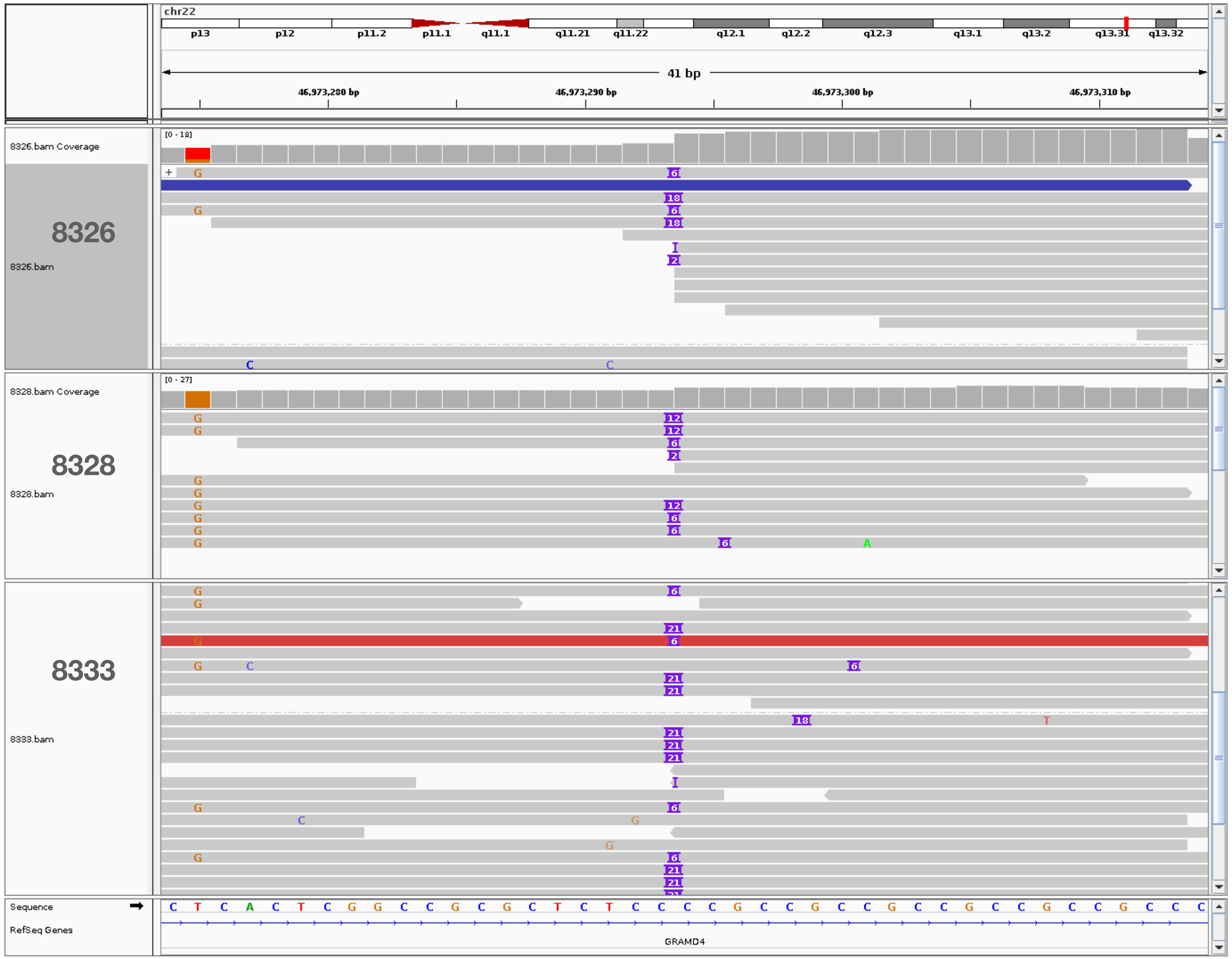

1345 8333 22:46973294 8326p female 1,4 26,38 5 41 NaN NaN 3 [0.705021, 0.629707, 0.976987, 0.75523] 22 240000 3

De novo 14

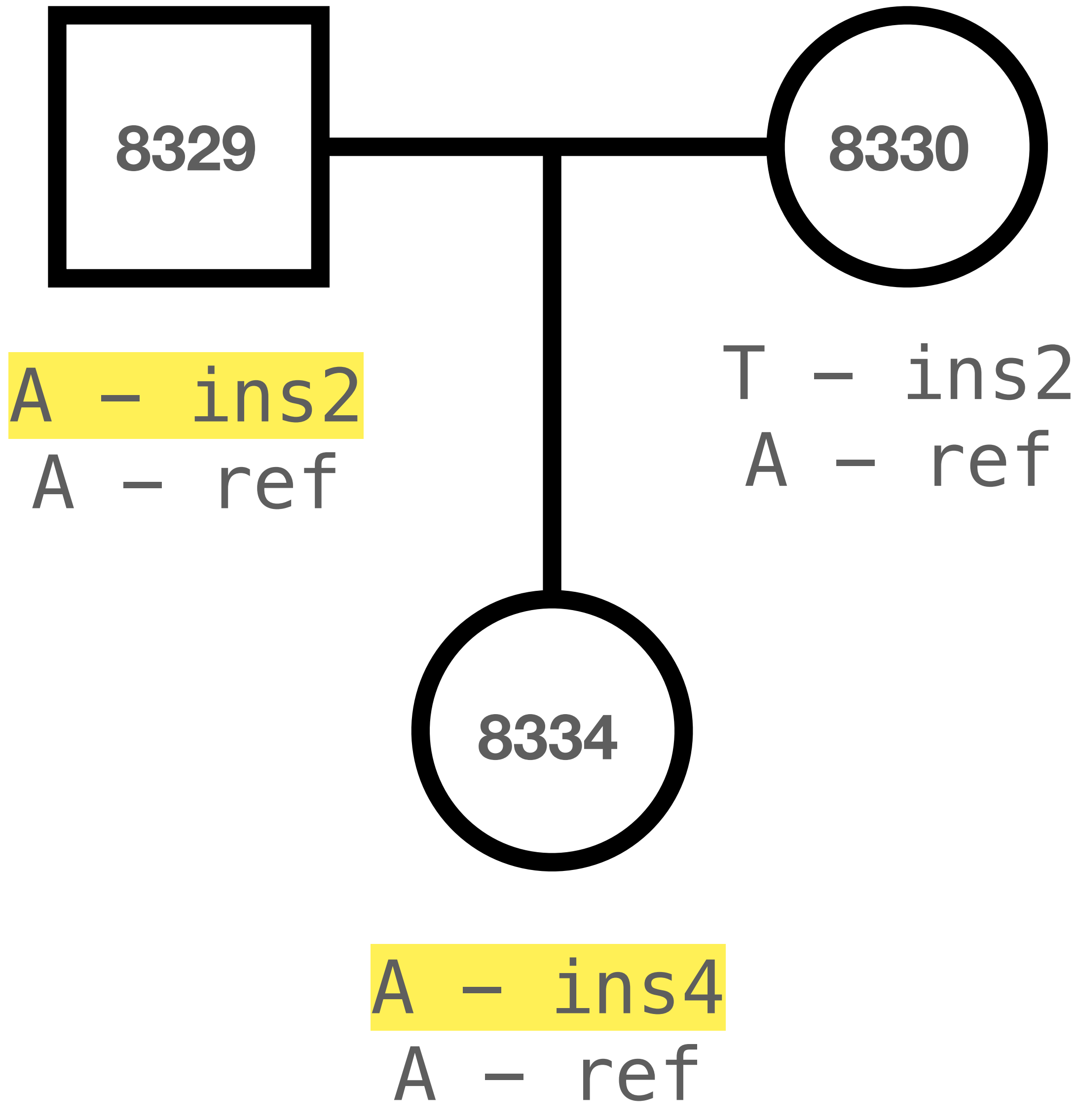

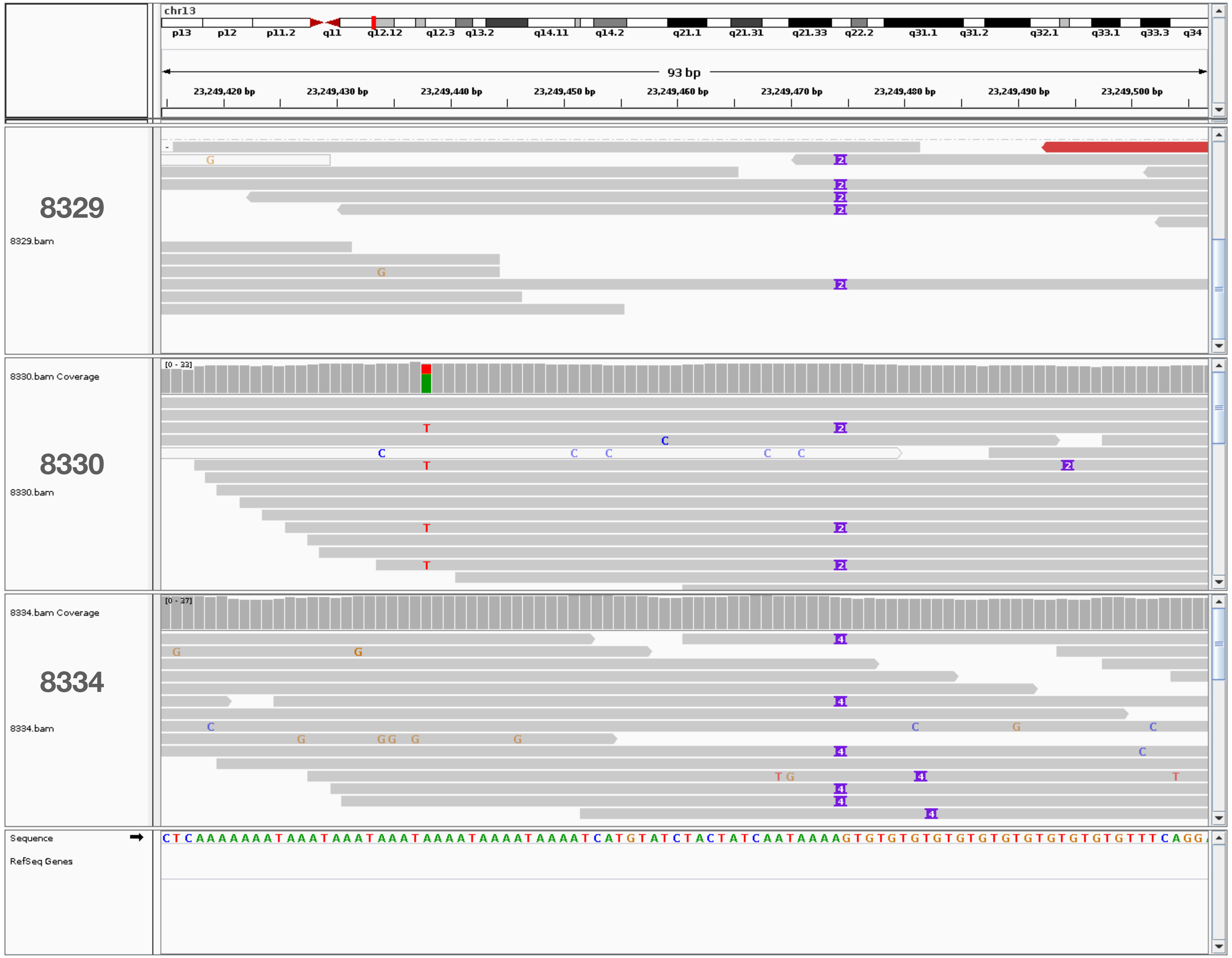

Most likely ins2 -> ins4

1345 8334 13:23249475 8329q male 1 28 2 30 1 2 1 [0.780282, 0.96338, 0.752113, 0.791549] 17 260000 2

De novo 15

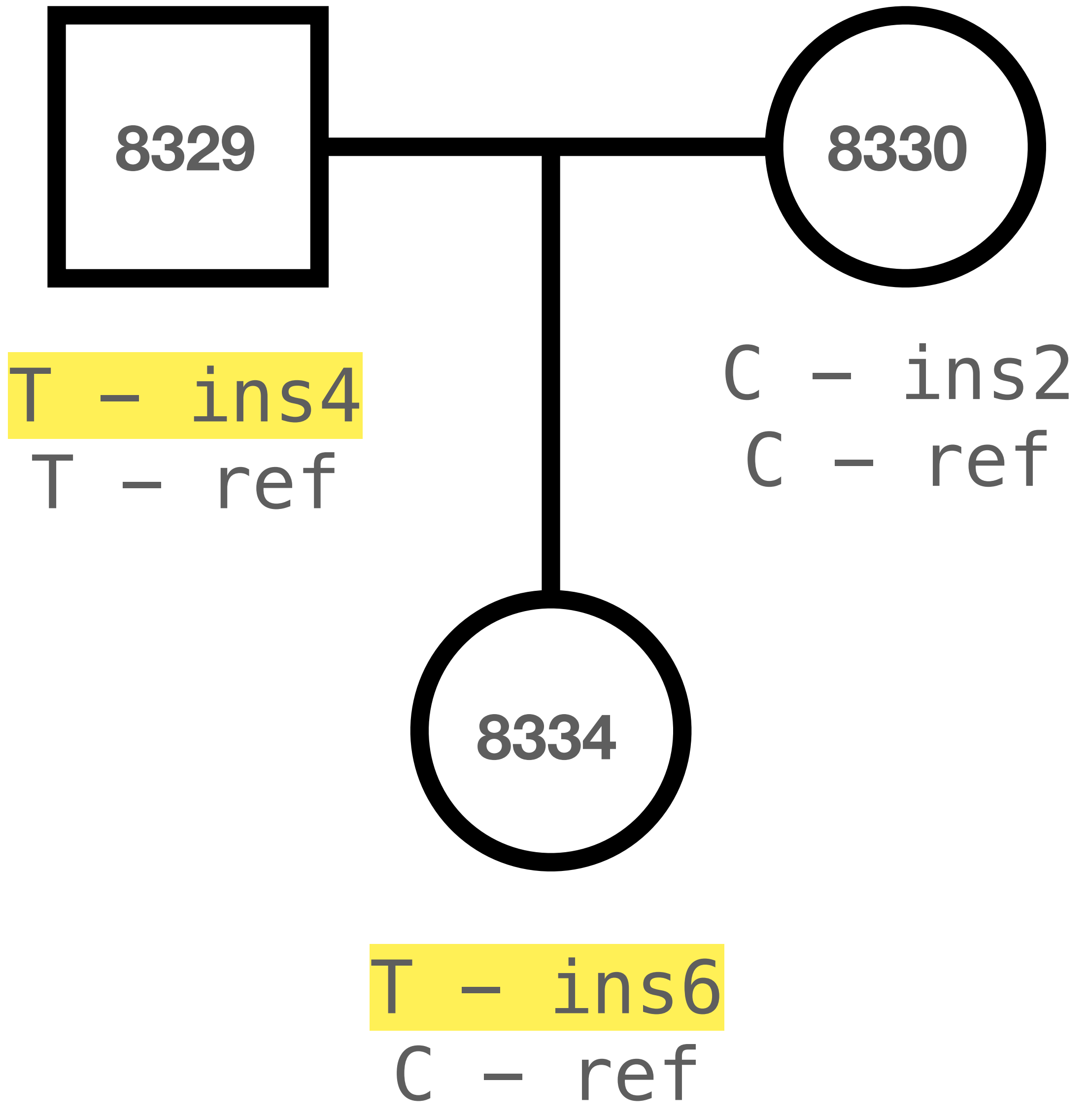

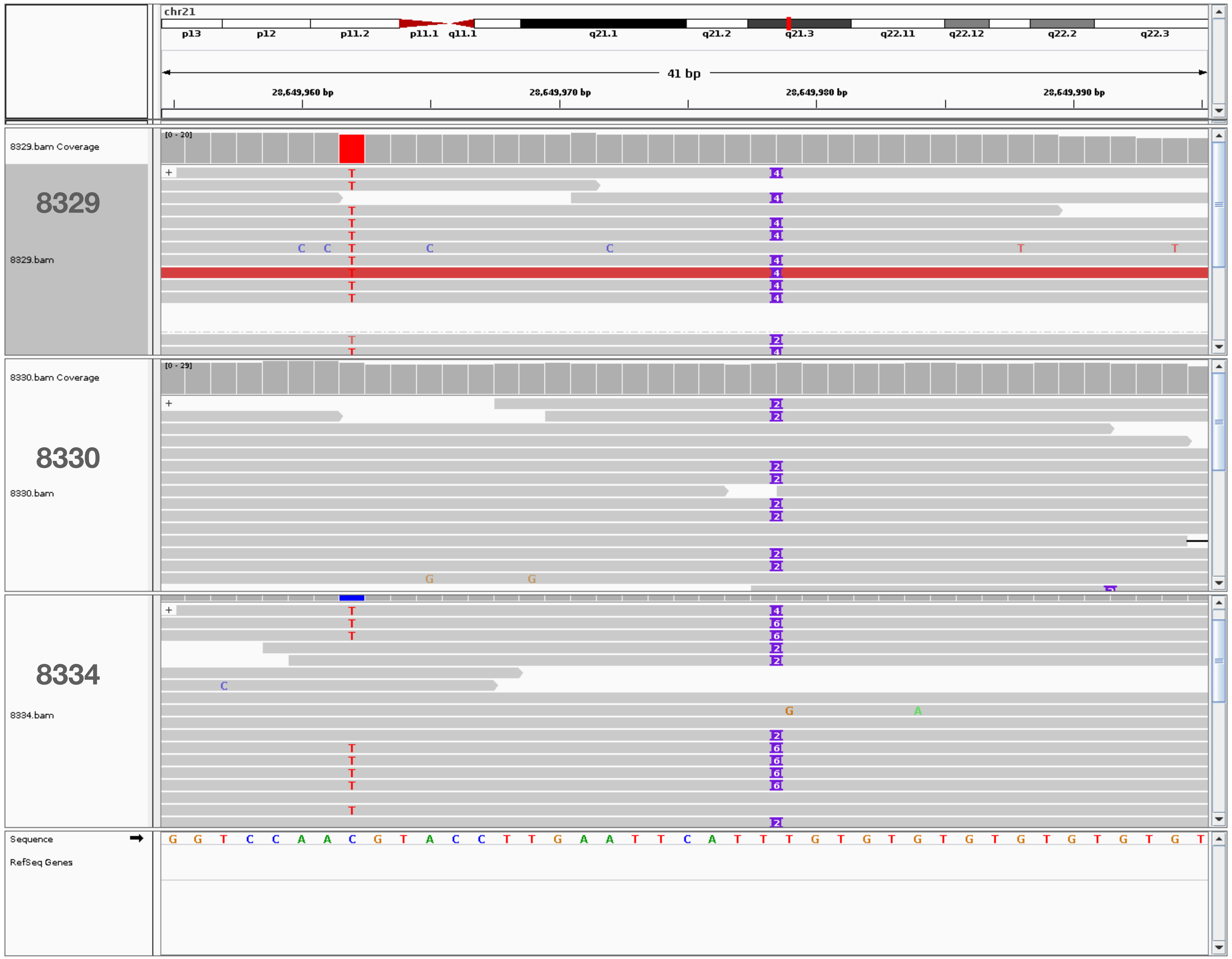

1345 8334 21:28649979 8329q male 2,3 36,36 4 38 NaN ±2 6 [0.779412, 0.970588, 0.823529, 0.779412] 15 40000 2

De novo 16

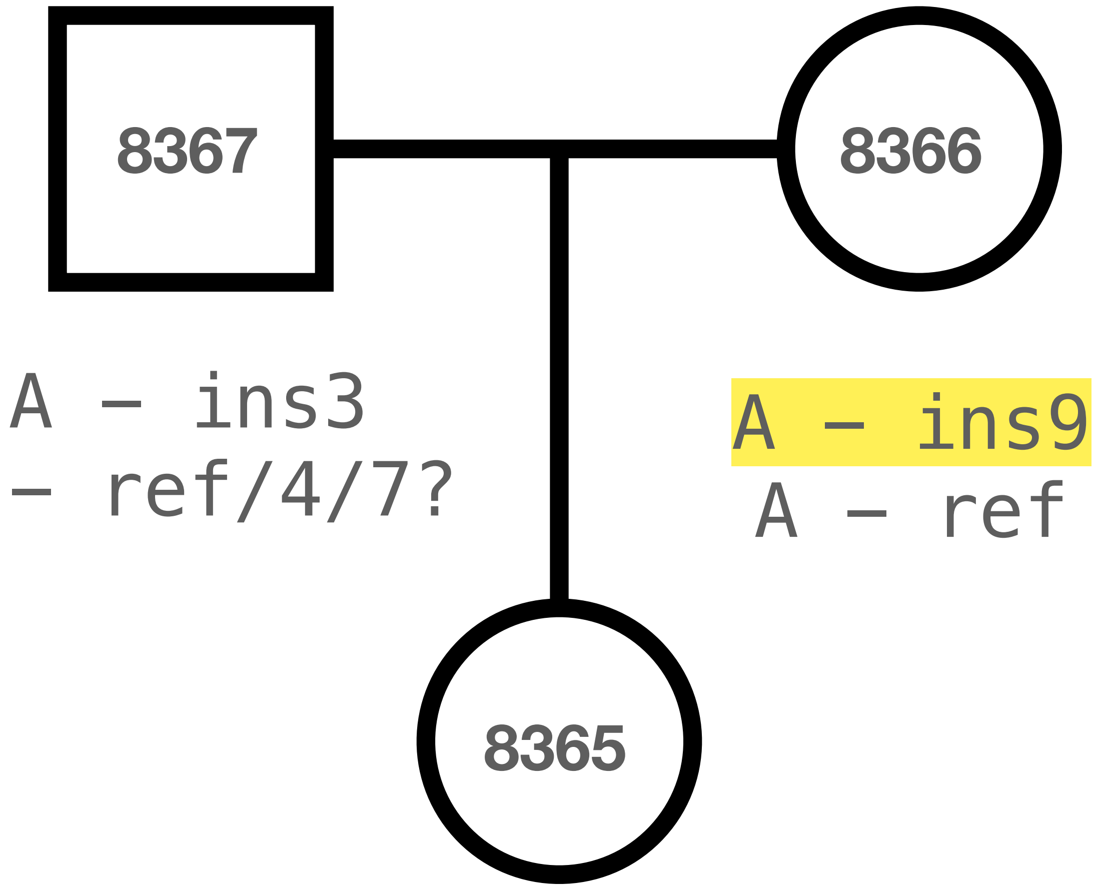

A – ins3
G – ref/4/7?

A – ins9
A – ref

A – ins12
A – ins3

Most likely ins9 -> ins12

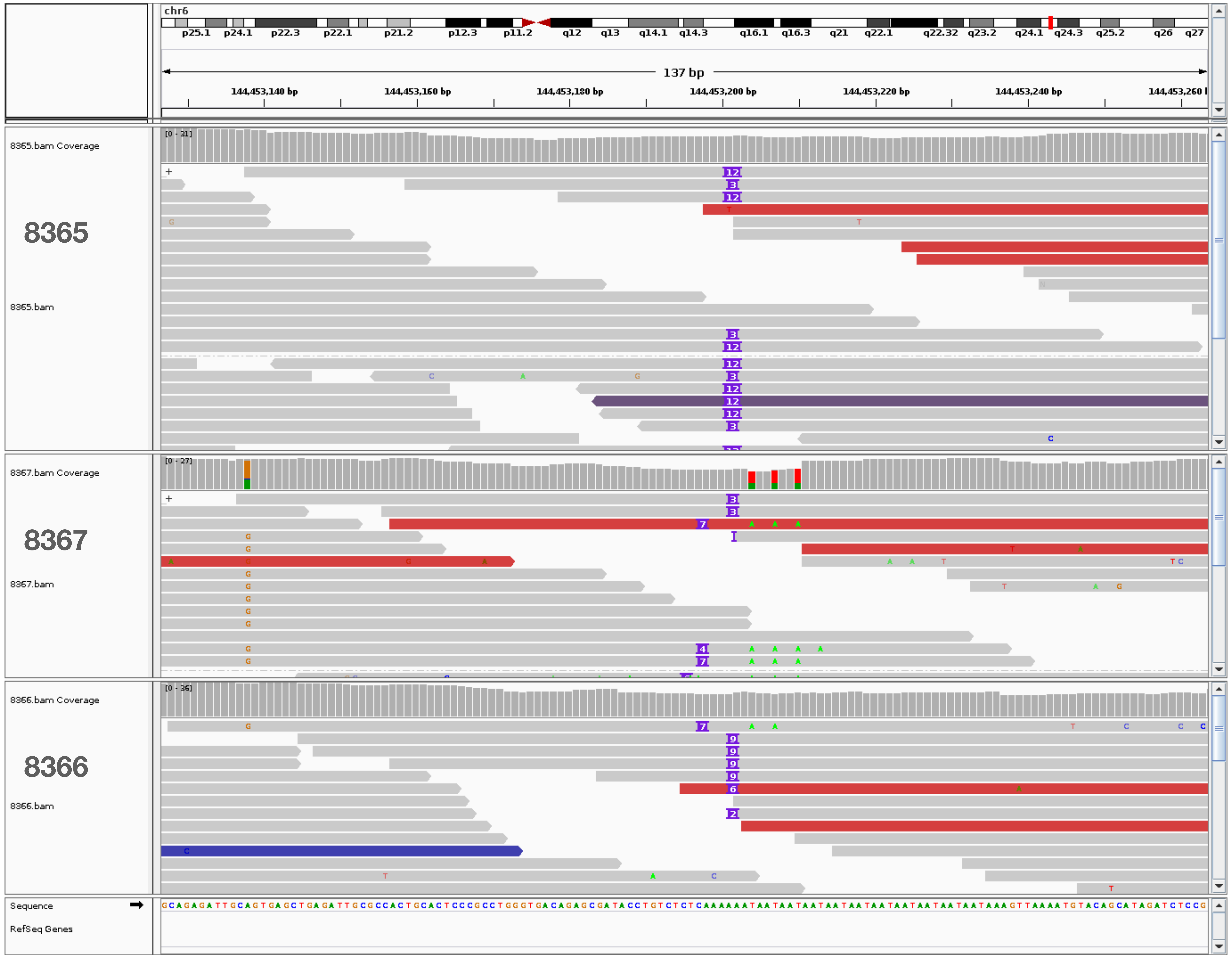

1346 8365 6:144453196 8366p female 3 50 4 53 1 3 1 [0.436464, 0.59116, 0.911602, 0.370166] 32 290000 3

De novo 17

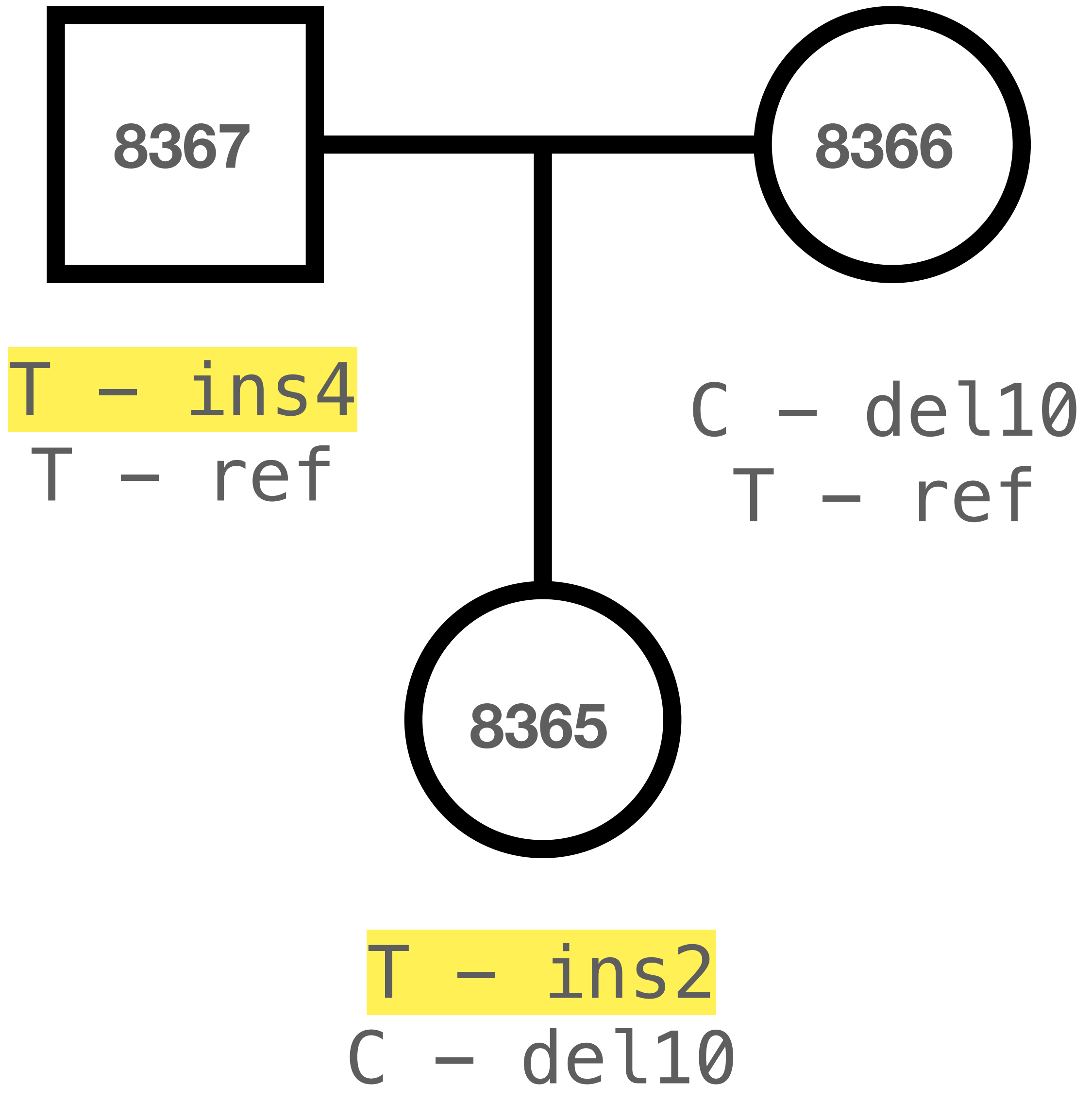

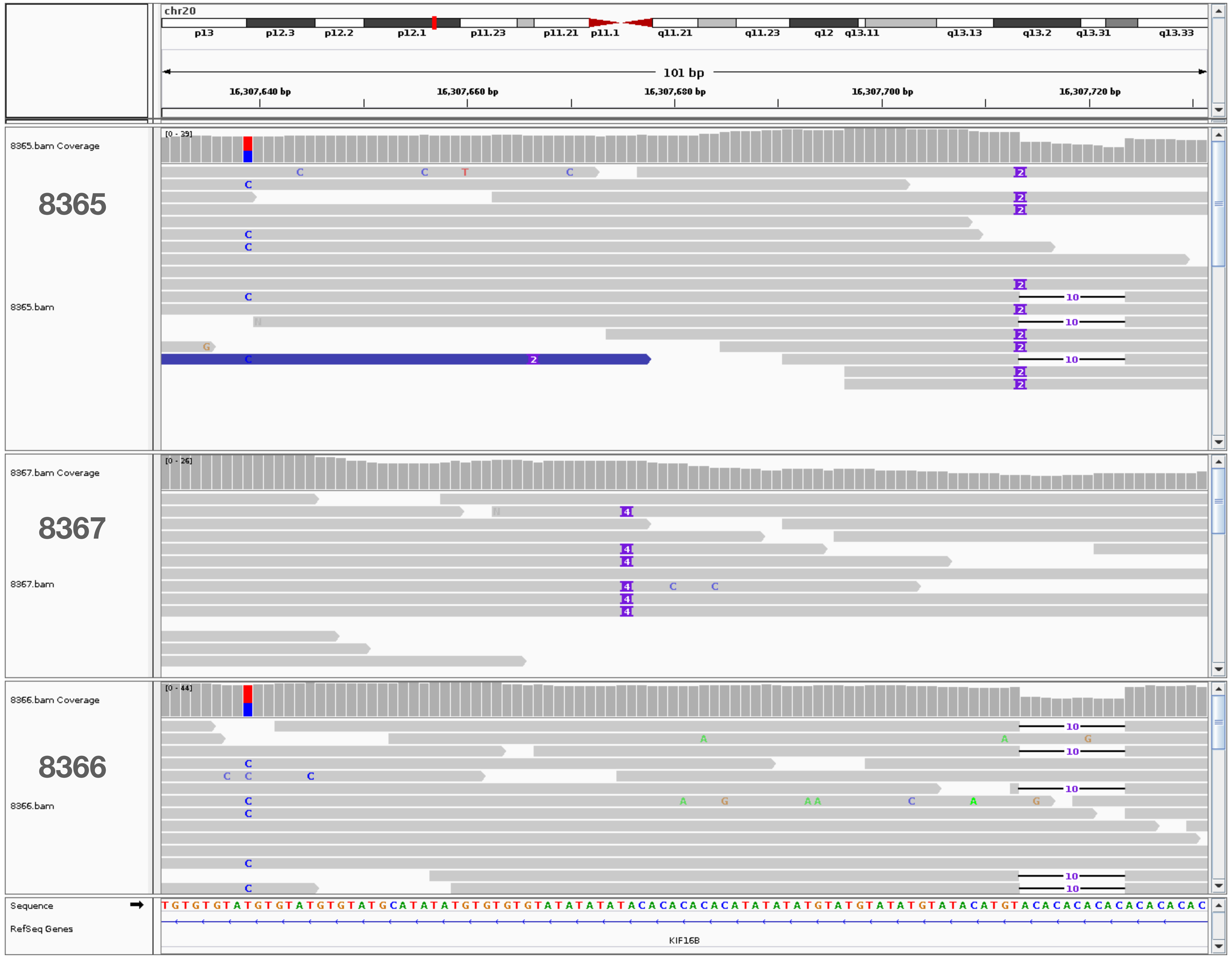

1346 8365 20:16307714 8367q male 0 34 2 36 2 2 3 [0.48433, 0.980057, 0.518519, 0.236467] 46 140000 2

De novo 18

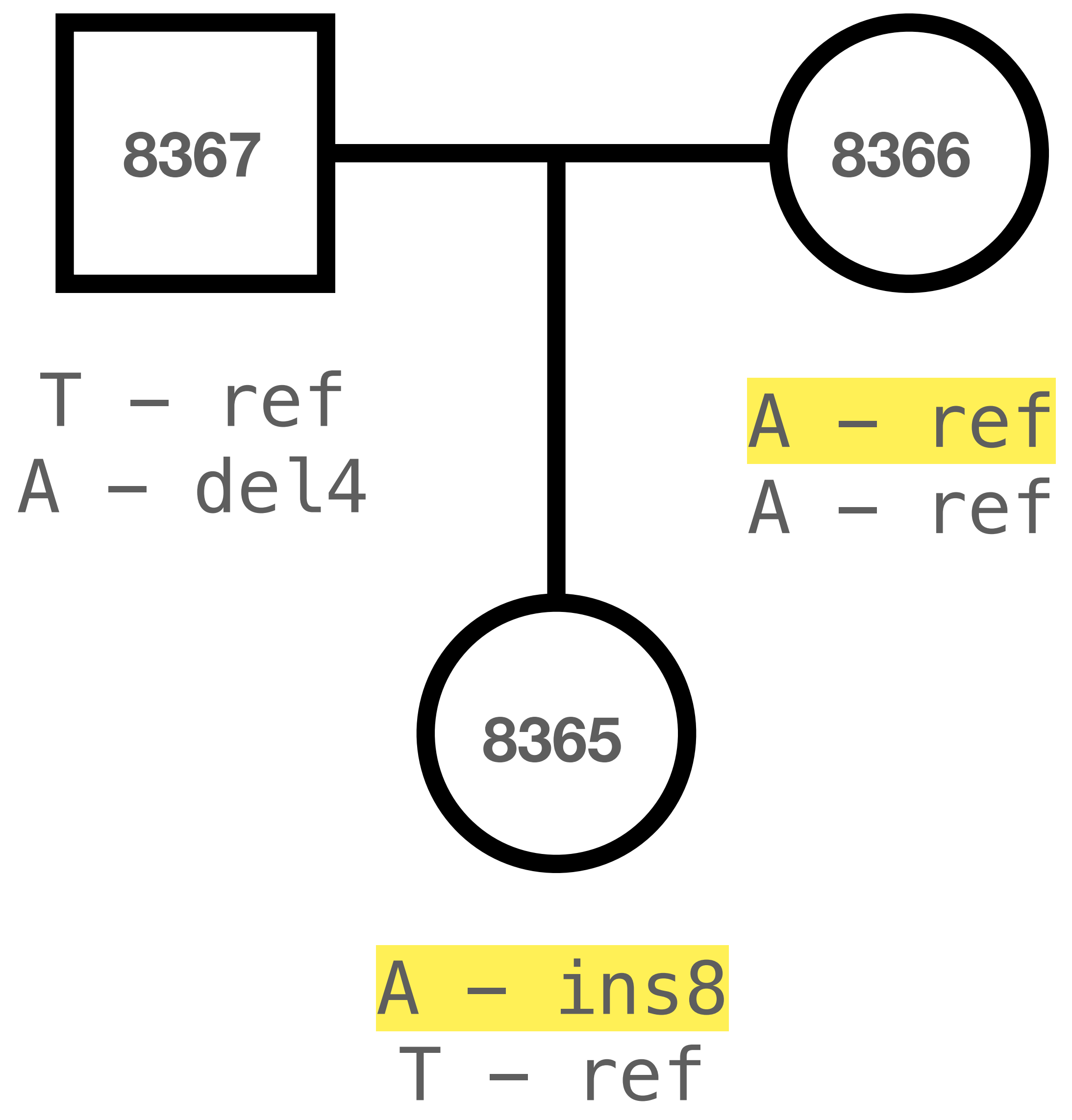

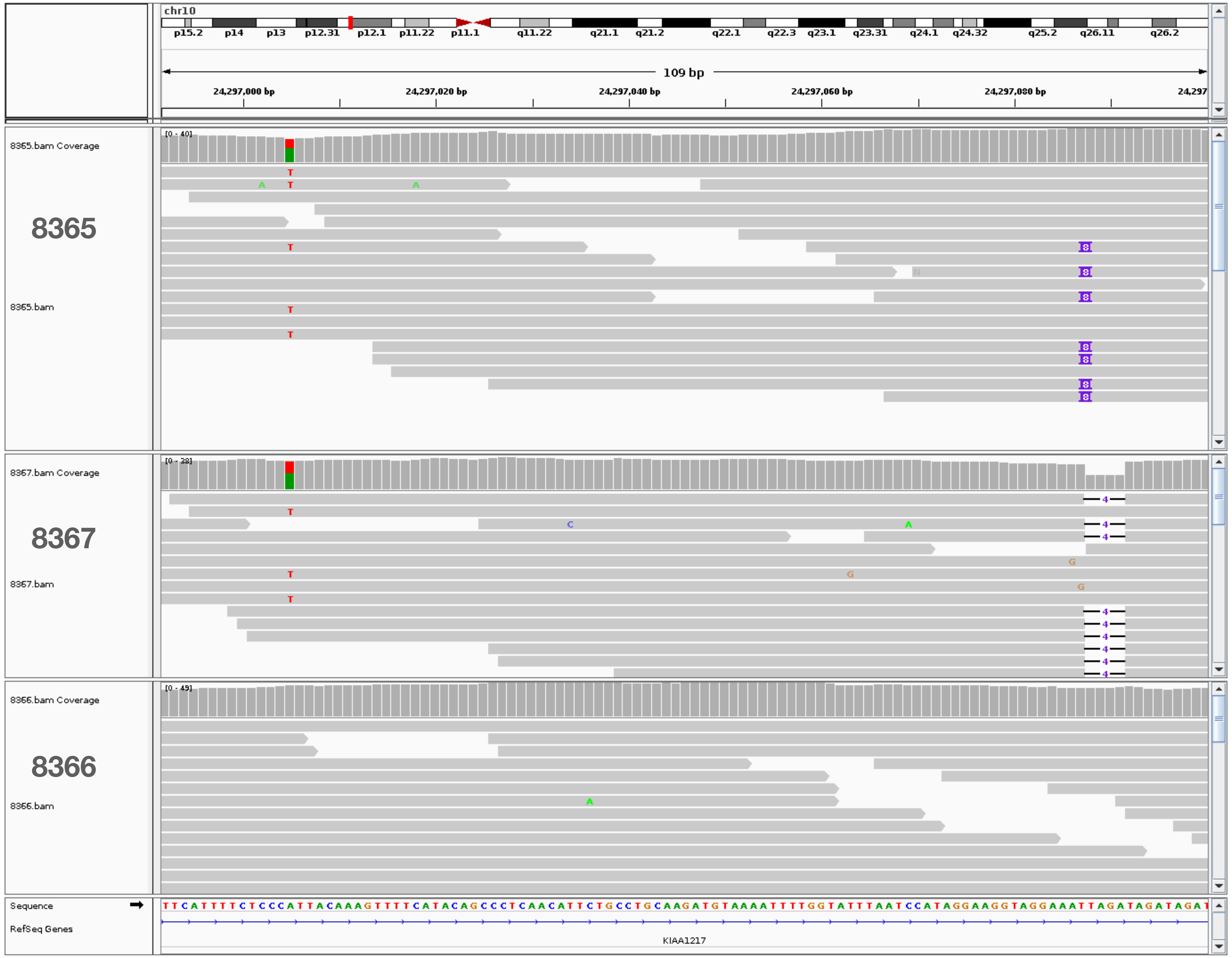

1346 8365 10:24297088 8367p male 1,2 45,53 3 57 NaN NaN 3 [0.973214, 0.348214, 0.205357, 0.285714] 62 90000 4

De novo 19

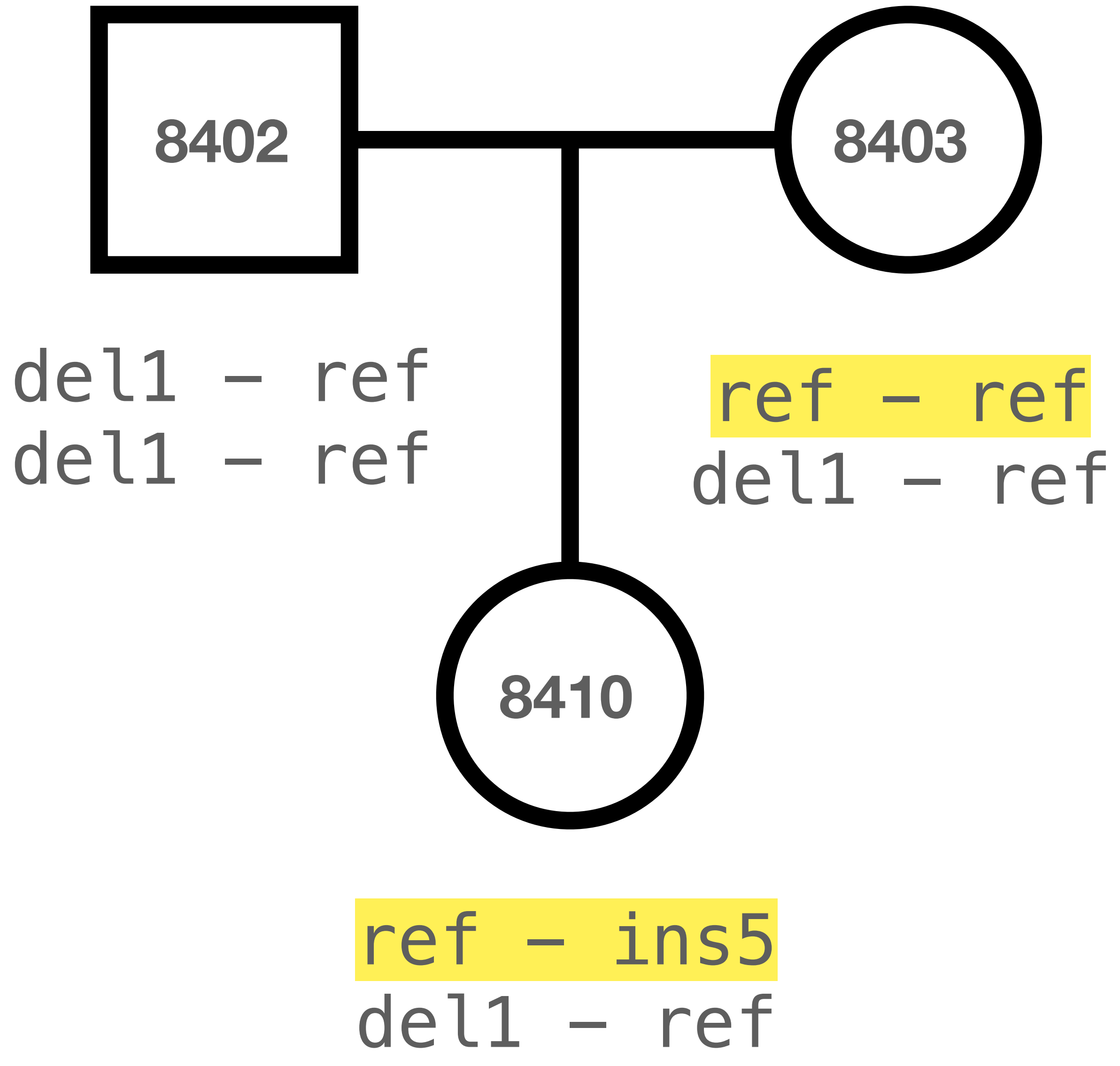

del1-ref in father must come from male gp

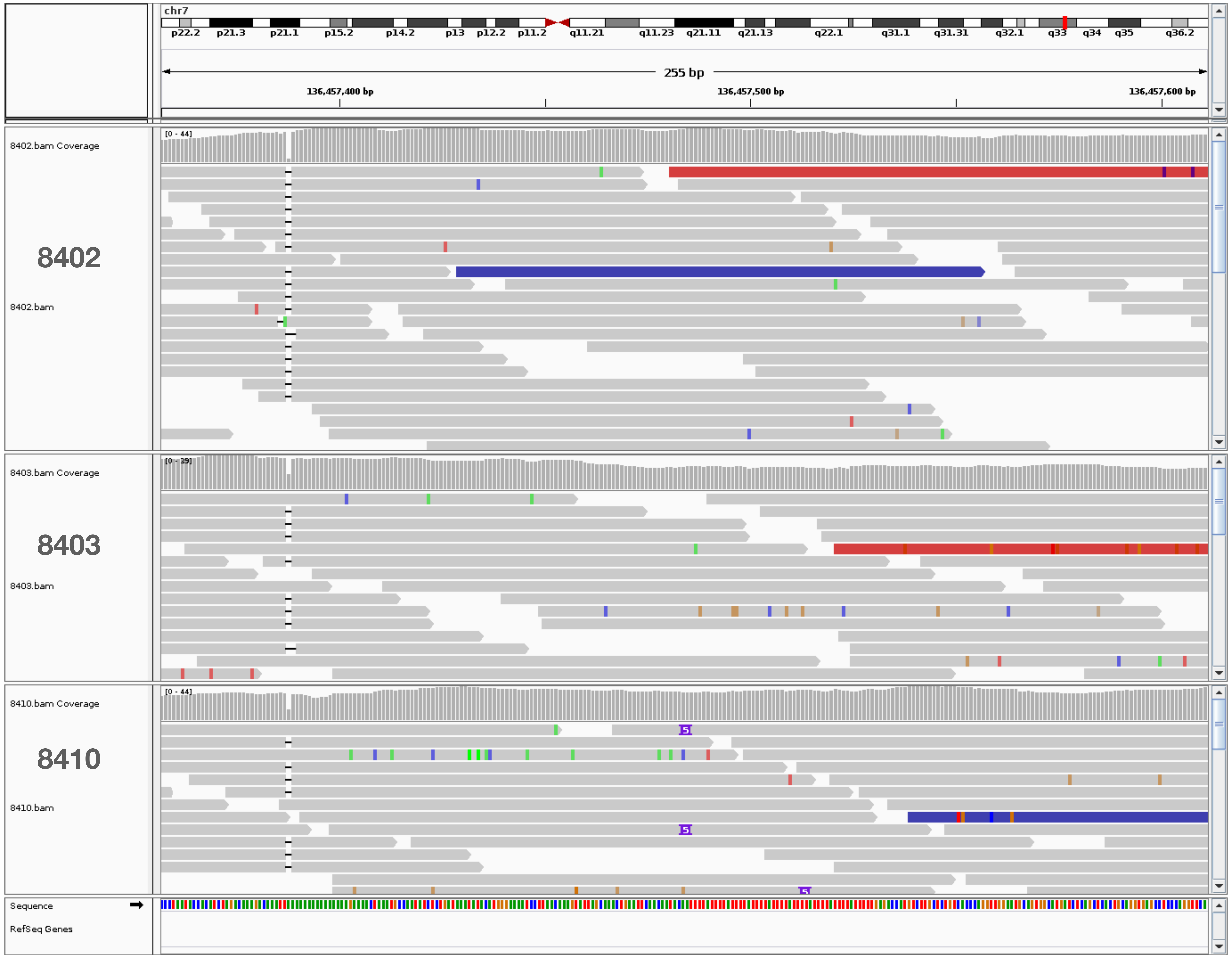

1350 8410 7:136457485 8403p female 0 45 1 50 1 5 5 [0.021277, 0.021277, 0.994681, 0.021277] 97 50000 5

De novo 20

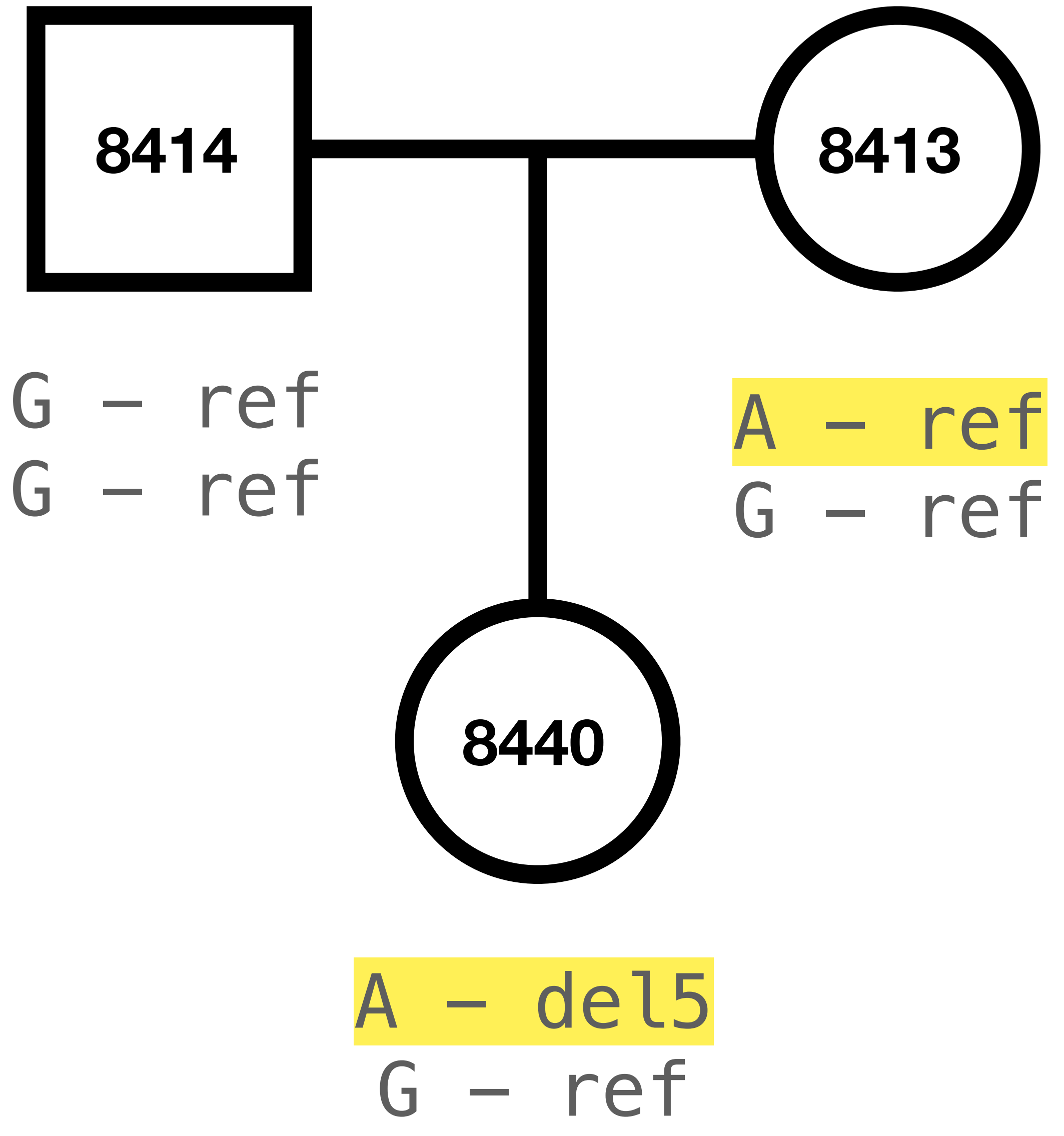

(One del5 read found in mother; mother's genotype is ref)

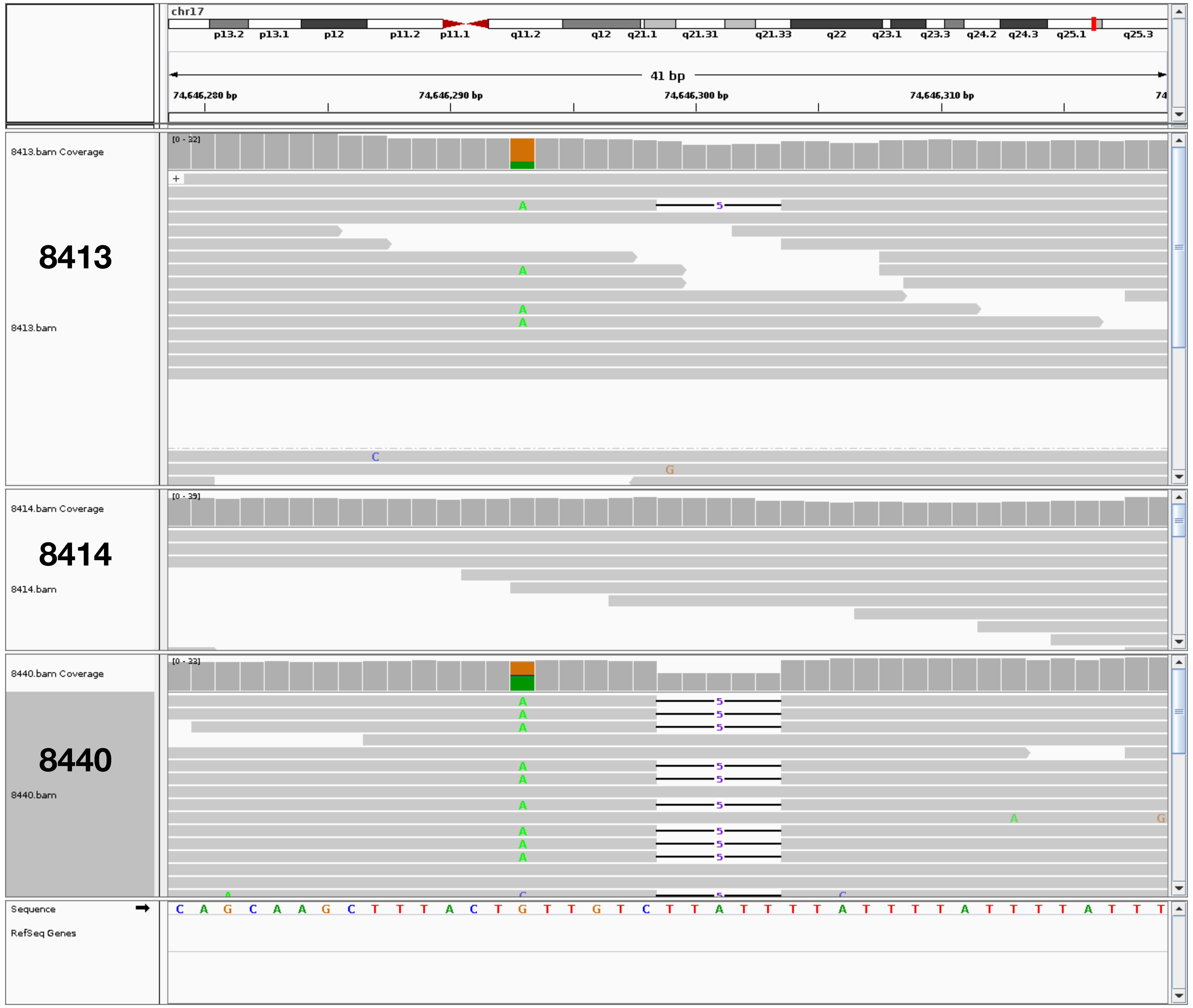

1347 8440 17:74646299 8413q female 0 32 1 27 1 -5 5 [0.420354, 0.371681, 0.415929, 0.911504] 49 50000 5

De novo 21

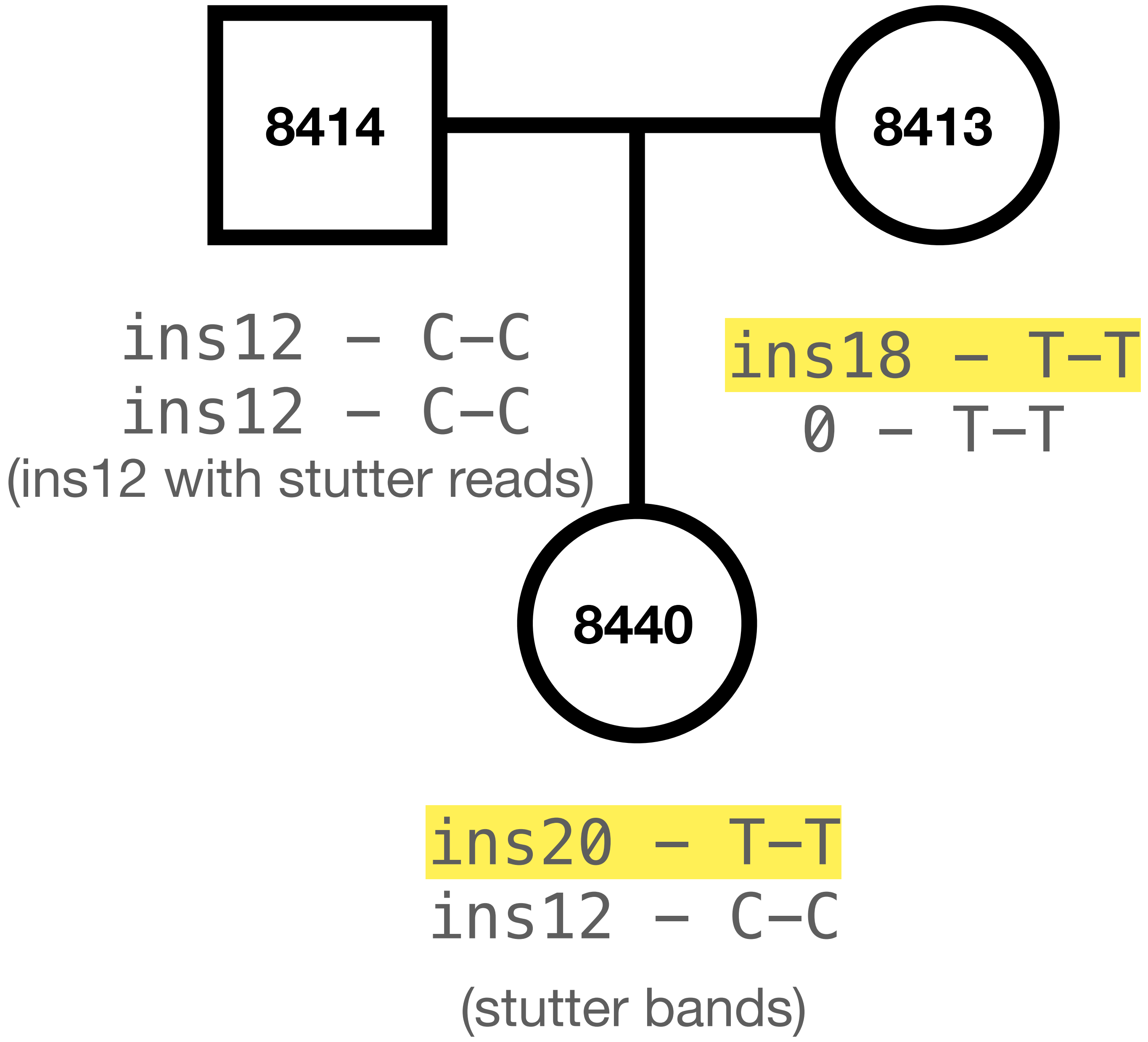

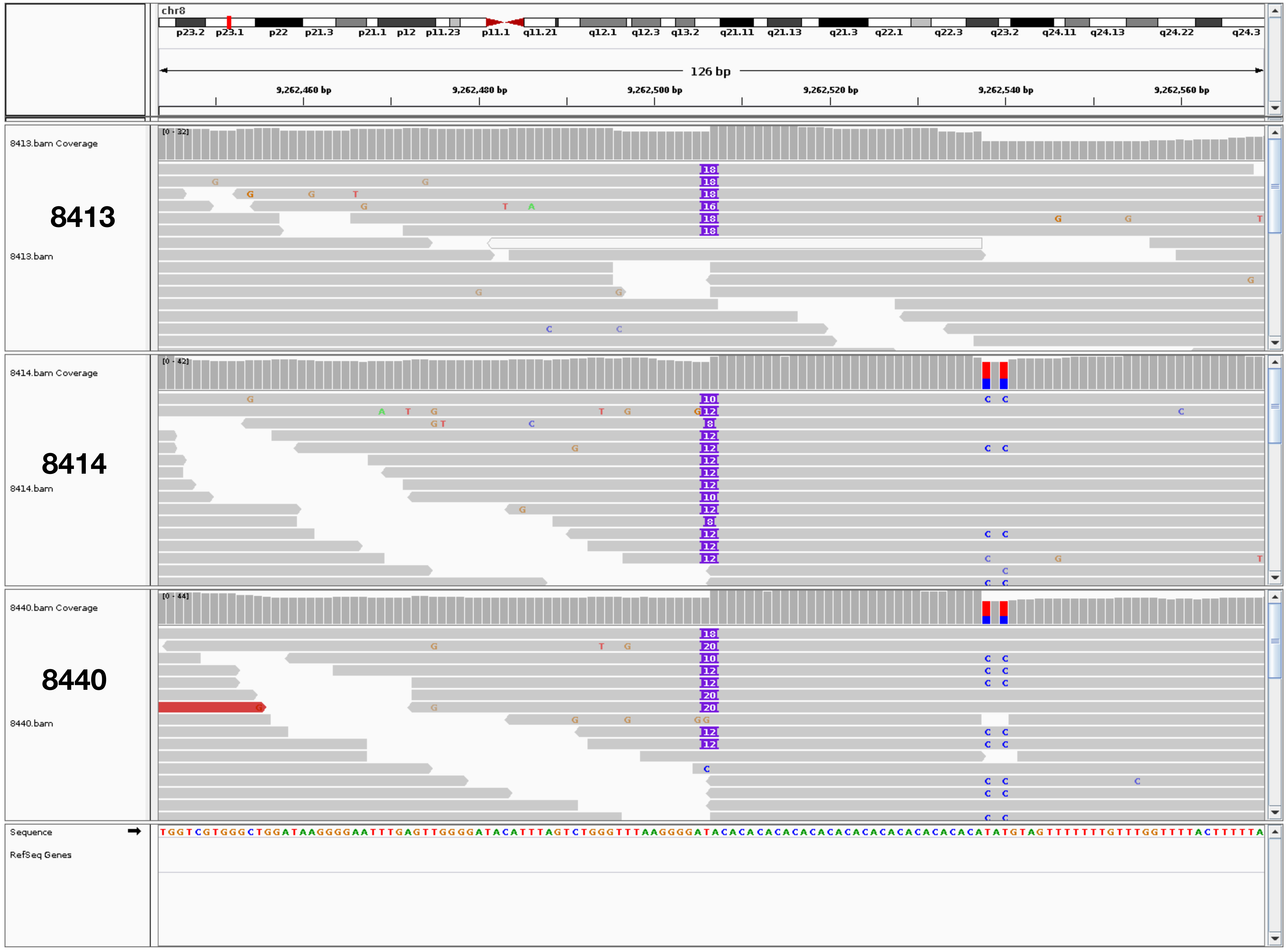

1347 8440 8:9262507 8413p female 5 52 6 54 1 2 6 [0.69877, 0.8125, 0.938525, 0.795082] 13 260000 2

De novo 22

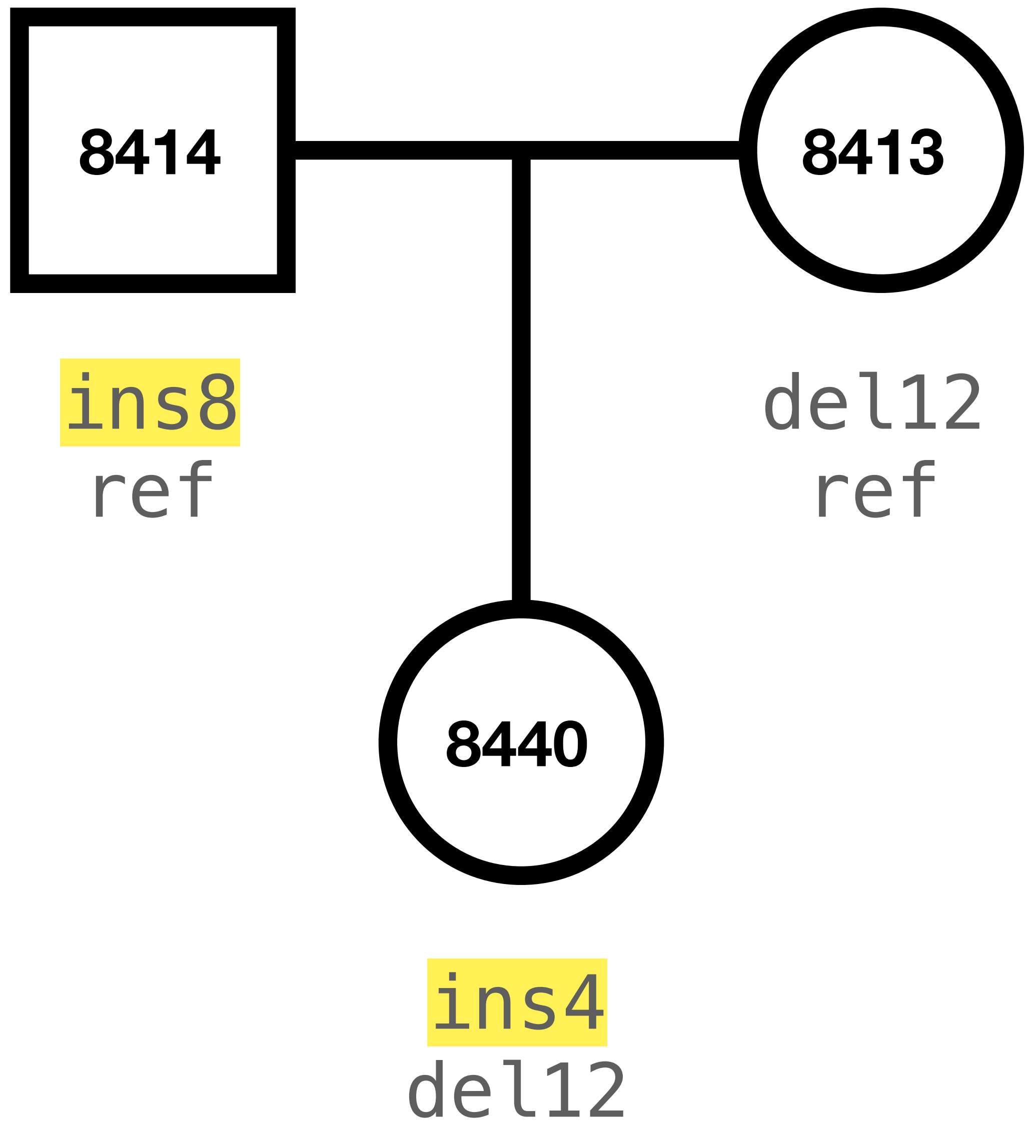

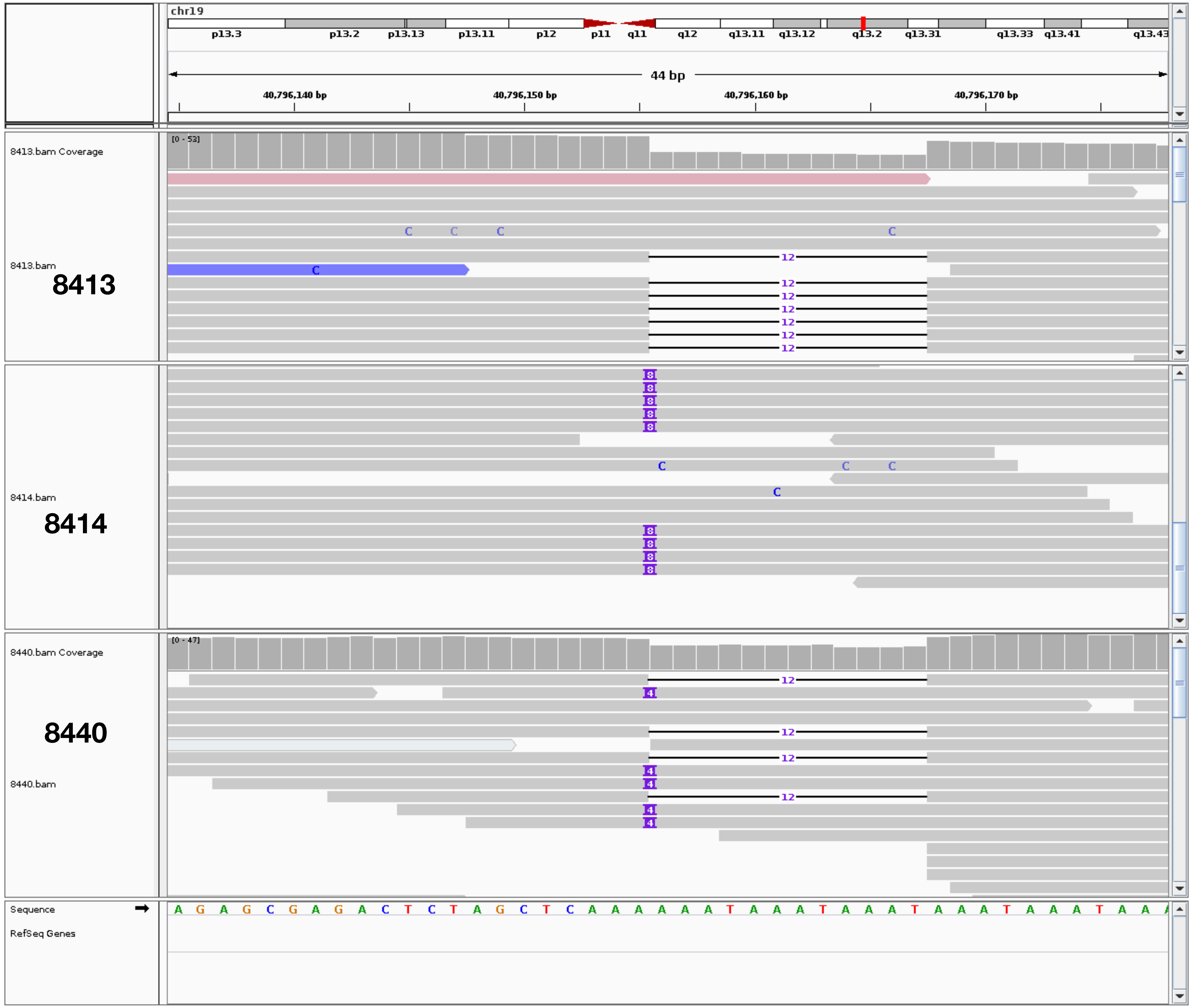

1347 8440 19:40796156 8414p male 3 43 2 39 -1 -4 6 [0.810909, 0.541818, 0.541818, 0.578182] 23 290000 4

De novo 23

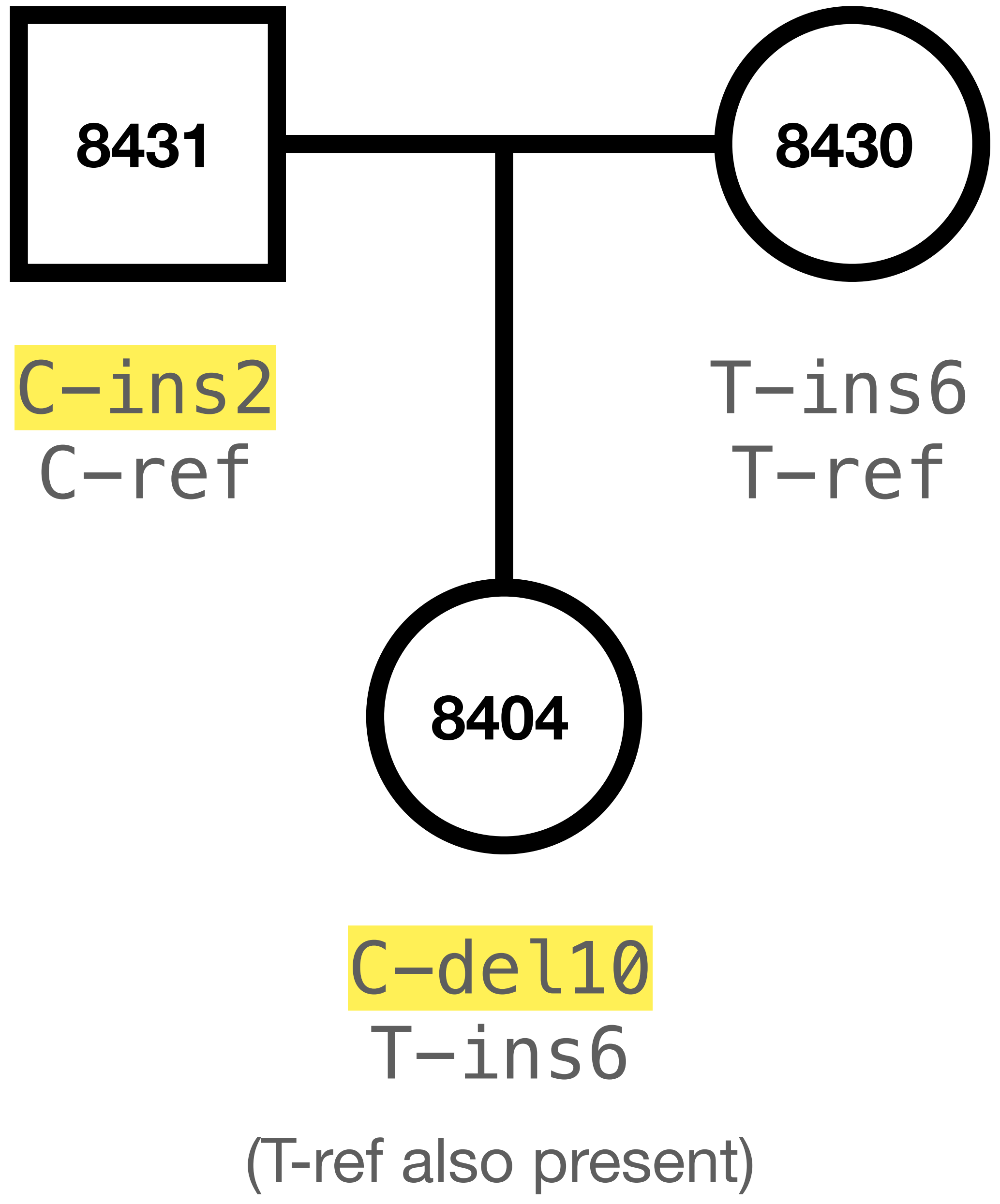

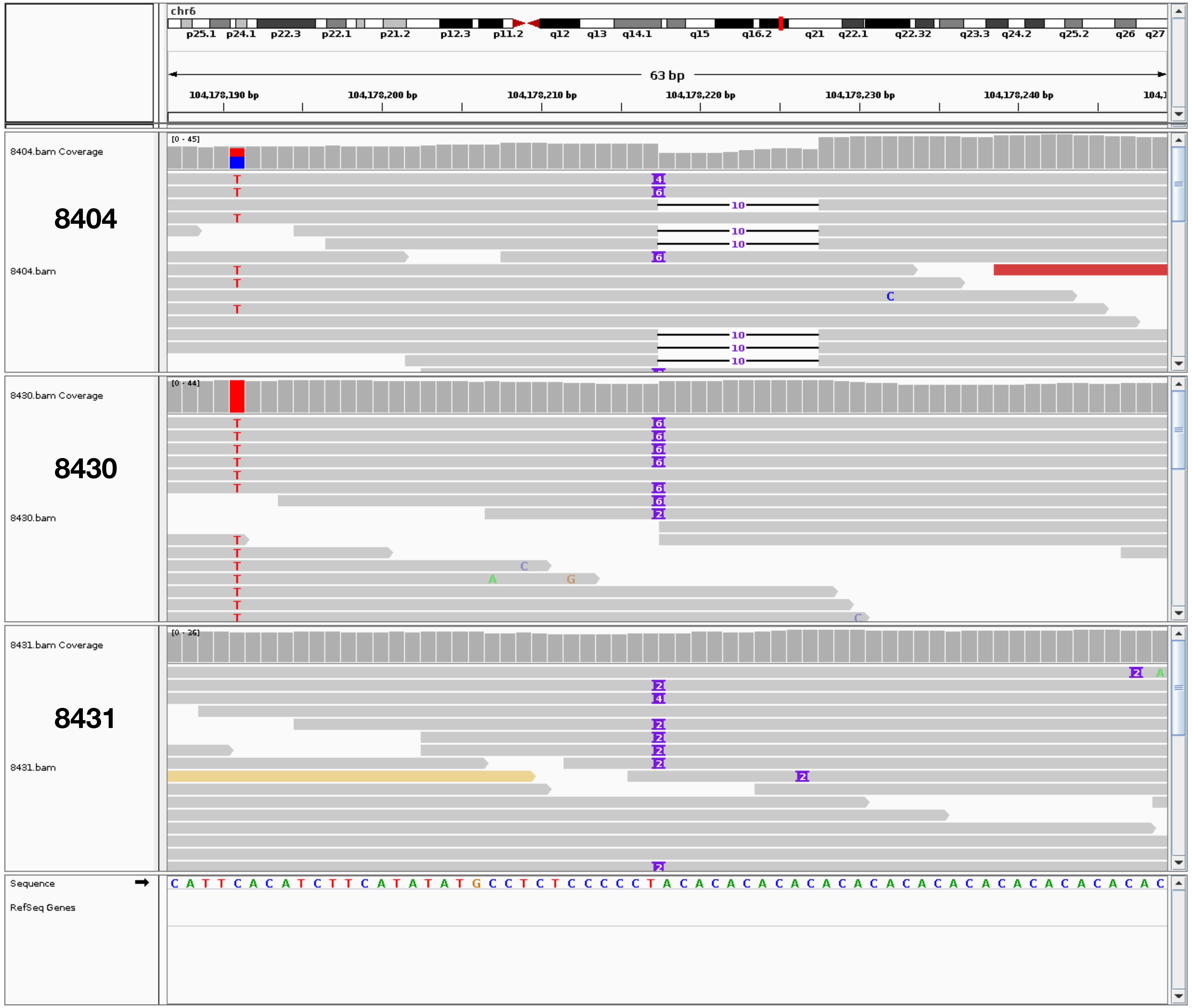

1350 8404 6:104178218 8431p male 3 59 1 47 -2 -12 1 [0.983871, 0.774194, 0.822581, 0.822581] 16 20000 2

De novo 24

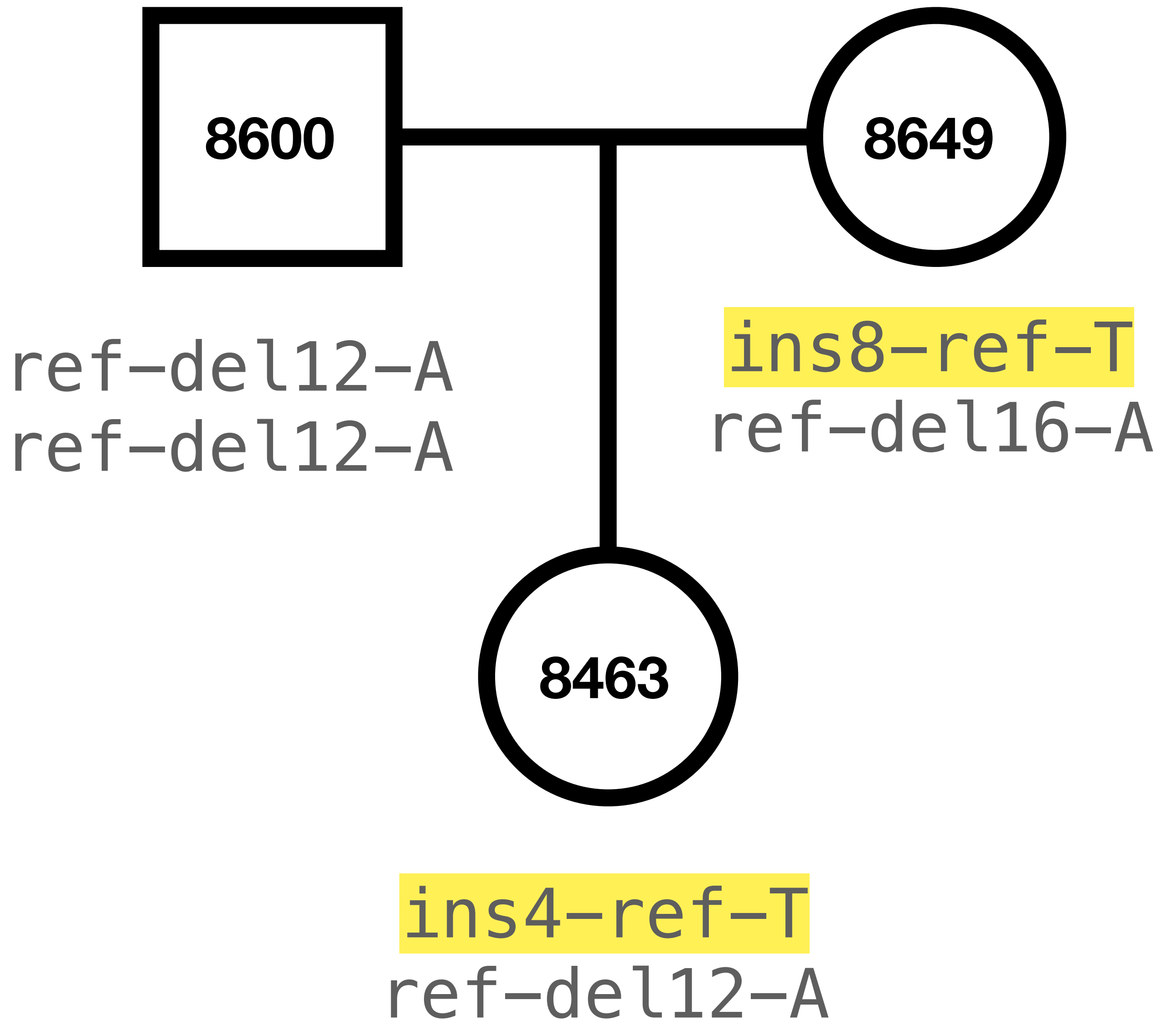

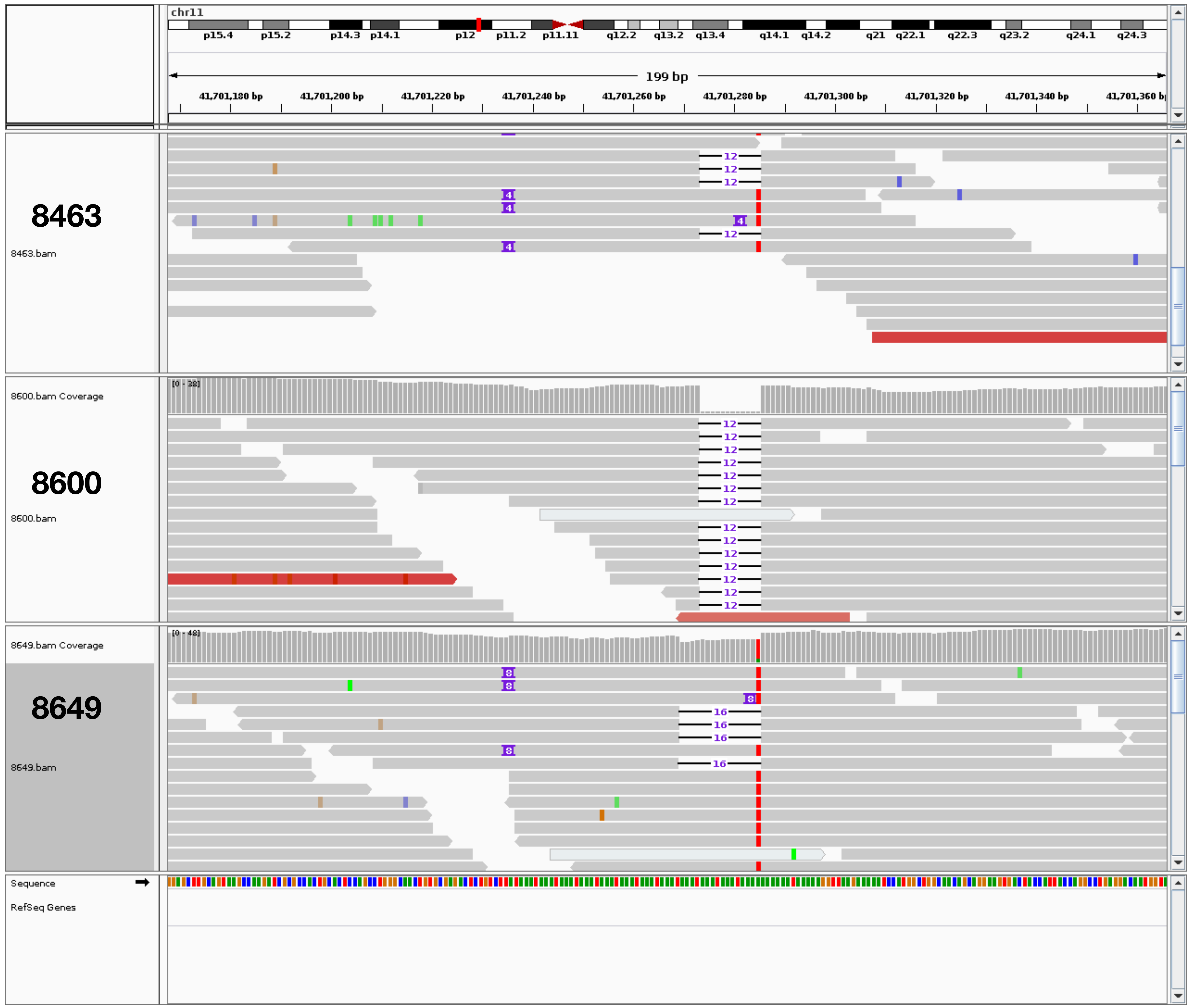

1353 8463 11:41701236 8649p female 1,4 44,68 3 64 NaN NaN 5 [0.079365, 0.079365, 0.984127, 0.079365] 90 20000 4
