# Supplementary material for: The mutational dynamics of short tandem repeats in large, multigenerational families
Source: Genome Biol. 2022 Dec 12;23:253. doi: 10.1186/s13059-022-02818-4 (PMC9743774; doi:10.1186/s13059-022-02818-4)
Supplement: Supplementary file 7 — Additional file 7. [file 13059_2022_2818_MOESM7_ESM.docx]

**1^st^ round**

**Reviewer 1**

Steely and colleagues perform a comprehensive study examining differences mutation rates depending on STR repeat length and testing for age and parent-of-origin effects. They analyze a large cohort of multi-generational pedigrees which permits evaluation and confirmation of repeat slippage across generations and can be compared to a subset of STRs that have already been genotyped in these families. They are able to filter out a high degree of false positives (~20%) and find some individuals with large numbers of mutations. Some of these findings may be due to using samples that were not sequenced in a PCR-free manner.

The authors have a good strategy for validating findings in the third generation of samples, but it is unclear whether variability in the first generation is as stringent. For example, what if a grandparent in the first generation was genotyped incorrectly or had a somatic change? How would these false positives be caught?

The patterns in Figures 3A, 4A and 4B seem a little difficult to explain. The authors provide some rationale but a more formal analysis appears warranted. There seem to be a couple of different factors that contribute: mutations within Alu repeats, and repeat expansion/contractions elsewhere in the genome. The authors could do a more extensive evaluation trying to delineate some of the various possibilities for the distribution. It appears that mono-nucleotide mutations within Alu elements play a substantial role in the distribution of repeat alleles, it would be helpful to determine how much of an effect they have by separating some of the analyses in figures 3 and 4 out between those that are within Alu repeats and those that are not (especially mono-nucleotide repeats in 3A and 4A). Another option would be to list the number of repeats of each overall and pure repeat length (whether or not they are mutated) to get a better sense of this distribution of the denominator and/or list mutations rate individual for A, C, G and T nucleotides. For example, are there many more mono-nucleotide repeats of length ~15, and are these mostly polyA repeats, and are these mostly within Alu elements?

Likewise, the data in figure 3B show an expected pattern for pure repeats, but in the comparable plot in figure 4B, the mutation rate value drops off after a repeat length of ~40. Is the rate of interrupted motifs much higher, and does this therefore decrease the possibility of mutation? Also, is there a bias in the nucleotide composition for repeats that have a high mutation rate (e.g. AT-repeats) versus those that have a longer repeat length but a lower frequency of mutation? Or is it again related to the number of loci that were evaluated for each length?

More discussion could be provided regarding the distribution of proportion of repeats that are mutated in figures 3A, 4A and 4B as the description of figure 4 in the results is a bit confusing.

Several additional programs like ExpansionHunter are also available. Perhaps the authors can discuss why in particular they chose HipSTR and how this differs, or what the relative strengths and weaknesses of this program are relative to other programs.

Figures 1 and 2 have a lot of whitespace, yet the text is small and hard to read. They could be consolidated. 1A and 1B may be better represented as a violin plot.

Figure S1 is helpful in providing visual representation of some of the examples of the authors' findings. De novo #9 and #13 seem a little unclear though, there appear to be 3 alleles; what is the rationale for calling 2 in this case and leave out the reference read (or is the STR evidence at another location in the read)?

**Reviewer 2**

The manuscript by Steely and Watkins et al. analyzes patterns of de novo short tandem repeat (STR or microsatellite) mutations in 3 generation CEPH families. STRs are an important, but understudied, type of genetic variation contributing to variation in complex traits and evolution. They also have complex mutational properties that are still not fully understood, despite years of study. The CEPH families are an amazing genomic resource, and the analysis of genome-wide patterns of STR mutations in these families is excellent. Overall, the authors make a number of interesting, albeit somewhat expected based on existing literature, findings. They estimate that the mutation rate for STRs is ~5.24 x 10^-5, much higher than for SNPs. STRs are enriched in Alu elements. Further, longer motifs tend to have a lower mutation rate than shorter motifs. However, the number of copies of a repeat is positively correlated with the mutation rate. Lastly, most mutations are only single-repeat unit changes, consistent with predictions of the step-wise mutation model.

Overall, I really enjoyed this paper and think that it makes an important contribution to our understanding of genetic variation. While many of the findings are not unexpected, I think their confirmation in a large, genome-wide study is worthwhile and significant. Also, the manuscript and figures are clear and a joy to read, which likely will increase the impact of the paper for a general audience.

I have a number of comments to improve the manuscript:

1) Introduction, Line 5: It's not certain that STRs can always "uniquely identify any person". Maybe rephrase to say, "Typically, only 13 loci are needed to have high-power to distinguish among individuals" or something to that effect.

2) Results, Lines 107-109: Please provide some additional detail (in the Supplement is fine) about how many STRs failed different filters.

3) Results, Lines 124-125: Some additional rationale is needed about why it is required that de novo mutation be seen in two individuals in the third generation. I realize that this requirement will reduce the false-positive rate for de novo calls. However, why 2? Further, what effect will this requirement have on the false-negative rate? Put another way, are there STRs seen in only 1 individual in the third generation that appear to be genuine?

4) Related to above, in lines 126-130, the authors note that 20% of de novo mutations are not found in more than 1 individual and are classified as false positives. What is the evidence that they are false-positives rather than genuine calls that were only transmitted once to the third generation by chance? More clarification here (or in the Methods section) would be helpful.

5) Lines 211-213: For the family that seems to transmit STR mutations at a higher rate, could this family be enriched for false-positive calls? In other words, is there some other bioinformatic or genomic (rather than real biology) attribute of this family that could give the appearance of greater STR mutations?

6) End of Results section: The authors conclude that most mutations are in a single step away from the parent allele. This is consistent with previous empirical and theoretical work. Is there any evidence of a mutational bias toward alleles that increase allele length vs. those that decrease repeat length? This has been suggested in previous studies (e.g. Gymrek et al. 2017 Nature Genetics; Sun et al. 2012 Nature Genetics; Huang et al. 2002 AJHG). In Figure 6, it appears that allele increasing mutations are more likely for the mono through tetranucleotide repeats, but decreasing mutations are slightly more likely for penta- and hexanucleotide repeats. Is this significant?

7) Figure 6: Some of the hexanucleotide mutations are of 2 bp. Could you provide some additional explanation as to what these are? Are they short indels within the repeat unit?

8) Caption for Figure 6: Some of the phrasing here is a bit ambiguous. For C, it says, "De novo contractions and expansions generally decrease in frequency with increasing allele size". I think it would be clearer to say something like "most de novo contractions and expansions are of one repeat unit". Also, for panel d, the authors write, "….the frequency of observed de novo STR alleles decreases exponentially with an increasing number of repeats." There isn't any data presented for this in Figure 6. I realize that SI Figure 4 shows this pattern. So, maybe talk about Figure S4 in the Results section a bit more as I think it's interesting and important. Also, maybe give additional detail in the caption of Figure S4. I think "Negative distribution" should be "exponential distribution"?

9) Lines 258-259: It would be good to explain a little more about how PCR errors could influence the results.

10) It is excellent that the genotype data are available through dbGAP. However, I think it would be extremely useful if the authors could also release a table with the 2859 de novo mutations, giving some basic summary statistics of the mutations. For example, the table could include the genomic location, parental allele, mutant allele, motif etc. I think this summary data would greatly increase the impact and importance of the present paper.

**Authors’ response**

Reviewer #1: Steely and colleagues perform a comprehensive study examining differences mutation rates depending on STR repeat length and testing for age and parent-of-origin effects. They analyze a large cohort of multi-generational pedigrees which permits evaluation and confirmation of repeat slippage across generations and can be compared to a subset of STRs that have already been genotyped in these families. They are able to filter out a high degree of false positives (~20%) and find some individuals with large numbers of mutations. Some of these findings may be due to using samples that were not sequenced in a PCR-free manner.

1) The authors have a good strategy for validating findings in the third generation of samples, but it is unclear whether variability in the first generation is as stringent. For example, what if a grandparent in the first generation was genotyped incorrectly or had a somatic change? How would these false positives be caught?

We appreciate this feedback from the reviewer. We performed an additional analysis comparing the genotypes generated by HipSTR for many of the grandparents to the manually generated genotype dataset described in the manuscript. We find that 796/813 genotypes (from 23 loci) called by HipSTR match the previous dataset for a 97.9% concordance rate. While we believe it is possible that there are a small number of incorrectly called genotypes or somatic mutations, this concordance rate indicates that they are very infrequent. We have added these new genotype comparisons to Table S7, and we have added information about this analysis to the manuscript.

2) The patterns in Figures 3A, 4A and 4B seem a little difficult to explain. The authors provide some rationale but a more formal analysis appears warranted. There seem to be a couple of different factors that contribute: mutations within Alu repeats, and repeat expansion/contractions elsewhere in the genome. The authors could do a more extensive evaluation trying to delineate some of the various possibilities for the distribution. It appears that mono-nucleotide mutations within Alu elements play a substantial role in the distribution of repeat alleles, it would be helpful to determine how much of an effect they have by separating some of the analyses in figures 3 and 4 out between those that are within Alu repeats and those that are not (especially mono- nucleotide repeats in 3A and 4A). Another option would be to list the number of repeats of each overall and pure repeat length (whether or not they are mutated) to get a better sense of this distribution of the denominator and/or list mutations rate individual for A, C, G and T nucleotides. For example, are there many more mono-nucleotide repeats of length ~15, and are these mostly polyA repeats, and are these mostly within Alu elements?

We have analyzed the mononucleotide repeats within Alu elements separately from those that occur outside of Alu elements. We performed this analysis for the longest perfectly repeating segment, as well as for the total repeat length. Figures for these analyses are shown in the Figure S2, and they show that the pattern for the proportion of loci mutated in Alu and non-Alu STRs are quite similar. Additionally, we have added Table S4 for the denominator of each mononucleotide repeat motif (A, T, C, and G). This table shows a substantial decline for most repeat types after approximately 25 bases. It is likely that this decrease in frequency, along with the difficulty in sequencing these simple repeats, explains some of the patterns shown in this table. We have added more discussion on this to the manuscript.

3) Likewise, the data in figure 3B show an expected pattern for pure repeats, but in the comparable plot in figure 4B, the mutation rate value drops off after a repeat length of ~40. Is the rate of interrupted motifs much higher, and does this therefore decrease the possibility of mutation? Also, is there a bias in the nucleotide composition for repeats that have a high mutation rate (e.g. AT-repeats) versus those that have a longer repeat length but a lower frequency of mutation? Or is it again related to the number of loci that were evaluated for each length?

In this case, the decrease in mutation rate is likely caused by a sharp decline in the denominator (number of possible loci that can mutate after an STR length of ~44). For perfect repeats, ~92% of dinucleotide repeats are <=44 bases in length. Additionally, ~80% of all imperfect repeats are <=44 bases (and less than 100bp) in length. With the vast majority of perfect and imperfect repeats being smaller than this cutoff, the number of possible mutations decreases quickly. For total number of repeats (including perfect and imperfect), we find that only ~20% of all dinucleotide repeats are between 45 and 100 bp in length. We have added more discussion on this to the manuscript.

4) More discussion could be provided regarding the distribution of proportion of repeats that are mutated in figures 3A, 4A and 4B as the description of figure 4 in the results is a bit confusing.

We have added information from the above two points to the results and discussion regarding figures 3A, 4A, and 4B (Now figures 2 and 3).

5) Several additional programs like ExpansionHunter are also available. Perhaps the authors can discuss why in particular they chose HipSTR and how this differs, or what the relative strengths and weaknesses of this program are relative to other programs.

We have added additional information to the results regarding our use of HipSTR rather than other programs.

6) Figures 1 and 2 have a lot of whitespace, yet the text is small and hard to read. They could be consolidated. 1A and 1B may be better represented as a violin plot.

We have combined Figures 1 and 2 and replaced Figures 1A and 1B with violin plots.

7) Figure S1 is helpful in providing visual representation of some of the examples of the authors' findings. De novo #9 and #13 seem a little unclear though, there appear to be 3 alleles; what is the rationale for calling 2 in this case and leave out the reference read (or is the STR evidence at another location in the read)?

In these cases, the calls were made based on read counts. For STRs, calls are generally based on the largest and most prevalent reads. PCR of most STRs produces stutter bands that are typically smaller than the actual allele and decrease by number of unit repeats while also decreasing counts. Occasionally, a small number of reads will be larger than the actual allele. The HipSTR counts for these alleles are now given on the image.

Reviewer #2: The manuscript by Steely and Watkins et al. analyzes patterns of de novo short tandem repeat (STR or microsatellite) mutations in 3 generation CEPH families. STRs are an important, but understudied, type of genetic variation contributing to variation in complex traits and evolution. They also have complex mutational properties that are still not fully understood, despite years of study. The CEPH families are an amazing genomic resource, and the analysis of genome-wide patterns of STR mutations in these families is excellent. Overall, the authors make a number of interesting, albeit somewhat expected based on existing literature, findings. They estimate that the mutation rate for STRs is ~5.24 x 10^-5, much higher than for SNPs. STRs are enriched in Alu elements. Further, longer motifs tend to have a lower mutation rate than shorter motifs. However, the number of copies of a repeat is positively correlated with the mutation rate. Lastly, most mutations are only single-repeat unit changes, consistent with predictions of the step-wise mutation model.

Overall, I really enjoyed this paper and think that it makes an important contribution to our understanding of genetic variation. While many of the findings are not unexpected, I think their confirmation in a large, genome-wide study is worthwhile and significant. Also, the manuscript and figures are clear and a joy to read, which likely will increase the impact of the paper for a general audience.

I have a number of comments to improve the manuscript:

1) Introduction, Line 5: It's not certain that STRs can always "uniquely identify any person". Maybe rephrase to say, "Typically, only 13 loci are needed to have high- power to distinguish among individuals" or something to that effect.

We thank the reviewer for this comment and have made the suggested edit.

2) Results, Lines 107-109: Please provide some additional detail (in the Supplement is fine) about how many STRs failed different filters.

We have added Table S1 containing the exact number of STRs that failed different filters. We have also separated mononucleotides from other STR lengths. For built-in HipSTR filters (posterior probability, PCR stutter percentage, and flanking indel percentage), we do not have precise information on which loci were removed for which reason, as the loci that did not pass these filters were removed from the file by HipSTR without clear distinction as to which filter removed each locus.

3) Results, Lines 124-125: Some additional rationale is needed about why it is required that de novo mutation be seen in two individuals in the third generation. I realize that this requirement will reduce the false-positive rate for de novo calls. However, why 2? Further, what effect will this requirement have on the false-negative rate? Put another way, are there STRs seen in only 1 individual in the third generation that appear to be genuine?

We added information to the results about this requirement. Briefly, we followed criteria similar to those used in the original HipSTR publication, which used a single three- generation family for their analysis. We have also included a new supplemental table (Table S1) showing that many of the loci that failed the filtering criteria are mononucleotide repeats. For false-negative results, it is certainly possible that some of these found in a single individual in the third generation could be real events; however, because of the high number of grandchildren in the third generation, we would expect a very small number of false negatives. In an average family with nine grandchildren, assuming Mendelian inheritance, the probability of a de novo mutation being found in zero or only one grandchild is 0.0215 (binomial test). In our 5249 putative de novo mutations, we would expect only 113 false negatives (0.0215*5249). Additionally, this is taken from our total number of identified mutations, some of which would have been removed through other filters. From this, it seems likely that the expected number of false negative mutations would be quite small.

4) Related to above, in lines 126-130, the authors note that 20% of de novo mutations are not found in more than 1 individual and are classified as false positives. What is the evidence that they are false-positives rather than genuine calls that were only transmitted once to the third generation by chance? More clarification here (or in the Methods section) would be helpful.

Some of this was addressed in the above point, but the expected number of false positives would be low, and many of these mutations (~54%; now detailed in a supplemental table) are from mononucleotide repeats, which have been difficult to analyze and are the source of many of our false positives. This has been added to the methods.

5) Lines 211-213: For the family that seems to transmit STR mutations at a higher rate, could this family be enriched for false-positive calls? In other words, is there some other bioinformatic or genomic (rather than real biology) attribute of this family that could give the appearance of greater STR mutations?

This is an interesting point, but we have not found any evidence that this family would be enriched for false positive calls. The coverage and quality metrics for the CEPH genomes are all comparable. Additionally, a high degree of variation in the de novo SNV mutation rate among the CEPH families has been previously observed by Sasani et al., whom we now cite.

6) End of Results section: The authors conclude that most mutations are in a single step away from the parent allele. This is consistent with previous empirical and theoretical work. Is there any evidence of a mutational bias toward alleles that increase allele length vs. those that decrease repeat length? This has been suggested in previous studies (e.g. Gymrek et al. 2017 Nature Genetics; Sun et al. 2012 Nature Genetics; Huang et al. 2002 AJHG). In Figure 6, it appears that allele increasing mutations are more likely for the mono through tetranucleotide repeats, but decreasing mutations are slightly more likely for penta- and hexanucleotide repeats. Is this significant?

We appreciate the reviewer’s observation and clarify the results presented in Figure 6. We now state that the overall trend is for repeat expansion but note by-class differences, “By class, the larger penta- and hexanucleotide repeats had slightly more contractions than expansions, but the differences between expansions and contractions were not significant (Figure 6D, P > 0.5).”

We update the conclusion section and cite the suggested literature, “We also found a general trend for increasing allele length driven by single repeat length expansions in mono- through tetranucleotide repeats, consistent with previous studies (Gymrek et al. 2017; Sun et al. 2012; Huang et al. 2002).”

7) Figure 6: Some of the hexanucleotide mutations are of 2 bp. Could you provide some additional explanation as to what these are? Are they short indels within the repeat unit?

We appreciate the reviewer pointing this out. These were mutations of only 1bp, and we have clarified this in the last paragraph of the Discussion. Upon examining several of these repeats, we found that they were indeed short indels in within the repeat unit. FamilyIDLocationSize changeTypeMotif

1421131012:74816569 31 -> 32deletionCTTTTT

1463220916:30171540 27 -> 26deletionCTTTTT

136285633:17876300 33 -> 32deletionCTTTTT

136285633:53423264 41 -> 40deletionGAAAAA

8) Caption for Figure 6: Some of the phrasing here is a bit ambiguous. For C, it says, "De novo contractions and expansions generally decrease in frequency with increasing allele size". I think it would be clearer to say something like "most de novo contractions and expansions are of one repeat unit". Also, for panel d, the authors write, "....the frequency of observed de novo STR alleles decreases exponentially with an increasing number of repeats." There isn't any data presented for this in Figure 6. I realize that SI Figure 4 shows this pattern. So, maybe talk about Figure S4 in the Results section a bit more as I think it's interesting and important. Also, maybe give additional detail in the caption of Figure S4. I think "Negative distribution" should be "exponential distribution"?

We have changed the caption for Figure 6C to read, “Most de novo events are contractions or expansions of one repeat unit.” Additionally, we have deleted the sentence in the caption for Figure 6D and moved it to the Results section. We have corrected the wording from “negative distribution” to “exponential distribution”.

9) Lines 258-259: It would be good to explain a little more about how PCR errors could influence the results.

We have added the following sentence to the second paragraph of the discussion “PCR stutter may create products that are generally one repeat unit smaller than the target, leading to an incorrect repeat size being sequenced and genotyped.”

10) It is excellent that the genotype data are available through dbGAP. However, I think it would be extremely useful if the authors could also release a table with the 2859 de novo mutations, giving some basic summary statistics of the mutations. For example, the table could include the genomic location, parental allele, mutant allele, motif etc. I think this summary data would greatly increase the impact and importance of the present paper.

We agree with the reviewer and have compiled a table showing the genomic location, parental alleles, the mutated allele, and the motif for each mutation identified in this study. This table is available in the supplement.

**2^nd^ round**

**Reviewer 1**

The authors have responded appropriately to my queries. I have no further comments, congratulations on the manuscript.

**Reviewer 2**

The authors have adequately addressed my comments on the previous version of the manuscript. I really like this paper!

My only remaining very minor comment is that the authors report the probability of a mutation being transmitted 0 or 1 times in a set of 9 children as 0.0215 from a binomial test. However, I believe this probability should be 0.0195. If I’m missing something about how this is calculated, then my apologies. Of course, this difference does not affect the overall excellent point that the authors are making here about the low probability of false negatives.

**Authors’ response**

Reviewer #2: The authors have adequately addressed my comments on the previous version of the manuscript. I really like this paper!

My only remaining very minor comment is that the authors report the probability of a mutation being transmitted 0 or 1 times in a set of 9 children as 0.0215 from a binomial test. However, I believe this probability should be 0.0195. If I’m missing something about how this is calculated, then my apologies. Of course, this difference does not affect the overall excellent point that the authors are making here about the low probability of false negatives.

We inadvertently added probability values evaluated at 0 and 1 which were *already* cumulative. We have corrected the problem and thank the reviewer for catching this error
